# Evolutionary Convergence of C_4_ Photosynthesis: A Case Study in the Nyctaginaceae

**DOI:** 10.3389/fpls.2020.578739

**Published:** 2020-11-02

**Authors:** Roxana Khoshravesh, Matt Stata, Shunsuke Adachi, Tammy L. Sage, Rowan F. Sage

**Affiliations:** ^1^Department of Ecology and Evolutionary Biology, The University of Toronto, Toronto, ON, Canada; ^2^Department of Biology, The University of New Mexico, Albuquerque, NM, United States; ^3^Institute of Global Innovation Research, Tokyo University of Agriculture and Technology, Fuchu, Japan

**Keywords:** *Allionia*, *Boerhavia*, C_4_ photosynthesis, convergent evolution, Nyctaginaceae phylogeny, PEP carboxylase

## Abstract

C_4_ photosynthesis evolved over 65 times, with around 24 origins in the eudicot order Caryophyllales. In the Caryophyllales family Nyctaginaceae, the C_4_ pathway is known in three genera of the tribe Nyctagineae: *Allionia*, *Okenia* and *Boerhavia.* Phylogenetically, *Allionia* and *Boerhavia/Okenia* are separated by three genera whose photosynthetic pathway is uncertain. To clarify the distribution of photosynthetic pathways in the Nyctaginaceae, we surveyed carbon isotope ratios of 159 species of the Nyctaginaceae, along with bundle sheath (BS) cell ultrastructure, leaf gas exchange, and C_4_ pathway biochemistry in five species from the two C_4_ clades and closely related C_3_ genera. All species in *Allionia, Okenia* and *Boerhavia* are C_4_, while no C_4_ species occur in any other genera of the family, including three that branch between *Allionia* and *Boerhavia*. This demonstrates that C_4_ photosynthesis evolved twice in Nyctaginaceae. *Boerhavia* species use the NADP-malic enzyme (NADP-ME) subtype of C_4_ photosynthesis, while *Allionia* species use the NAD-malic enzyme (NAD-ME) subtype. The BS cells of *Allionia* have many more mitochondria than the BS of *Boerhavia*. Bundle sheath mitochondria are closely associated with chloroplasts in *Allionia* which facilitates CO_2_ refixation following decarboxylation by mitochondrial NAD-ME. The close relationship between *Allionia* and *Boerhavia* could provide insights into why NADP-ME versus NAD-ME subtypes evolve, particularly when coupled to analysis of their respective genomes. As such, the group is an excellent system to dissect the organizational hierarchy of convergent versus divergent traits produced by C_4_ evolution, enabling us to understand when convergence is favored versus when divergent modifications can result in a common phenotype.

## Introduction

C_4_ photosynthesis is a complex trait that arises following modifications to hundreds if not thousands of individual genes within a genome (Gowik et al., 2011). Despite this, it is one of the most convergent of evolutionary phenomena in the biosphere, with over 65 independent origins (Conway-Morris, 2003; Sage, 2016; Heyduk et al., 2019). Evolutionary convergence, however, does not necessarily reflect convergence throughout the hierarchy of traits that give rise to a complex phenotype, because multiple mechanisms can support a common function (Losos, 2011). This is well illustrated in the case of C_4_ photosynthesis and crassulacean acid metabolism (CAM), each of which have been repeatedly assembled using disparate enzymes and structural modifications (Sage et al., 2012; Christin and Osborne, 2013; Edwards, 2019). While examples of evolutionary convergence are many, the mechanisms of convergence remain a major question in the life sciences, particularly in the cases where complex traits such as C_4_ photosynthesis repeatedly evolve (Blount et al., 2018). Because the complexity of the C_4_ system is well-understood, as well as the phylogenetic distribution of the many C_4_ clades, C_4_ photosynthesis represents an excellent system to understand the mechanics of convergent evolution, and its implication for the rise of C_4_-dominated biomes over the past 30 million years (Christin and Osborne, 2013; Heyduk et al., 2019).

**FIGURE 1:**
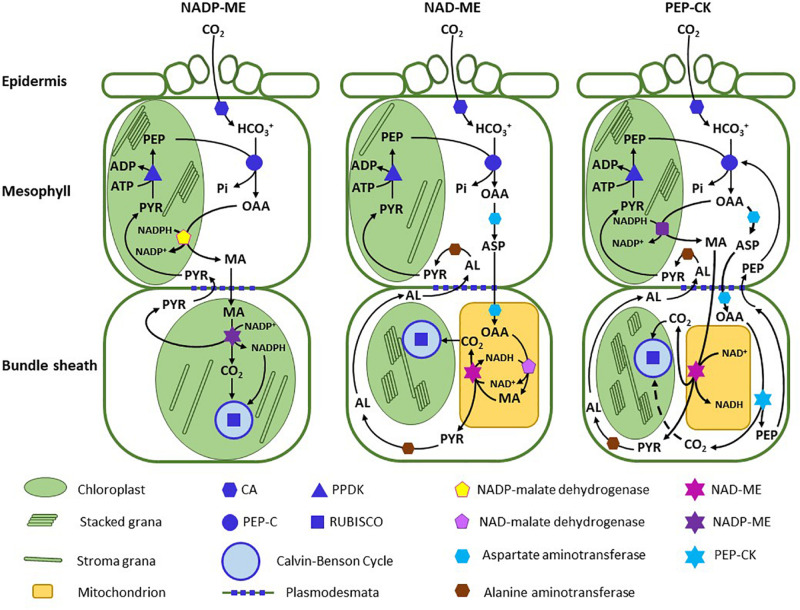
A schematic outlining the C_4_ pathway in NADP-ME type species, NAD-ME species, and PEP carboxykinase species. The diagram represents the classical depiction of the pathway (as per Hatch, 1987 and Furbank, 2011) assuming malate or aspartate predominate in their respective pathways. For clarity, nitrogen exchange reactions between aspartate and pyruvate are not shown in the NAD-ME and PCK subtypes. Thylakoid stacking is indicated by multi-layered rectangles in the chloroplasts. AL, alanine; ASP, aspartate; CA, carbonic anhydrase; MA, malate; OAA, oxaloacetate; PEP, phosphoenolpyruvate; PEP-C, PEP carboxylase; PEP-CK, PEP carboxykinase; PPDK, pyruvate, phosphate dikinase; PVA, pyruvate.

C_4_ photosynthesis first captures CO_2_ at low concentration in an outer mesophyll (M) compartment via the activity of phosphoenolpyruvate (PEP) carboxylase (PEPCase), and then concentrates it into an internal compartment, typically a layer of cells around the leaf vasculature termed the bundle sheath (BS; Edwards and Walker, 1983; Hatch, 1987).^1^ The C_4_ pathway begins with the conversion of CO_2_ to bicarbonate (HCO_3_^–^) by carbonic anhydrase (CA) in M cells, followed by PEP carboxylation (Figure 1). These two steps occur in all C_4_ plants, and thus are universally convergent traits in C_4_ photosynthesis; however, different paralogs have been recruited to carry out the PEPCase and CA functions, demonstrating evolutionary flexibility at a lower level of organization (Christin et al., 2010b, 2013a; Ludwig, 2016a; Heyduk et al., 2019). The product of PEP carboxylation, oxaloacetate (OAA), is too labile to safely move between M and BS cells, so it must be converted to a stable metabolite (Edwards and Walker, 1983). Metabolite transport between M and BS cells is by diffusion, which necessitates that metabolites form steep concentration gradients to support rapid flux, and thus must be stable at high concentration (Bräutigam and Weber, 2011). The solution to the challenge presented by OAA instability is to convert it to the stable metabolites malate or aspartate. This highlights another fundamental feature of evolutionary convergence, in that it occurs where there are strict physiochemical constraints such as OAA lability. A common step (OAA conversion) is accomplished via divergent metabolic solutions (formation of malate versus aspartate). The selected transport metabolite, as it turns out, reflects the enzyme that catalyzes the decarboxylation step.

There are three major decarboxylating enzymes co-opted for the decarboxylation step in C_4_ photosynthesis: NADP-malic enzyme (NADP-ME), NAD-malic enzyme (NAD-ME) and PEP carboxykinase (PCK; Hatch, 1987). Most C_4_ lineages use NADP-ME as the primary decarboxylating enzyme, about 1/3 use NAD-ME and five lineages predominantly use PCK (Sage R. F. et al., 2011). The consequence of the decarboxylase selection affects many aspects of C_4_ photosynthesis, to include the pathway biochemistry, BS and M ultrastructure, M to BS transport processes, leaf energetics, and photosynthetic efficiency (Hatch, 1987; Drincovich et al., 2011; Ghannoum et al., 2011). To recognize the suite of traits associated with each decarboxylation mode, three subtypes of C_4_ photosynthesis have been delineated – the NADP-ME subtype, the NAD-ME subtype, and the PCK subtype (Figure 1). Although each subtype conducts C_4_ photosynthesis, they represent three evolutionary mechanisms to concentrate CO_2_ that are derived from a distinct set of biochemical, structural and transport traits (Rao and Dixon, 2016). While each subtype represents a divergent means of concentrating CO_2_ around Rubisco, traits associated within each subtype reflect strong convergence in response to constraints imposed by the decarboxylating enzyme. The NADP-ME enzyme co-opted by the C_4_ cycle is located in the BS chloroplasts, where it uses malate and NADP^+^ to produce CO_2_, NADPH and pyruvate, with the NADPH directly supporting reduction of PGA produced by Rubisco. NAD-ME is mitochondrial, and uses malate and NAD^+^ to produce CO_2_, NADH and pyruvate. Because it is active in the BS mitochondria, substantial mitochondrial volume is needed to meet the metabolic requirements of the C_4_ pathway, and consistently, species of the NAD-ME subtype often have more and/or larger mitochondria in BS cells than NADP-ME species (Dengler and Nelson, 1999; Edwards and Voznesenskaya, 2011). The PCK enzyme is located in the cytosol, and uses ATP and OAA to produce PEP, CO_2_, and ADP + P_*i*__._ Large amounts of ATP are needed for the PCK reaction, and hence there is a large investment in BS mitochondrial volume to meet the ATP requirement (Yoshimura et al., 2004; Voznesenskaya et al., 2006). The decarboxylation type also constrains the selection of the transport metabolite. In NADP-ME species, NADPH produced by malate oxidation in the BS chloroplast is rapidly consumed in the metabolism of PGA generated by Rubisco, so there is no feedback onto malate flux. In NAD-ME species, the use of malate as a transport molecule would be problematic. NADH cannot readily exit the mitochondria, and there is an insufficient energy sink in the mitochondria to use the large amount of NADH that would be generated if NAD-ME oxidized malate imported from the M cells. The utilization of aspartate avoids these issues because there is no net import of reducing power into the BS mitochondria (Kanai and Edwards, 1999). Once in the BS mitochondria, aspartate forms OAA, which is reduced to malate via malate dehydrogenase using the NADH generated by NAD-ME (Figure 1).

PEP carboxykinase directly uses OAA, and hence to avoid flooding the BS cytosol with reducing power, aspartate is also imported from M cells. However, the PCK reaction requires large amounts of ATP to support rapid photosynthesis, and this can be generated in the mitochondria by oxidizing NADH created by NAD-ME using malate directly imported into the BS mitochondria from the M cells (Leegood and Walker, 1999). PEP carboxykinase species are thought to use NAD-ME at about a fourth to a third of the rate of the PCK reaction, in order to supply sufficient NADH for ATP production (Leegood and Walker, 1999). Consistently, PCK species have many mitochondria in the BS (Dengler and Nelson, 1999).

To meet the energy requirements imposed by the decarboxylating enzymes and associated transport systems, a distinct arrangement of chloroplast membranes occurs in each of the three subtypes (Hatch, 1987; Edwards and Voznesenskaya, 2011; Rao and Dixon, 2016). NADP-ME species have low grana stacking in the BS chloroplasts, yet high stacking in the M chloroplasts. More grana stacking in M cells increases the PSII content in the thylakoids, and hence the potential to generate NADPH by linear electron transport. Because NADP-ME species import reducing power into the BS with malate, the demand for NADPH to reduce PGA in the BS chloroplasts of NADP-ME plants is halved.; hence less PSII and granal stacking are required (Kanai and Edwards, 1999). Chloroplasts in the M cells of NADP-ME species are well stacked to support malate reduction following PEP carboxylation. NAD-ME species, by contrast, have low grana stacking in M chloroplasts because their primary energetic function is to produce ATP for PEP regeneration. The thylakoids of the BS exhibit pronounced stacking in NAD-ME species, to supply sufficient NADPH to potentially reduce all the PGA generated by the C_3_ cycle. However, there can be variation on these general chloroplast phenotypes, depending upon the degree to which multiple decarboxylases are employed, whether PGA is exported to the M tissue for reduction, or if aspartate is imported into the chloroplast in lieu of malate, as suggested to occur in NADP-ME *Flaveria* species (Leegood and Walker, 1999; Furbank, 2011).

The theoretical pattern that emerges in C_4_ evolution is thus one of universal convergence in terms of overall outcome (CO_2_ concentration into a BS-like compartment), with some steps being universal (CO_2_ conversion to bicarbonate, PEP carboxylation), and others being divergent (decarboxylation, transport, chloroplast energetics, and possibly how PEP is regenerated). Convergence occurs within the subtype pathways where specific biophysical constraints are present, for example, in transport metabolites associated with the distinct decarboxylating enzymes. An organizational hierarchy between convergence and divergence can thus be envisioned for complex traits that scales from the level of the genes up through to the composite phenotype. The challenge for research on convergent evolution is to describe this hierarchy and to understand why and under what constraints convergent patterns emerge (Losos, 2011). To do this, one needs effective research systems, such as fast cycling microbes, or in terrestrial plants, multiple lineages where convergence has occurred, such as the dozens of clades that have independently evolved C_4_ photosynthesis (Blount et al., 2018; Heyduk et al., 2019). However, most closely related C_4_ clades are of the same subtype (Sage R. F. et al., 2011), which restricts the ability to examine sub-type impacts on convergent versus divergent solutions to creating a C_4_ pathway. One potentially strong study system occurs in *Portulaca*, where both NADP-ME and NAD-ME species are present (Voznesenskaya et al., 2010; Ocampo et al., 2013). Another possibility occurs in the Nyctagineae tribe of the Nyctaginaceae. Here, three related genera of the tribe Nyctagineae - *Allionia*, *Boerhavia* and *Okenia* - contain C_4_ species while their closest relatives in the genera *Anulocaulis*, *Cyphomeris, Commicarpus*, and *Nytctaginia* are not known to have any C_4_ species (Sage R. F. et al., 2011). In the phylogeny of Douglas and Manos (2007), *Okenia* and *Boerhavia* form a common C_4_ clade, while *Allionia* forms a distinct C_4_ clade that is separated from the *Okenia/Borehavia* lineage by the *Anulocaulis*/*Nyctaginia* complex. However, there has been no systematic survey of the occurrence of C_3_ and C_4_ photosynthesis in the Nyctaginaceae, so it is unclear whether C_4_ may exist in *Anulocaulis, Commicarpus, Cyphomeris*, and *Nyctaginia*, or even whether *Allionia*, *Okenia*, and *Boerhavia* are completely C_4_, as opposed to also containing C_3_ and C_3_–C_4_ intermediate species. *Boerhavia* and *Allionia* are listed as being NADP-ME, although this is based on enzyme assays for *Boerhavia* only (Muhaidat et al., 2007). Intriguingly, our preliminary TEM images show many mitochondria in the BS ultrastructure of *Allionia*, suggesting it is NAD-ME. If so, then the *Allionia* and *Okenia/Boerhavia* clade could join *Portulaca* in forming a robust study system for addressing evolutionary convergence as influenced by C_4_ subtype.

To evaluate the potential of the Nyctaginaceae to become a model for studying evolutionary convergence, we present here a detailed study of the distribution of the C_3_ and C_4_ pathways in the Nyctaginaceae. We present a survey of carbon isotope ratios from 560 herbarium specimens, in addition to an anatomical/ultrastructural study using five representative species of *Allionia*, *Anulocaulis*, *Boerhavia*, *Commicarpus*, and *Nyctaginia*. We also examine climate data and geographic distributions of species in *Allionia*, *Anulocaulis*, *Boerhavia*, *Commicarpus*, *Cyphomeris*, and *Nyctaginia* to evaluate ecological factors contributing to C_4_ origins in the Nyctaginaceae. The biochemical sub-types of C_4_ species in *Allionia* and *Boerhavia* were determined, and we present a transcriptome-based phylogeny that updates the phylogenies of Douglas and Manos (2007) and Douglas and Spellenberg (2010). We also use transcriptome data to evaluate whether there has been convergence in the gene sequences of the major C_4_ pathway enzymes. For comparative purposes, we also present TEM images of leaf tissues from two *Portulaca* (Portulaceae) species previously shown to be NADP-ME (*Portulaca pilosa*) or NAD-ME (*Portulaca oleracea*). Through these efforts, we present an initial hierarchical assessment of how divergence occurs during the convergent evolution of C_4_ photosynthesis.

## Materials and Methods

### Plant Material and Growth Conditions

Seeds of *Allionia incarnata*, *Anulocaulis gypsogenus*, *Boerhavia burbigeana*, *Boerhavia coccinea*, *Commicarpus scandens, and Nyctaginia capitata* were collected from naturally occurring field populations in Southwestern North America and Australia between 2000 and 2016 (see Supplementary Table 1 for collection and voucher information). *Portulaca pilosa* seeds were a gift from Gerry Edwards and Elena Vosnesenskaya (Washington State University), while *P. oleracea* grew as a weed in our greenhouse. Seeds were sown directly into 10 or 20 L pots containing a sandy-loam mixture and grown in the University of Toronto greenhouse complex housed on the roof of the Earth’s Sciences Centre. Plants were watered as needed to avoid soil drying (daily in high summer, two to three times weekly in cooler weather), and fertilized bimonthly with a Miracle-Grow commercial fertilizer mix (All-Purpose Brand, 24-8-16) amended with 4 mM calcium nitrate and 1 mM magnesium sulfate. Greenhouse conditions were 27 to 33°C daytime temperature, 20–24°C night temperature, and a peak photon flux density (PFD) of 1500 μmol photons m^–2^ s^–1^ on clear days. We used 400 W sodium vapor lamps to supplement natural daylight to maintain a minimum PFD on cloudy days of 250 μmol m^–2^ s^–1^ over a >13 h photoperiod. Unless otherwise indicated, three to five plants were sampled for gas exchange, biochemical assay, leaf structural properties and transcriptomics between May and October of 2010 to 2017. Care was taken to sample tissues under the same environmental conditions in the greenhouse (full sun exposure, a leaf temperature near 27°C, at a time between 9 am and 2 pm) to minimize year to year and month to month variation. For the characteristics examined here, subtle variation that may exist between sampling dates is not known to affect results pertinent to our hypotheses. For example, C_3_ versus C_4_ expression patterns are largely constitutive, and sun to shade variation in phenotype would not be present at the bright light intensities present in our greenhouses during summer.

### Carbon Isotope Ratio

To determine the distribution of the C_3_ and C_4_ pathways in species of the Nyctaginaceae, 560 herbarium specimens representing 23 genera and 159 species were sampled from the herbaria at Kew gardens (Richmond, London, United Kingdom), Missouri Botanical Gardens (St Louis, MO United States), and New York Botanical Gardens (The Bronx, New York, NY, United States; Supplementary Tables 2, 3). Species were sampled from all genera in the Nyctagineae with the exception of the monospecific genus *Cuscatlainia*. Approximately 50% (*Mirabilis*) to 100% (*Okenia, Allionia*) of the known species from the sampled Nyctagineae genera are present in the survey. We also sampled species from 12 genera occurring in each of the other six tribes of the Nyctaginaceae (Table 1 and Supplementary Table 2). To measure the carbon isotope ratio (δ^13^C) of each herbarium specimen, 2–4 mg of leaf or stem material were sampled from herbarium sheets and assayed for δ^13^C by the Washington State University Stable Isotope Core.^2^ δ^13^C was determined for at least two distinct plant specimens if available in the herbaria. Carbon isotope ratios between −10 and −16‰ correspond to C_4_ values, while carbon isotope ratios between −23 and −32‰ correspond to C_3_ values. Species that are evolutionary intermediates between C_3_ and C_4_ photosynthesis exhibit δ^13^C ratios that are similar to C_3_ values except where a strong C_4_ metabolic cycle has been engaged; such C_4_-like plants typically exhibit δ^13^C values between −16 and −22‰ (Monson et al., 1988; Von Caemmerer, 1992).

**TABLE 1:** δ^13^C data for sampled species from the Nyctaginaceae showing number of accepted species for each genus and the number of species sampled, the range of δ^13^C for species means and number of C_4_ species we identified.

Tribe and genus	Accepted species number/sampled species number	δ^13^C range of species means	Number of C_4_ species in Supplementary Table 2
**Boldoeae**			
*Salpianthus* Humb. & Bonpl.	5/3	−28.8 to −27.4	0
**Bougainvilleeae**			
*Belemia* Pires	1/1	non-Kranz anatomy	0
*Phaeoptilum* Radlk.	1/1	−24.4	0
**Caribeeae**			
*Cryptocarpus* H.B.K.	1/1	−27.2	0
**Colignonieae**			
*Colignonia* Endl.	6/6	−28.7 to −25.3	0
**Leucastereae**			
*Andradea* Allemão	1/1	−26.0	0
*Leucaster* Choisy	1/1	−28.8	0
*Ramisia* Glaz. ex Baillon	1/1	−24.3	0
**Nyctagineae**			
*Abronia* Juss.	24/20	−29.5 to −24.5	0
*Acleisanthes* A. Gray	17/14	−28.0 to −23.9	0
***Allionia* L.**	**2/2**	**−13.3 to −13.2**	**2**
*Anulocaulis* Standl.	5/5	−28.0 to −24.2	0
***Boerhavia* L.**	**40/43**	**−14.9 to −9.4**	**43**
*Commicarpus* Standl.	25/23	−28.7 to −25.2	0
*Cyphomeris* Standl.	2/2	−28.1 to −27.2	0
*Mirabilis* L.	54–60/23	−29.8 to −22.6	0
*Nyctaginia* Choisy	1/1	−26.2	0
***Okenia* Schldl. & Cham.**	**1/4**	**−14.0 to −12.3**	**4**
*Tripterocalyx*	4	27.8 to 24.9	0
**Pisonieae**			
*Pisoniella* (Heimerl) Standl.	1/1	−25.8	0
*Grajalesia* Miranda	1/1	−25.7	0
*Cephalotomandra* Karst. & Triana	1	−28.8	0

*C_4_ genera are highlighted in bold. See Supplementary Tables 2,3 for herbarium specimens, collection information and individual δ^13^C values. Accepted species numbers follows Spellenberg, 2003, Tropicos (2020), and the International Plant Names Index (IPNI, 2020). Sampled species number includes species listed in Supplementary Tables 2,3 whose acceptability is uncertain. *Belemia fucsiodes* is a type specimen examined with a dissecting scope for vein density only.*

### Leaf Gas Exchange and Biochemical Assay

The response of net carbon assimilation rate (*A*) to intercellular CO_2_ concentration (*C*_*i*_) was measured for *Allionia incarnata*, *B. coccinea*, and *N. capitata* using a Li-COR 6400 photosynthetic gas analyzer at 33°C, a vapor pressure differences of 2.0–2.5 kPa and a photosynthetic PFD of 1800 μmol m^–2^ s^–1^. The most recent, fully mature leaves were first equilibrated in the leaf cuvette at an ambient CO_2_ concentration of 400 μmol mol^–1^ and 1800 μmol photons m^–2^ s^–1^. After equilibration and measurement, the ambient CO_2_ was increased to 1200 μmol mol^–1^ and then gas exchange parameters were measured after equilibration. The CO_2_ concentration was then reduced to near the CO_2_ compensation point in approximately 13 steps, with steady-state gas exchange values determined at each step. The initial slope of the *A versus C_*i*_* response was estimated as the linear slope of the measurements below a *C*_*i*_ of 100 μmol mol^–1^. Intrinsic water use efficiency (WUE) was determined as the ratio of *A* to stomatal conductance (*g*_*s*_) at an ambient CO_2_ of 400 ppm, and the CO_2_-saturated rate of *A* (*A*_1200_) was measured at 1200 μmol mol^–1^ CO*_2_*.

The activities of PEPC, NADP-malic enzyme, and NAD-malic enzyme were assayed at 30°C using a coupled-enzyme assay that measured oxidation/reduction rate of NADP(H) or NAD(H) at a wavelength of 340 nm using a Hewitt-Packard 8230 spectrophotometer following procedures in Ashton et al. (1990) as modified by Sage T. L. et al. (2011). Two to three cm^2^ of recent, fully-mature leaves of *A. incarnata, B. coccinea*, and *N. capitata* were sampled under full illumination in the greenhouse and then rapidly ground using a glass tissue homogenizer in an extraction buffer (100 mM HEPES – pH 7.6, 5 mM MgCl_2_, 10 mM KHCO_3_, 2 mM EDTA, 10 mM 6-aminocaproic acid, 2 mM benzamide, 1 mM phenylmethylsulfonyl fluoride, 1% (w/v) PVPP, 2% (w/v) PVP, 0.5% Triton X-100, 2% (w/v) BSA, 5 mM DTT, 1% (w/v) casein). After removing two aliquots for chlorophyll assay (in 80% acetone at 645 and 663 nm; Arnon, 1949), the extract was centrifuged 30 s and divided into three aliquots and put on ice. The aliquot used for NAD-ME assay was immediately treated with sufficient MnCl_2_ to give a 2 mM solution. PEPC was assayed by coupling the production of OAA to NADH oxidation via malate dehydrogenase in an assay buffer containing 50 mM Bicine (pH 8.0), 1 mM EDTA, 5 mM MgCl_2_, 2 mM NaHCO_3_, 2 mM DTT, 1 mM glucose 6-phosphate, 2 units ml^–1^ malate dehydrogenase, and 0.2 mM NADH. The reaction was initiated by the addition of PEP to give 5mM. NADP-ME was assayed by following NADP^+^ reduction at 340 nm. The assay buffer contained 25 mM Tricine (pH 8.2), 1 mM EDTA, 20 mM MgCl_2_, 2 mM DTT, 0.5 mM NADP. The reaction was initiated by the addition of malic acid to give 5 mM. NAD-ME was assayed by measuring NAD^+^ reduction at 340 nm. The reaction mixture contained 25 mM Hepes (pH 7.2), 0.2 mM EDTA, 8 mM ammonium sulfate, 1 unit ml^–1^ malate dehydrogenase, 5 mM malic acid, 0.025 mM NADH, and 2 mM NAD^+^. The reaction was initiated by adding MnCl_2_ to give 5 mM.

### Imaging and Quantification of Leaf Structure

For all anatomical and ultrastructural imaging, the middle portion of a leaf blade equidistant between the mid-rib and leaf margin were sampled from recent, fully expanded leaves. Leaf pieces approximately 2 mm^2^ were collected between 9:00–11:00 am and were prepared for light and transmission electron microscopy as described previously (Khoshravesh et al., 2017). Cell and organelle features of BS cells were quantified using Image J software (Schneider et al., 2012). One leaf per plant was sampled from three to five plants. Each mean value per plant was compiled from measurements of 5–10 imaged cells from the adaxial region of the leaf. All measurements were conducted on planar images of cross (=tranverse) sections. Cells measured were randomly selected from the pool of cells where the plane of section passed through the central region of a BS cell, rather than the periphery. Parameters measured include BS cell area; number of chloroplasts per BS cell; number of mitochondria per BS cell; area of individual chloroplasts and mitochondria; and% BS cell area covered by all chloroplasts and mitochondria in a BS cell.

### Phylotranscriptomic Analysis

To prepare a phylotranscriptome of the Nyctaginaceae, we used publicly availably RNA-sequence data from the NCBI Short Read Archive (Supplementary Table 4).^3^ FASTQ read data was trimmed using Trimmomatic (Bolger et al., 2014) with minimum leading and trailing quality cutoffs at 30, a 5-base sliding window cutoff of 30, and 70bp minimum trimmed length. Transcriptomes were *de novo* assembled using Trinity (Grabherr et al., 2011) with default settings including *in silico* read normalization. Open reading frames were predicted and translated using the getorf program from the EMBOSS package (Rice et al., 2000), and the longest open reading frame for each putative locus identified by Trinity was selected using a Python script. OrthoFinder (Emms and Kelly, 2015) was used to predict groups of orthologous genes using 10 reference proteomes with MapMan annotations (Thimm et al., 2004) and 10 with all other annotation types available from the Phytozome v12 (Supplementary Table 5).^4^ Single- or low-copy orthogroups were selected with a Python script based on the following criteria: a minimum of 40 of 53 species present; no more than 10% of the species having multiple sequences; and a minimum alignment length of 100 amino acids. For species with multiple sequences, a Python script was used to concatenate all orthogroup sequences from a given species if none overlapped by more than 10% of the shortest sequence length, or remove them all if they did. Sequences were assumed to represent fragmented assemblies in the former case and paralogs in the latter. This selection process resulted in 1084 genes for phylogenetic analysis. Initial protein alignments were produced with mafft (Katoh and Standley, 2013) and DNA codon alignments were generated from these using pal2nal (Suyama et al., 2006). The codon alignments were trimmed using trimAl (Capella-Gutiérrez et al., 2009) with a gap threshold of 0.5, then concatenated into a partitioned super-matrix using a Python script written by co-author M. Stata. Phylogenetic inference was conducted using RaxML (Stamatakis, 2014). This was conducted using separate evolutionary models for all partitions, rapid bootstrap analysis with a search for the best tree in one run (command line option -f a), and bootstrap convergence testing (autoMRE). Convergence testing showed that 50 bootstrap replicates were sufficient, but in order to be certain of support values we conducted 200. The species tree was visualized using FigTree^5^. All Python scripts are available at github.com/MattStata/Nyctaginaceae_Scripts.

### C_4_ Gene Phylogenies and Analysis

All homologs of PEPC, NADPME, NADME, and PPDK were identified in the Nyctaginaceae transcriptomes in the NCBI Short Read Archive (Supplementary Table 4) using BLASP with *Arabidopsis* sequences as queries. Matches were aligned using mafft (Katoh and Standley, 2013) and preliminary trees were inferred with FastTree (Price et al., 2010) in order to identify the number of copies present in Nyctaginaceae. For each copy, sequences from the Nyctagineae clade were extracted from the alignment and a consensus sequence was generated using Geneious (www.geneious.com) to create full-length consensus references for each copy onto which RNA-seq data for all Nyctagineae species in the NCBI SRA were mapped using HiSat2 (Kim et al., 2019) with the scoring argument – score-min L,0,-1.4. Read mappings were scrutinized manually using the software Geneious to be certain there were no additional paralogs beyond those we had detected, which would be evident as paralogous reads mapped onto the closest reference. Consensus sequences based on mapped reads were generated in Geneious. Gene trees were inferred using MrBayes (Huelsenbeck and Ronquist, 2001) with two runs, 40 chains, 2 million generations, and a heating factor of 0.05. About 10,000 trees from the end of each run where the average SD of split frequencies remained flat at or below 0.01, were used to generate the final tree and infer posterior probabilities using the consense program included with ExaBayes (Aberer et al., 2014). Trees were visualized using FigTree.^5^ Gene expression values were calculated as reads per kb of transcript per million reads (RPKM) using the SAM files produced by HiSat and a Python script. Only species from the 1KP project^6^ submitted by our lab were used for gene expression as these represent both C_4_ lineages and a closely-related C_3_ outgroup, and were grown and sampled identically. For these, the newest fully expanded leaves were sampled during a sunny day from plants grown in a glass house at the University of Toronto between 9 am and 1 pm. Because complete mappings to all transcripts were not conducted, numbers of total sequenced reads were used in RPKM calculations rather than total mapped reads. Scripts written by M Stata are available at github.com/MattStata/Nyctaginaceae_Scripts.

### Species Distribution Data

Biogeographic distributions for species of the tribe Nyctagineae were obtained from the Global Biodiversity Information Facility website (GBIF).^7^ Duplicate data points and those lacking herbarium records or corresponding to marine coordinates were removed (maptools, Bivand and Lewin-Koh, 2013). The remaining 15,870 observations represented 75 species within *Allionia* (two species), *Boerhavia* (40 species), *Anulocaulis* (five species), *Commicarpus* (25 species), *Cyphomeris* (two species), and *Nyctaginia* (one species). Bioclimate and monthly minimum and maximum temperature parameters (2.5 min resolution) were downloaded from the WorldClim 2.0 dataset^8^ (Fick and Hijmans, 2017). Monthly potential evapotranspiration and Global Aridity Indexes (AI) were downloaded from CGIAR-CSI GeoPortal.^9^ Values per observation for 19 bioclimatic variables in the Worldclim dataset, plus minimum and maximum temperatures and AI were then extracted using the extract function in the R raster package (Hijmans and van Etten, 2012). Median values per variable per species were calculated and normalized to Z-scores for use in subsequent analyses. Climate variables that significantly predicted the occurrence of photosynthetic type at *p* < 0.05 were selected using stepwise regression. Mixed-effect models (R package lme4, Bates et al., 2015) were built using the selected bioclimatic variables and photosynthetic subtypes (NAD-ME or NADP-ME) as the main effect and genus and species as random effects. These models were compared by ANOVA and Akaike’s Information Criteria (AIC). The best model was selected based on the lowest AIC and *p* values <0.05. A principal component analysis was performed by R package FactoMineR (Lê et al., 2008) to evaluate species distribution across the multivariate predictors. A subset of the data for which we had phylogenetic data was also used to run a phylogenetically corrected ANOVA using phytools in R (Revell, 2012).

## Results

### Carbon Isotope Ratios

For the δ^13^C survey, we sampled herbarium specimens from all genera of the Tribe Nyctagineae, except for one monospecific genus from El Salvador (*Cuscatlainia vulcanicola*; Table 1). All sampled species from the genera *Allionia, Boerhavia*, and *Okenia* exhibited C_4_ δ^13^C values (−9 to −15‰) (Table 1 and Supplementary Tables 2,3). All other species exhibited C_3_ δ^13^C values (−22 to −30‰), including all assayed species in the Nyctagineae genera *Abronia, Acleisanthes, Anulocaulis, Commicarpus, Cyphomeris, Mirabilis*, and *Tripterocalyx.* The δ^13^C survey provides little evidence for C_3_–C_4_ intermediate species in the Nyctagineae. Values from all species were clearly C_4_ or within the more negative range of δ^13^C values typical of C_3_ plants, with two possible exceptions – the arid zone species *Acleisanthes angustifolia* (δ^13^C = −23.9) and *Mirabilis polyphylla* (δ^13^C = −22.6). While high for a typical C_3_ δ^13^C value, these exceptions are within the range of values observed in arid-zone C_3_ species with high WUE (Farquhar et al., 1989).

### Gas Exchange and Biochemistry

**FIGURE 2:**
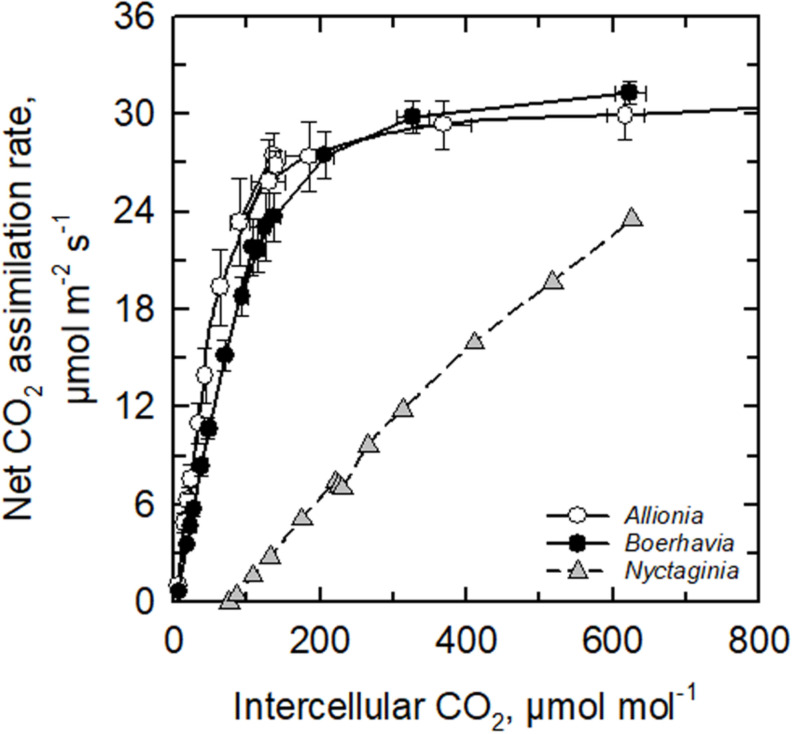
The response of the net CO_2_ assimilation rate to intercellular CO_2_ concentration in *Allionia incarnata* (C_4_), *Boerhavia coccinea* (C_4_) and *Nyctaginia capitata* (C_3_) at 30°C and a light intensity of 1800 μmol photons m^–2^ s^–1^. Means ± SE, N = 4 (for the C_4_ species) or 1 (for the C_3_ species).

Both *Allionia incarnata* and *B. coccinea* exhibited typical C_4_ photosynthetic parameters. The CO_2_ compensation point of photosynthesis (Γ) was below 5 μmol mol^–1^ in both species, the ratio of intercellular to ambient CO_2_ concentration (*C_*i*_/C_*a*_*) ratio was 0.31–0.35, and their carboxylation efficiency of photosynthesis, measured as the initial slope of the photosynthetic response to intercellular CO_2_ concentration (*A*/*C*_*i*_ response), was 5–7 times greater than the carboxylation efficiency in the C_3_ *N. capitata* (Figure 2 and Table 2). *Allionia* exhibited a steeper initial slope of the *A/C_*i*_* response than *Boerhavia.* When relativized by dividing by the maximum net CO_2_ assimilation rate at high CO_2_ (*A*_1200_) to correct for variation in photosynthetic capacity, the normalized carboxylation efficiency in *Allionia* was 50% greater than in *Boerhavia* (Table 2).

**TABLE 2:** Summary of gas exchange and biochemical data for three Nytaginaceae species.

Parameter	*Nyctaginia capitata*	*Boerhavia coccinea*	*Allionia incarnata*	C_4_ *p* value (one-tailed)
Net CO_2_ assimilation rate at 400 μmol CO_2_ mol^–1^ air, in μmol m^–2^ s^–1^	7.0	23.0 ± 2.1	26.8 ± 0.9	0.08
Net CO_2_ assimilation rate at 1200 μmol CO_2_ mol^–1^ air, in μmol m^–2^ s^–1^	23.5	31.3 ± 0.7	30.5 ± 1.5	0.32
*A*_400_/*A*_1200_	0.30	0.74 ± 0.06	0.88 ± 0.03	**0.04**
*A*/*g*_*s*_, mmol CO_2_ mol^–1^ H_2_O	100	158 ± 3	149 ± 5	0.10
*C_*i*_/C_*a*_*	0.58	0.31 ± 0.01	0.35 ± 0.02	0.10
Initial slope of the *A vs C*_*i*_ curve, mol m^–2^ s^–1^	0.05	0.25 ± 0.03	0.36 ± 0.04	**0.04**
Initial slope/A_1200_	0.002	0.008 ± 0.001	0.012 ± 0.001	**0.03**
CO_2_ compensation point of *A*, μmol mol^–1^	75	4.5 ± 0.5	2.5 ± 1.0	0.06
PEP carboxylase activity, μmol m^–2^ s^–1^	15.1 ± 1.9	111.8 ± 7.9	131.7 ± 22.2	0.22
PEP carboxylase activity, mmol mol^–1^ CHL s^–1^	27.0 ± 2.7	261.9 ± 12.4	429.7 ± 53.2	**0.01**
NADP-ME activity, μmol m^–2^ s^–1^	0 ± 0	50.1 ± 5.6	0 ± 0	**< 0.001**
NADP-ME activity, mmol mol^–1^ CHL s^–1^	0 ± 0	116.7 ± 8.9	0 ± 0	**< 0.001**
NAD-ME activity, μmol m^–2^ s^–1^	0 ± 0	2.1 ± 0.6	27.7 ± 2.8	**< 0.001**
NAD-ME activity, mmol mol^–1^ CHL s^–1^	0 ± 0	5.1 ± 1.4	91.5 ± 6.4	**< 0.001**
Chlorophyll, mmol m^–2^	0.56 ± 0.02	0.43 ± 0.02	0.30 ± 0.01	**< 0.001**
Chlorophyll a/b ratio	3.46 ± 0.07	4.45 ± 0.09	3.67 ± 0.08	**< 0.001**

*Means ± SE, N = 4 for the C_4_ species, 1 for *N. capitata* gas exchange data, and 3 for *N. capitata* biochemical data. The *p*-values of one-tailed Students T-tests between the two C_4_ species are indicated, with significant differences highlighted in bold. Gas exchange measurements were conducted at 33°C and a light intensity of 1800 μmol m^–2^ s^–1^. Biochemical assays were conducted at 30°C. *A*, net CO_2_ assimilation rate; *C*_*i*_, intercellular CO_2_ concentration; *C*_*a*_, ambient CO_2_ concentration in the leaf chamber; *A_400_, A* at a CO_2_ concentration of 400 μmol CO_2_ mol^–1^ air; *A_1200_, A* at a CO_2_ concentration of 1200 μmol mol^–1^; CHL, leaf chlorophyll content; *g*_*s*_, leaf conductance to water vapor; ME, malic enzyme.*

The *in vitro* activity of PEPC was high in the C_4_ species relative to the C_3_ *N. capitata*, and similar on a leaf area basis in the two C_4_ species; however, on a chlorophyll basis, the PEPC activity is 63% higher in *Allionia* than *Boerhavia* (Table 2). NADP-ME activity was high in *Boerhavia* and not detected in *Allionia*, while NAD-ME activity was negligible in *Boerhavia* and high in *Allionia* (Table 2). Chlorophyll content was 43% higher in *Boerhavia* than *Allionia*, and chlorophyll *a/b* ratio was 21% higher in *Boerhavia*. Observed enzyme activities are considered robust because they are equivalent to or greater than the observed *A* values in each species. From these results, we conclude that *Boerhavia* belongs to the NADP-ME subtype, while *Allionia* is of the NAD-ME subtype.

### Leaf Structure and Ultrastructure in *Allionia, Boerhavia* and Two *Portulaca* Species

**FIGURE 3:**
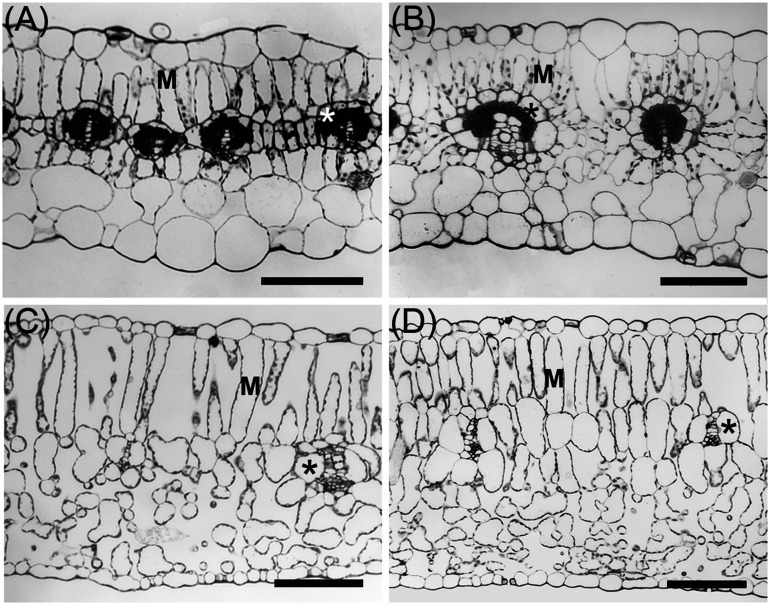
Light microscope images of leaves from C_3_ and C_4_ species of Nyctaginaceae, tribe Nyctagineae. **(A)** *Allionia incarnata*, **(B)** *Boerhavia coccinea*, **(C)** *Commicarpus scandens*, **(D)** *Nyctaginia capitata.* M, mesophyll cells; *Asterisks mark bundle sheath cells. Scale bars = 100 μm.

**FIGURE 4:**
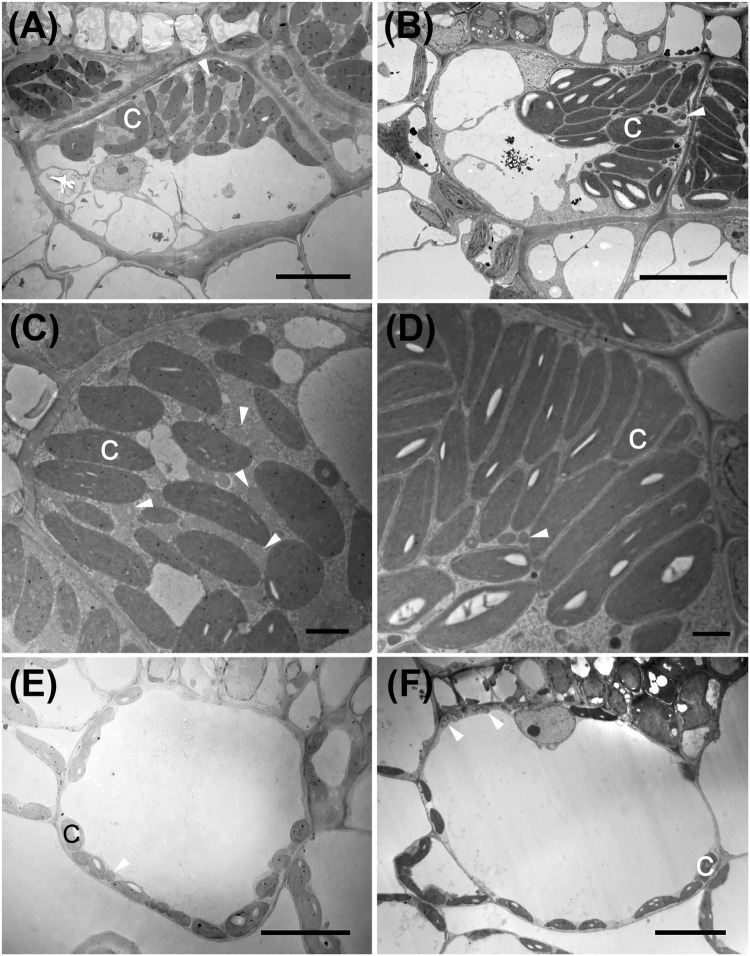
Transmission electron micrographs illustrating bundle sheath cells of C_3_ and C_4_ Nyctaginaceae species from the tribe Nyctagineae. **(A–D)** C_4_ species; **(A,C)** *Allionia incarnata*, **(B,D)***; Boerhavia coccinea*; **(C,D)** high magnification images of the bundle sheath cells. Note high numbers of mitochondria in *Allionia incarnata* **(C)**. **(E,F)** The C_3_ species *Commicarpus scandens* **(E)**, and *Nyctaginia capitata* **(F)**. C, chloroplast; arrowheads mark mitochondria. Scale bars = 10 μm.

*Allionia incarnata* and *B. coccinea* exhibited typical C_4_-Atriplicoid Kranz anatomy, which is characterized by large BS cells in planar cross sections with centripetal organelle arrangements (Figures 3, 4; Edwards and Voznesenskaya, 2011). Notably, enlarged BS cells in both *Allionia* and *Boerhavia* do not wrap around the entire vascular bundle, mimicking patterns observed in *Atriplex* but not many other C_4_ eudicots classified as having Atriplicoid Kranz anatomy (Muhaidat et al., 2007). *A. gypsogenus*, *C. scandens, N. capitata*, and *Mirabilis jalapa* have characteristic C_3_ leaf anatomy (Figures 3, 4 and Supplementary Figure 1). Planar areas of the BS cells are comparable between the C_3_ and C_4_ species, indicating similar dimensions of the BS tissue in a radial direction with respect to the vasculature (Table 3). Both C_4_ species exhibit greater coverage of the BS cell by chloroplasts than the C_3_ species, because there are more chloroplasts per BS cell and greater mean area per chloroplast in the C_4_ species (Figure 4; Table 3). In *A. incarnata*, the planar area of BS cells covered by mitochondria is significantly greater than in C_3_ species and the C_4_ *B. coccinea*, due to the presence of larger mitochondria in planar section, and more mitochondria per cell area (Table 3; Figures 4A,C). The percent mitochondrial coverage of the BS cells is similar in *B. coccinea* and the C_3_ species (Table 3; Figures 4B,D–F).

**TABLE 3:** Ultrastructural parameters for the bundle sheath cells in cross section of two C_4_ species and three C_3_ species from the Nyctagineae tribe of the Nyctaginaceae.

	*A. incarnata*	*An. gypsogenus*	*B. coccinea*	*C. scandens*	*N. capitata*
	C_4_	C_3_	C_4_	C_3_	C_3_
Cell area, μm^2^	427 ± 169 a	727 ± 339 a	557 ± 183 a	505 ± 199 a	944 ± 389 a
% Cell covered by chloroplast,%	28.3 ± 5.9 b	2.3 ± 0.9 a	35.2 ± 11.5 b	13.8 ± 4.6 a	6.5 ± 4.2 a
% Cell area covered by mitochondria,%	3.6 ± 1.7 b	0.4 ± 0.2 a	1.2 ± 0.5 a	0.6 ± 0.3 a	0.5 ± 0.4 a
Number of chloroplasts per cell	16 (5–24) b	5 (2–11) a	18 (3–37) c	10 (7–15) b	10 (5–17) b
Number of mitochondria per cell	45 (12–111) c	14 (4–57) a	28 (4.8–75) b	13 (5–30) a	11 (7–52) a
Area per chloroplast, μm^2^	8.3 ± 3 c	2.9 ± 1.1 ab	11.4 ± 3.8 c	6.1 ± 2.1 bc	5.3 ± 1.2 bc
Area per mitochondria, μm^2^	0.7 ± 0.2 b	0.3 ± 0.1 a	0.4 ± 0.1 a	0.5 ± 0.2 ab	0.3 ± 0.2 a

*Means ± SE, or median ± range for organelle numbers. N = 3–5. Letters besides values indicate statistically distinct groups by Kruskal Wallis test followed by a Dunn’s test except for organelle numbers which are evaluated using a Poisson regression for count data (number of organelles per cell). Abbreviations: *A*, *Allionia*; *An, Anulocaulis; B, Boerhavia, C, Commicarpus; N*, *Nyctaginia*.*

**FIGURE 5:**
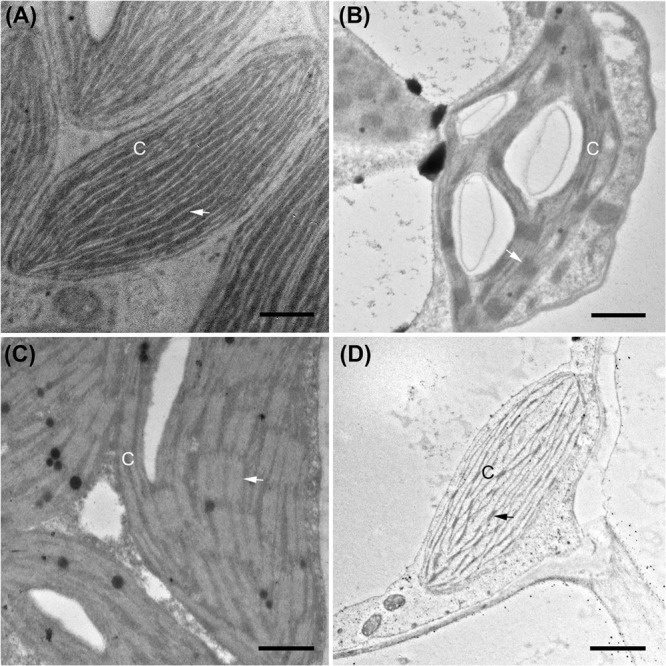
Transmission electron micrographs illustrating chloroplast fine structure. **(A)** *Boerhavia burbidgeana* bundle sheath cell; **(B)** *B. burbidgeana* mesophyll cell; **(C)** *Allionia incarnata* bundle sheath cell; **(D)** *A. incarnata* mesophyll cell. In panels **(A,C)**, thylakoids are lighter stacks and striations against a dark stroma. In panels **(B,D)**, thylakoids are dark stacks and striations against a lighter-staining stroma. Scale bars, 0.5 μm. C, chloroplast; arrows mark stacked thylakoids. Scale bars = 0.5 μm.

The BS cells in species with C_3_ δ^13^C values have smaller and fewer chloroplasts than the C_4_ species of the Nyctagineae, and these are positioned mostly along the outer BS wall exposed to intercellular airspace (Figures 4E,F and Supplementary Figure 1). None of the three C_3_ species examined exhibit traits associated with C_3_–C_4_ intermediacy or even the incipient intermediate state termed “proto-Kranz”; there was not a greater mitochondrial number, nor a repositioning of mitochondria and chloroplasts toward the inner side of the BS cells. Chloroplasts in the BS of *Boerhavia* and the M tissue of *Allionia* are largely agranal, whereas distinct levels of grana stacking are apparent in the thylakoids of the BS chloroplasts in *Allionia*, and the M chloroplasts of *Boerhavia* (Figure 5)

**FIGURE 6:**
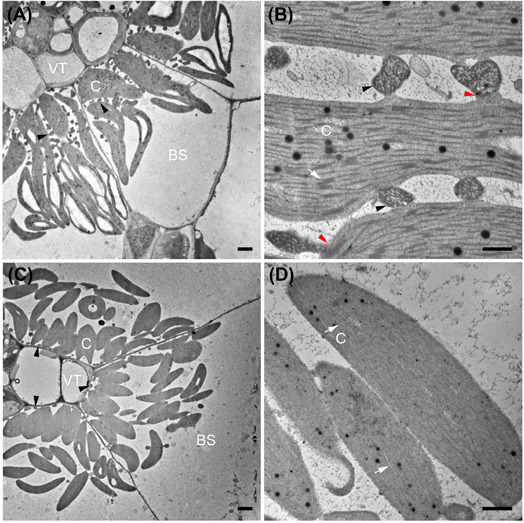
Transmission electron micrographs of bundle sheath cells **(A,C)** and bundle sheath chloroplasts **(B,D)** from NAD-ME subtype *Portulaca oleracea* **(A,B)** and NADP-ME subtype *Portulaca pilosa* **(C,D)**. Red arrowheads mark mitochondria to chloroplasts connection structures in *P. oleracea* **(B)**. BS, bundle sheath; C, chloroplast; VT, vascular tissue; black arrowheads mark mitochondria; white arrows mark thylakoids. Bars = 2 μm **(A,C)** or 0.5 μm **(B,C)**.

In *P. oleracea* (NAD-ME), chloroplasts with pronounced grana stacks cluster in the inner BS (Figure 6A). Many mitochondria are interspersed between the chloroplasts, and exhibit distinct structural connections with nearby chloroplasts (Figure 6B). In *P. pilosa* (NADP-ME), chloroplasts also occur in the inner BS where they form clusters with no discernable thylakoid stacks (Figures 6C,D). Mitochondria are largely absent between chloroplasts, although some mitochondria occur along the inner wall of the BS in a pattern that is commonly observed in C_3_–C_4_ intermediate species (Figure 6C).

### Species Phylogeny

**FIGURE 7:**
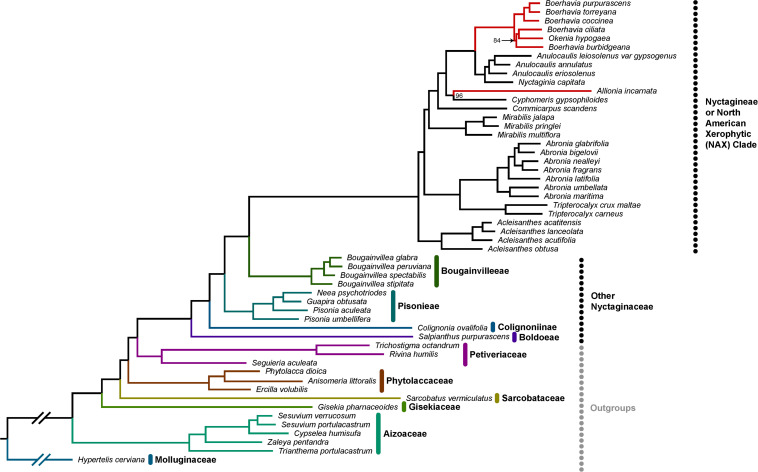
Phylotranscriptomic reconstruction of Nyctaginaceae. A maximum likelihood phylogeny of the Nyctaginaceae family and six outgroup families with previously uncertain topology. This tree is based on a concatenated super-matrix of 847 codon-aligned single-copy orthologs totaling 834,018 sites. All bootstrap support values are 100% except for the two nodes with values indicated, based on 200 bootstrap replicates. Within the Nyctagineae (identified as the North American Xerophytic clade by Douglas and Spellenberg, 2010), C_4_ lineages are denoted with red branches. The tree is rooted on *Hypertelis cerviana* and gapped branches here were shortened for visual convenience.

We update the molecular phylogeny of the Nyctagineae using transcriptome sequence data available at the NCBI short read archive (Figure 7). The tree largely replicates the phylogenies of Douglas and Manos (2007) and Douglas and Spellenberg (2010) showing the C_4_ and C_3_ clades of the Nyctagineae correspond to a clade of xerophytic herbs and shrubs that Douglas and Spellenberg term the North American Xeric (NAX) clade. C_4_ *Allionia* branches with *Cyphomeris* at the base of a clade that includes the isotopically C_3_ *Anulocaulis* and *Nyctaginia* species, and the C_4_ *Okenia* and *Boerhavia* species. *Anulocaulis* and *Nyctaginia* form a clade that branches between *Allionia/Cyphomeris* and *Boerhavia/Okenia. C. scandens* branches at the base of the clade containing the C_4_ species and their immediate non-C_4_ sister clades, with *Mirabilis* species branching just below *C. scandens*. Unlike Douglas and Spellenberg (2010) who predicted Bougainvilleeae + Pisonieae to be sister to the Nyctagineae, we predict only Bougainvilleeae branches in a sister position, with maximal support. Also, the branching order of Phytolaccaceae, Sarcobataceae, Gisekiaceae, and Nyctaginaceae is unclear in the literature and in the APG Caryophllalyes tree, where they form a polytomy.^10^ Our tree resolves them with 100% support, improving the phylogenetic context for the family.

### Gene Phylogenies

**FIGURE 8:**
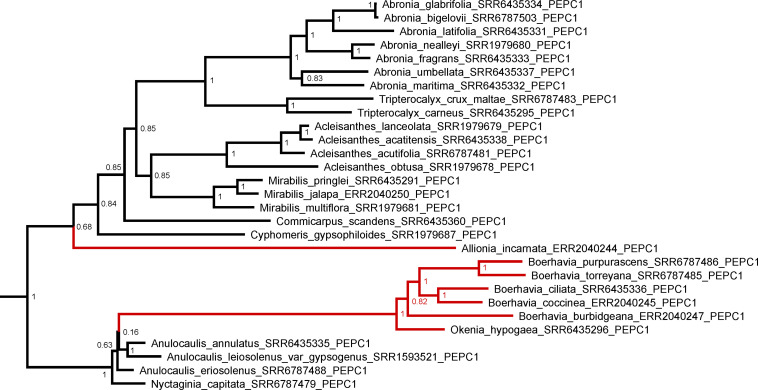
A Baysian gene phylogeny for the PEPCase gene copy co-opted for C_4_ function in the Nyctaginaceae. Node values are posterior probabilities. Red branches denote C_4_ species. A tree with all three PEPC paralogs is presented in Supplementary Figure 2.

We examined the trees of four important C_4_ cycle genes – PEPCase, NADP-ME, NAD-ME, and PPDK (Figure 8 and Supplementary Figures 2–5). For each gene, we assumed that the functional copy in the C_4_ pathway was the one with the highest expression in the transcriptome analysis. In this analysis, we numbered gene copies based on branching order from the base of the gene tree; the numbers assigned are not meant to imply direct orthology with gene copies from any other lineage. For *Allionia incarnata* and two *Boerhavia* species, the *PEPC1* gene is the copy used in the C_4_ pathway because it shows an expression strength that is orders of magnitude greater than *PEPC2 and PEPC3* (Table 4). By similar logic, *NAD-ME3* is the main decarboxylase gene in the C_4_ pathway of *Allionia*, and *NADP-ME2* is the main decarboxylase gene in *Boerhavia. Allionia* also exhibited significant expression of *NADP-ME1*, approaching that of *NAD-ME3* (Table 4). The expression of the PEP carboxykinase gene was minimally detectable in both C_4_ clades (not shown). In the gene trees for *PEPC1*, *PPDK* and *NADP-ME1*, the respective orthologs from numerous C_3_ species of the Nyctagineae branch between the two C_4_ lineages, with strong support (Figure 8 and Supplementary Figures 2–5). This indicates the C_4_ genes arose independently from an ancestral C_3_ copy, rather than by lateral transfer between clades. If one or more of the genes had moved laterally between the C_4_ clades, the orthologs from both C_4_ lineages would form a common clade.

**TABLE 4:** RNA Transcript Expression of C_4_ cycle enzymes from four selected species of the Nyctaginaceae.

	PEPC1	PEPC2	PEPC3	NAD1	NAD2	NAD3	NADP1	NADP2	NADP3
*Mirabilis jalapa* (C_3_)	164	19	105	50	28	4	122	4	75
*Allionia incarnata* (C_4_)	12069	33	88	80	25	2001	1751	114	13
*Boerhavia burburgiana* (C_4_)	17742	1	144	56	6	0.4	8563	2	144
*Boerhavia coccinea* (C_4_)	9331	0.1	128	85	27	0.2	3562	2	121

*Yellow shading indicates the expression values of gene copies that are predominant in the C_4_ metabolic cycle. Green shading indicates enhanced expression of genes for which the activity *in vitro* was negligible. PEPC, PEP carboxylase; NADP, NADP malic enzyme; NAD, NAD-malic enzyme. Data were generated from transcriptomes in the 1KP database (www.onekp.com) and are available in the NCBI Short Read Archive (Supplementary Table 4). PEP carboxykinase transcript levels were found to be negligible in each species.*

### Positively Selected Amino Acid Substitutions

We examined *PEPC1* sequences for evidence of convergence in the C_4_ isoforms (Table 5). Christin et al. (2008) showed that 21 amino acid sites were under positive selection in the *PEPC1* of various C_4_ grasses, with the best-known example of sequence convergence being a serine for alanine substitution near the maize 780 position in the PEPC1 sequence (position 774 in the *Flaveria* sequence; Gowik and Westhoff, 2011). We did not find evidence for convergence with other C_4_ lineages at position 780 in *Allionia* nor *Boerhavia*, as they both exhibited an alanine, which is typical of C_3_ isoforms (Table 5 and Supplementary Figure 6). There was consistent convergence at sites 572, 761, and 807, where all C_4_ species exhibited the same amino acids as those under positive selection in C_4_ grasses. Convergence was observed in both C_4_ lineages at site 813, but in only one of the six *Boerhavia/Okenia* species in the database (Table 5). *Boerhavia* also converged on the same amino acids as the C_4_ grass species at site 733 and 863, and partially at position 502 (*B. purpurescens and B. torreyana* only). In total, of the 21 C_3_ to C_4_ amino acid switches repeatedly observed in the grass PEPCase sequences (Christin et al., 2007), about a third were replicated in the *Boerhavia* lineage and a fifth in the *Allionia* lineage (Table 5). At one site, position 577, the C_3_ and C_4_ Nyctaginaceae species share a serine with the C_4_ grass *Zea mays*. In the grasses, most C_3_ species examined by Christin et al. (2007, 2012b) exhibit an alanine at this site. It is possible that C_4_ evolution in Nyctaginaceae did not require this substitution as the ancestral C_3_ state was already a serine.

**TABLE 5:** Comparison of amino acids in the C_4_ PEP carboxylases of the *Allionia* and *Okenia/Boerhavia* C_4_ clades with PEP carboxylase sites under positive selection in C_4_ grasses.

	Sites with posterior probability > 0.999												Prob > 0.99			Prob > 0.95			
Clade	466	517	531	560	577	579	625	637	761	780	794	807	572	599	813	665	733	863	866
*Boerhavia/Okenia*	Met	Thr	Ala	Arg	Ser	Ala	Leu	Met	Ala	Ala	Phe	Lys	Gln	Ile	Arg, Gln*	His	Met	Lys	Glu
*Allionia*	Met	Thr	Ala	Arg	Ser	Ala	Val	Met	Ala	Ala	Phe	Lys	Gln	Ile	Gln	His	Phe	Asn	Glu
C_3_ Nyctag	Met	Thr	Ala	Arg	Ser	Ala	Val	Met	Ser	Ala	Phe	Arg	Glu	Ile	Arg	His	Phe	Asn	Glu
C_4_ grasses (Christin et al. (2007)	Ile, Val	Ala, Cys	Pro	Pro	Ser	Glu	Ala	Leu, Phe	Ala	Ser	Val	Lys	Gln	Val	Gln	Asn	Met, Val	Lys	Glu

Site numbers correspond to the maize PEP carboxylase gene (PEPC). Sites under positive selection were identified by a screen of grass lineages examined by Christin et al. (2007). Posterior probabilities noted with blue headers are from that study. Sites showing an identical amino acid substitution in either or both C_4_ Nyctaginaceae lineages are highlighted in yellow. We list the most frequent positively selected amino acid(s) at each site in C_4_ grasses. The species in the C_3_ Nyctag clade are the North American Xerophytic C_3_ species in the *PEPC1* gene tree in Supplementary Figure 2, which exhibited identical amino acids at the sites shown. *Gln is only present in *B. burbidgeana*; the other *Boerhavia* and *Okenia* have Arg at position 813.

### Ecological Distribution

Nyctagineae species from the genera *Allionia, Boerhavia, Anulocaulis, Commicarpus, Cyphomeris*, and *Nyctaginia* are distributed in tropical and subtropical regions of similar climate (Supplementary Figures 7–9). Six bioclimatic variables significantly predicted (*p* < 0.05) the C_3_ and C_4_ distribution using a stepwise regression model (Supplementary Figure 8 and Supplementary Table 6). To address whether the variation in distribution of C_3_ and C_4_ species could be species or genera dependent, GLMM models were performed with and without considering taxa as random effects. The model was significantly improved (*p* < 2e^–16^) when genus was added as the random effect (Supplementary Table 6); however, after this addition, none of the main effects significantly predicted the pattern of photosynthetic pathway distribution. A phylogenetically-corrected ANOVA also failed to detect a significant difference between the C_3_ and C_4_ species in response to six variables (Supplementary Table 7). These analyses show that the distribution of Nyctagineae species in climate and geographic space follows taxonomic and phylogenetic affinities rather than photosynthetic pathway.

## Discussion

Carbon isotope ratios demonstrate that all examined species in *Allionia*, *Boerhavia* and *Okenia* are C_4_, while all examined species in other Nyctaginaceae genera are not, including species in *Anulocaulis*, *Commicarpus*, *Cyphomeris*, and *Nyctaginia* that branch sister to the C_4_ clades. The number of C_4_ species in the Nyctaginaceae is 43–46 based on recent assessments that conclude there are 40 *Boerhavia*, one to four *Okenia*, and two *Allionia* species (Spellenberg, 2003; Tropicos, 2020). The typically C_3_ δ^13^C values in the 31 examined species of *Anulocaulis*, *Commicarpus*, *Cyphomeris*, and *Nyctaginia* indicate that none of the examined species have a type of C_3_–C_4_ intermediacy termed C_4_-like, where a strong C_4_ metabolic cycle has been engaged. As a C_4_ cycle becomes engaged, the values of δ^13^C increase from C_3_ toward C_4_ values, becoming distinct from typical C_3_ values when δ^13^C rises above −22‰ (Von Caemmerer, 1992; Alonso-Cantabrana and von Caemmerer, 2016). This occurs because PEPCase does not discriminate against the ^13^C isotope to the degree that Rubisco does (Farquhar et al., 1989). Carbon isotope screens cannot differentiate between C_3_ plants and a type of C_3_–C_4_ intermediacy utilizing C_2_ photosynthesis, a metabolic pathway that concentrates CO_2_ into the BS using photorespiratory metabolites to shuttle CO_2_ from M to BS cells. In C_2_ photosynthesis, the photorespiratory enzyme glycine decarboxylase is expressed only in BS mitochondria, which forces photorespiratory glycine to diffuse from the M cells where it is formed to the BS cell for metabolism (Sage et al., 2012). The released CO_2_ from glycine decarboxylation accumulates in the BS cells, where it can be refixed by Rubisco in adjacent chloroplasts with high efficiency. C_2_ photosynthesis improves carbon gain at low CO_2_ concentrations, but because all CO_2_ is fixed by Rubisco, the δ^13^C values of C_2_ plants reflect those of C_3_ plants (Von Caemmerer, 1992). Anatomically, C_2_ plants have increased numbers of mitochondria and chloroplasts in BS cells, typically in a centripetal position against the BS wall facing the vascular tissue (Khoshravesh et al., 2016). This characteristic identifies candidate C_2_ species, with low CO_2_ compensation points of photosynthesis (Γ) confirming the presence of the C_2_ physiology. Our examination of the BS structure in *Anulocaulis*, *Commicarpus* and *Nyctaginia*, and leaf gas exchange in *Nyctaginia*, showed no evidence of C_2_ photosynthesis as the structural characteristics and Γ were typically C_3_. Hence, we conclude that the sister clades to the *Allionia* and *Boerhavia/Okenia* clades are most likely comprised of only C_3_ plants. Gas exchange data and anatomical studies demonstrate strong C_4_ features in selected species of *Allionia* and *Boerhavia*, which along with the consistently C_4_ values of δ^13^C in these clades, lead us to conclude they are completely C_4_ rather than C_4_-like. C_4_-like species have a fully functional C_4_ cycle but retain limited C_3_ photosynthesis in M tissues, and exhibit features suggesting they are newly evolved C_4_ plants that have not yet optimized the C_4_ pathway (Moore et al., 1989).

In the phylogeny, *Anulocaulis* and *Nyctaginia* species occur in a clade that branches sister to the *Boerhavia/Okenia* clade, while *Cyphomeris* branches sister to *Allionia*, supporting a hypothesis of two independent C_4_ origins, one in ancestral *Allionia* and a second in ancestors to the *Okenia/Boerhavia* clade. An alternative possibility is a single C_4_ origin followed by reversions to C_3_ in ancestors of *Cyphomeris* and the *Anulocaulis/Nyctaginia* clade. This option is less parsimonious, requiring three changes – one acquisition of C_4_ and two reversions – rather than two. It is also not favored because reversions are considered unlikely and have never been confirmed (Christin et al., 2010a; Oakley et al., 2014; Bräutigam and Gowik, 2016). In addition, the two C_4_ clades exhibit different biochemical and structural subtypes. The high degree of structural, transport, and biochemical specialization associated with each subtype indicates switching subtypes would be difficult, and consistently, no instances of subtype switching have been documented. Gene sequences of C_4_ pathway enzymes can also be used to assess reversal possibilities. If a reversion had occurred, the derived ortholog of a C_4_-pathway enzyme in any C_3_ progeny might retain sequences from their ancestral C_4_ function (Christin et al., 2010a; Khoshravesh et al., 2020). In *Anulocaulis*, *Cyphomeris*, and *Nyctaginia*, the C_4_-type orthologs of PEPCase, PPDK, and both decarboxylases exhibited no signatures of prior C_4_ function. For example, the C_3_ species in the Nyctagineae share only one positively selected amino acid substitution with the C_4_ Nyctaginaceae and *Zea mays* in the PEPC1 sequence, a serine at position 577. C_3_ species outside the clade where an ancestral C_4_ transition could have occurred also share this serine, indicating it is not a relic from a C_4_-state. Based on these points, we conclude that *Allionia* and *Boerhavia* represent independent yet closely related C_4_ clades that have diverged in terms of biochemical subtype. With other examples of subtype divergence in closely related species of *Portulaca* and possibly other clades (*Salsola* and Sesuvioideae; Voznesenskaya et al., 1999; Bohley et al., 2015), comparative methods of evolutionary biology can be used to address questions of convergence and divergence during C_4_ evolution.

In contrast to a prior conclusion that both *Boerhavia* and *Allionia* are NADP-ME (Muhaidat et al., 2007), the biochemical assays demonstrate species in these two clades are different biochemical subtypes of C_4_ photosynthesis. Consistently, *Allionia* and *Boerhavia* showed pronounced differences in BS and M ultrastructure that support their designation as NAD-ME and NADP-ME subtypes, respectively. In both *B. coccinea* (high NADP-ME activity, low NAD-ME activity) and *A. incarnata* (high NAD-ME activity, nil NADP-ME activity), chloroplasts and mitochondria occupy the inner half of the BS cells, as is typical in eudicot C_4_ species (Edwards and Voznesenskaya, 2011). In *A. incarnata*, there is an abundance of mitochondria scattered among the elongated chloroplasts, while in *B. coccinea*, BS mitochondria are much less frequent and not commonly interspersed within the chloroplast cluster. In NAD-ME species, the juxtaposition of chloroplasts and mitochondria facilitates rapid movement of CO_2_ released from mitochondria into adjacent chloroplasts, while the tight packing of chloroplasts and mitochondria reduces the chance of CO_2_ escape. This tight packing is a major means by which CO_2_ is trapped in species lacking a suberized layer around the BS wall, as is the case in eudicots (von Caemmerer and Furbank, 2003). In *Portulaca* species, the same patterns hold but with one important variation that is not reported in the literature, notably, mitochondria in the BS of NAD-ME *P. oleracea* form distinct attachment structures to adjacent chloroplasts that are not obvious in *Allionia* and other NAD-ME type C_4_ species (for example, *Anticharis*, *Cleome, Salsola*, and *Suaeda;* Voznesenskaya et al., 1999, 2018; Khoshravesh et al., 2012). Such structures may facilitate rapid CO_2_ diffusion between mitochondria and chloroplasts, enhancing refixation efficiency.

The structural characteristics observed here are consistent with patterns in other C_4_ clades. NADP-ME species among C_4_ grasses, sedges and eudicots (as shown in *Flaveria*, *Euphorbia*, *Gomphrena, Heliotropium*, *Salsola*, and *Tribulus*) share with *Boerhavia* and *Portulaca pilosa* the pattern of enlarged chloroplasts with weakly developed grana stacks, and few BS mitochondria (Carolin et al., 1978; Kim and Fisher, 1990; Ueno, 1996, 2013; Voznesenskaya et al., 1999; Yoshimura et al., 2004; Muhaidat et al., 2011; Sage T. L. et al., 2011; Lauterbach et al., 2019). NAD-ME eudicots in *Amaranthus*, *Atriplex*, *Anticharis*, *Cleome*, *Gisekia*, *Salsola, Suaeda*, and *Tecticornia* share with *Allionia* and *P. oleracea* the pattern of enlarged chloroplasts with well-developed grana and large numbers of interspersed mitochondria (Carolin et al., 1978; Voznesenskaya et al., 1999, 2007, 2008; Khoshravesh et al., 2012; Bissinger et al., 2014; Oakley et al., 2014). In grasses, Dengler and Nelson (1999) describe NAD-ME species as having 5-to 20-fold more mitochondria than NADP-ME species, and NAD-ME mitochondria are often larger with greater internal membrane surface area in the BS. The differences between C_4_ subtypes hold even when different tissue layers are co-opted as the site of CO_2_ concentration, whether it is the mestome sheath as in grasses, an inner chlorenchyma layer as occurs in *Salsola* and other succulent chenopods, or single-cell type of C_4_ photosynthesis as shown in the NAD-ME chenopods *Bienertia cycloptera* and *Suaeda aralocaspica* (Ueno, 1996, 2013; Voznesenskaya et al., 1999; Pyankov et al., 2000; Edwards and Voznesenskaya, 2011; Khoshravesh et al., 2020). In the middle of M cells of *Bienertia cycloptera*, for example, there are many mitochondria within a ball of Rubisco-containing chloroplasts where CO_2_ is concentrated (Voznesenskaya et al., 2002). The central chloroplasts in *B. cycloptera* have well-stacked grana, while peripheral chloroplast that are functionally equivalent to M chloroplasts of typical C_4_ species do not.

Lateral transfer of genes encoding C_4_ pathway elements has been observed in closely related grass clades within *Alloteropsis* and *Neurachne* (Christin et al., 2012a,b; Dunning et al., 2017; Khoshravesh et al., 2020). Lateral gene transfer is relevant to discussions of evolutionary convergence because the acquisition of previously evolved C_4_ genes by a non-C_4_ relative could facilitate C_4_ evolution in the receptive clade, possibly creating a false impression of convergence. When we examined the phylogenies of C_4_ pathway genes in the Nyctagineae, we found no evidence of lateral transfer. The gene trees consistently showed the two C_4_ lineages do not share a common gene copy of a C_4_-adapted isoforms of *PEPC*, *PPDK*, or either malic enzyme, because the respective orthologs from related C_3_ species branch between the C_4_ lineages with high support (Figure 8 and Supplementary Figures 2–5). This evidence is consistent with a hypothesis that the two C_4_ clades in the Nyctaginaceae arose *de novo* from a completely C_3_ state, rather than via assisted origins involving gene introgression from a pre-existing C_4_ clade (Christin et al., 2012a,b; Dunning et al., 2017).

With sequencing data becoming widely available, it may be possible to identify C_4_-subtypes using transcriptomics (Lauterbach et al., 2019). Such an approach, however, could produce errors if not coupled with enzyme activity assays. Here, the transcriptomes consistently showed high expression of the copies we designate as *NADP-ME1* in *Boerhavia* and *NAD-ME3* in *Allionia*. *Allionia* also exhibited significant expression of *NADP-ME1* transcripts which approach expression of its *NAD-ME3* gene. This data by itself would suggest co-function of NADP-ME and NAD-ME in *Allionia*; however, no NADP-ME activity was detected in *Allionia*, leading us to conclude its *NADP-ME1* transcripts are not translated into functional enzyme. The possibility of error in this conclusion is unlikely because the BS ultrastructure in *Allionia* is consistent with patterns generally seen in NAD-ME subtypes. The high transcript level of both NAD-ME and NADP-ME may be a relic from ancestral *Allionia* species that may have utilized both decarboxylases during an early phase of C_4_ photosynthesis. A chance selection event may have subsequently enhanced NAD-ME activity, after which leaf structure and energetics were optimized for the NAD-ME subtype.

One of the notable features of C_4_ photosynthesis is that it is concentrated in relatively few orders of higher plants. The Poideae (grasses, sedges) and Caryophyllales, for example, account for over 90% of all C_4_ species and about 50 of the estimated 65 independent origins of C_4_ photosynthesis (Sage, 2016). The ability of these clades to repeatedly evolve the C_4_ pathway is hypothesized to reflect the presence of enabling traits that facilitate C_4_ evolution (Sage, 2004; Christin et al., 2013b). Wider BS cells in C_3_ species, for example, could be a structural enabling trait (Muhaidat et al., 2011; Christin et al., 2013b; Griffiths et al., 2013), while greater numbers of organelles in BS tissues may reflect an activation of photosynthetic physiology which enables establishment of photorespiratory glycine shuttles (Sage et al., 2013; Schulze et al., 2013). Photosynthetic activation of the BS can facilitate the rise of photorespiratory glycine shuttles because mitochondria and some chloroplasts can reposition to the inner BS region, forcing glycine formed in centrifugal chloroplasts to migrate to the inner BS for processing by glycine decarboxylase (Sage et al., 2013). To evaluate whether enabling traits are present in close C_3_ relatives of the C_4_ Nyctaginaceae clades, we examined their BS structure and organelle characteristics. In the C_3_ species of *Anulocaulus, Cyphomeris*, and *Nyctaginia*, BS cells had similar cross-sectional areas as the C_4_ BS cells, and exhibited numerous chloroplasts along the outer wall, indicating photosynthetic activation. We thus conclude the close C_3_ relatives of *Allionia* and *Boerhavia* exhibit numerous enabling traits for C_4_ evolution, indicating they were present in ancestral C_3_ taxa from which the C_4_ clades arose.

### C_4_ Selection Environments

The results indicate C_4_ photosynthesis evolved in Nyctaginaceae species from hot and dry climates of the New World, most likely in the arid-to-semi-arid regions of Southwestern North America where the center of diversity occurs for the North American Xerophytic clade of the Nyctaginaceae (Douglas and Manos, 2007). Christin et al. (2011) estimate that the C_4_ pathway appeared in the *Boerhavia/Okenia* clade about 4.7 million years ago, and 6.1 million years ago in *Allionia*, based on molecular phylogenies. This was during a period of aridification in Southwestern North American and reduced atmospheric CO_2_ (Sage et al., 2018). The environmental habitat of *Allionia* versus *Boerhavia* did not differ much from each other or their C_3_ ancestors, indicating the C_3_ ancestors were adapted to the same kind of hot, dry environments where the C_4_ species are common. This supports a hypothesis that C_4_ arose in these sorts of environments, consistent with a model that high levels of photorespiration brought on by heat, drought and/or salinity promoted C_4_ evolution in C_3_ plants pre-adapted to such harsh environments (Sage et al., 2018).

Surveys of the floristic distribution of NADP-ME versus NAD-ME grasses have identified a trend where NADP-ME species predominate in grass floras of wetter climates while NAD-ME species predominate in drier climates (Vogel et al., 1978; Hattersley, 1992; Ghannoum et al., 2011). The reasons for this trend have not been clarified, although it has been hypothesized that the pattern reflects phylogenetic ancestry where NAD-ME species are more likely to arise in clades from drier areas while NADP-ME species evolve in wetter climate zones (Taub, 2000). Explanations for habitat differences between C_4_ subtypes can be evaluated using closely related NADP-ME and NAD-ME clades. In *A. incarnata* and *B. coccinea*, we observed similar photosynthetic capacities and intrinsic WUE (*A/gs*), indicating no obvious differences in photosynthetic parameters that might explain subtype segregation along a moisture gradient. *Allionia* did exhibit a steeper initial slope of the *A/Ci* response than *Boerhavia*, indicating a stronger C_4_ metabolic cycle in the NAD-ME plant. Consistently, the NAD-ME clade in *Allionia* had greater PEPCase activity per unit chlorophyll than *Boerhavia* (NADP-ME clade). If a stronger metabolic cycle is an inherent feature of NAD-ME relative to NADP-ME species, there could be a photosynthetic advantage under low intercellular CO_2_ concentrations that commonly occur where drought and low humidity reduce stomatal aperture. A comparative analysis using closely related species of differing subtype in the Nyctagineae, *Portulaca* and other lineages could evaluate this possibility.

### Convergence Versus Non-convergence in the C_4_ Functional Type

As a highly convergent, complex trait, C_4_ photosynthesis represents an excellent system to dissect evolutionary convergence, particularly the degree of convergence versus divergence in the mix of traits that give rise to a composite phenotype (Heyduk et al., 2019). Numerous studies have previously examined convergent properties of C_4_ components, such as convergence in genes for C_4_ enzymes (Christin et al., 2007, 2009, 2010b; Emms et al., 2016); regulatory components (Gowik and Westhoff, 2011; John et al., 2014; Reyna-Llorens and Hibberd, 2017), structural features (Yoshimura et al., 2004; Kadereit et al., 2014; Stata et al., 2014; Danila et al., 2018); and biochemical sub-types (Gutierrez et al., 1974; Hatch et al., 1975; Sage R. F. et al., 2011; Ludwig, 2016b). However, the assembly of multiple datasets into a hierarchical framework has not been attempted in a C_4_ context. In Table 6, we present a preliminary hierarchy of convergent and divergent traits observed in comparative studies of the many C_4_ clades, thereby enabling deeper assessments of when and why convergence is favored, versus when divergent solutions to CO_2_ concentration can occur. Convergence within C_4_ photosynthesis is apparent in two key steps: the carboxylation of PEP by PEPCase, and the conversion of CO_2_ to bicarbonate by CA. The ubiquitous use of PEPCase is probably due to three factors. First, it is readily available for co-option, because it is widely used in C_3_ plants for processes such as pH control, metabolite generation for the Krebs cycle and nitrogen assimilation, and shuttling reducing power between cellular compartments (Aubry et al., 2011; Mallmann et al., 2014). Second, there are no obvious alternatives in vascular plants that may be co-opted to provide the carboxylation function of a C_4_ cycle. While numerous carboxylases are active in prokaryotes, few occur in higher plants (Erb, 2011). One obvious candidate to recruit into a C_4_ cycle is pyruvate carboxylase, which produces OAA from pyruvate and bicarbonate in bacteria, animals and algae, but it is not known to occur in plants (Tsuji et al., 2012). Third, it is worth considering the chain of events giving rise to C_4_ photosynthesis. The evolutionary rise of C_2_ photosynthesis in C_3_–C_4_ intermediate species establishes a Kranz-like leaf anatomy and M to BS transport systems that facilitate the subsequent upregulation of PEPCase and a C_4_ metabolic cycle (Sage et al., 2012). A leading hypothesis to explain this upregulation is PEPCase provides carbon skeletons to support re-assimilation of photorespiratory ammonia (Mallmann et al., 2014). If so, then the ubiquitous use of PEPCase may arise out of its pre-exiting role supporting N metabolism in C_3_ leaves, which make it the ready candidate for optimizing the performance of C_2_ photosynthesis, which in turn positions it to be further upregulated as the benefits of the nascent C_4_ cycle improve fitness (Heckmann et al., 2013). The convergence upon enhanced CA activity then becomes necessary to enable PEPCase to have enough bicarbonate to maintain rapid activity.

**TABLE 6:** A summary list of convergent and divergent traits of C_4_ photosynthesis.

Convergent trait	Divergent trait
**Biochemical level**	**Biochemical level**
a) PEPCase upregulated in M cytosol only. b) Reduced malate sensitivity of PEPCase. c) Carbonic anhydrase expressed in M tissue only. d) Functional rubisco restricted to high CO_2_ compartment.	a) Variation in decarboxylating enzymes and associated ultrastructural, transport and regulatory traits. b) PEP-CK species may not require PPDK c) Transport metabolites are malate or aspartate
**Structural level**	**Structural level**
a) Interior compartment always used for CO_2_ concentration. b) Reduced M/BS ratio. c) High vein density. d) More plasmodesmata at M x BS boundary.	a) Cell tissue type for CO_2_ concentration (e.g., BS, mestome sheath, inner chloremchyma, central cluster of chloroplasts as in *Bienertia*) b) Variable M and BS arrangement around veins (e.g., partially or incomplete coverage of vein) c) Variable plasmodemata structure (branched, non-branched)
**Ultrastructural level**	**Ultrastructural level**
a) More chloroplast volume in Kranz sheath tissue b) Less chloroplast investment in M cells c) Reduced mitochondria in M cells	a) Chloroplast size, number, shape and position b) Diffusive trap structure (cell wall thickness, suberin presence, vacuole size, chloroplast cluster arrangement) c) Thylakoid stacking and PSII location d) Variable location of whole-chain versus cyclic electron transport e) Mitochondria number, size and position in BS Note: most of the variable traits above become convergent within a given C_4_ subtype.
**Genetic level (PEPCase gene only)**	**Genetic level (PEPCase gene only)**
a) Use of serine at position 780, with some exceptions b) Use of codons in promoter to target M cell expression and expression strength	a) Variation in amino acids at most sites under positive selection b) Variation in PEPCase promoter structure c) Variation in enzyme paralogs co-opted for the C_4_ pathway

*BS, bundle sheath; M, mesophyll; PEP-CK, PEPcase, PEP carboxylase; PEP carboxykinase.*

As the most heavily studied enzyme in C_4_ photosynthesis, the sequence of the PEPCase gene serves as a model of how convergence versus divergence can operate at the gene sequence level. Convergence is apparent in that *PEPC1* was separately co-opted for C_4_ function in the *Allionia* and *Okenia/Boerhavia* lineages, while divergence is apparent in the sequence of *PEPC1* in these clades, as indicated by the distinct branches on the gene trees and variation at specific sites in the amino acid sequences. Is this sequence divergence random, or could it reflect divergent solutions for optimizing PEPcase function in C_4_ leaves? In C_4_ plants, the affinity of PEPCase for its substrate bicarbonate should be increased to compensate for subsaturating bicarbonate concentrations in M tissues (Gowik and Westhoff, 2011; Di Mario and Cousins, 2019). Also, C_4_ orthologs of PEPCase require reduced sensitivity to malate and aspartate, because these allosteric inhibitors of PEPCase must accumulate to high concentration in M cells to drive rapid diffusion into the BS (Jacobs et al., 2008; Gowik and Westhoff, 2011). Numerous studies have documented similar changes in the amino acid sequence of PEPCase in a pattern that is associated with changes in sensitivity to malate, aspartate and PEP, supporting hypotheses of convergent optimization of PEPCase for the C_4_ leaf (Engelmann et al., 2003; Gowik et al., 2006; Christin et al., 2007). Convergence is indicated by a widely noted substitution of a serine for alanine at a homologous site in the PEPC sequence, position 780 in maize, which is proposed to alter PEP and possibly bicarbonate sensitivity (Christin et al., 2007; Gowik and Westhoff, 2011; Di Mario and Cousins, 2019). Christin et al. (2007) also observed common sequence substitutions in numerous distinct clades of C_4_ grasses, some of which occur in gene regions controlling sensitivity to malate. However, transcriptome surveys of numerous chenopods indicate the sequence convergence observed in grasses is less common in eudicots, suggesting divergent solutions to meeting the kinetic and regulatory requirements of a C_4_ PEPCase (Rosnow et al., 2014, 2015). Our results with *Boerhavia* and *Allionia* support a hypothesis of flexibility in how PEPCase is modified for the C_4_ leaf. Both clades lack the serine at the position near 780, instead sharing an alanine with their C_3_ sisters. They also exhibit differences in half of the sequence positions where Christin et al. (2007) noted convergence in grasses, but do exhibit some of the same substitutions as C_4_ grasses and certain C_4_ eudicots (at positions 572, 577, 761, and 807; Table 5). These patterns raise a number of possibilities. On the one hand, these convergent substitutions may sufficiently alter kinetics to allow the C_4_ PEPCase to efficiently function. Alternatively, other sequence shifts may be able to accomplish the necessary change in sensitivities. A third possibility is the C_4_ Nyctaginaceae species may simply have a non-optimized PEPCase for the C_4_ function, in which case other mechanisms may compensate for inefficiencies. One compensation mechanism may be environmental, for example, high temperature in the natural habitat may override a need for convergence in PEPCase sequences by stimulating catalytic capacity. Where convergence is beneficial, but not accomplished, the consequences and compensation mechanisms become as interesting as the convergence itself.

We next consider the evolutionary transition at the opposite end of the C_4_ pathway, where there is clear divergence in decarboxylase function. The divergence arises because there are enzymatic alternatives to meeting the decarboxyation imperative, with NADP-ME, NAD-ME, and PCK having significant roles in numerous metabolic pathways in C_3_ plants (Aubry et al., 2011). Elevated activities of NADP-ME, NAD-ME, and PCK have been detected in vascular tissues and BS cells of C_3_ species, where they metabolize organic acids used in long-distance transport from the roots, and may have a role in pH homeostasis, coordinating carbon and nitrogen metabolism, and providing metabolites for numerous biosynthetic pathways (Hibberd and Quick, 2002; Aubry et al., 2011). The mechanism by which one decarboxylase is selected over another is not known, but since all three are active in BS tissues, it is possible that each may function in a nascent C_4_ pathway, perhaps to support recovery of photorespired nitrogen in the BS (Mallmann et al., 2014). It may be that chance determines which of the three decarboxylases is upregulated as the C_4_ pathway strengthens, with convergence on the distinctive subtype characteristics occurring afterward, as the C_4_ pathway is optimized for the selected decarboxylase. Alternatively, pre-existing traits in the C_3_ or C_2_ ancestors may determine which decarboxylase is selected and also influence the characteristics of each C_4_ subtype.

To close, we note that comparative studies of evolutionary convergence and divergence in C_4_ plants show that convergence is greatest where constraints are high and biochemical options are limited, while divergence occurs where the constraints are low and multiple alternative solutions can be selected during the evolutionary process. What remains unclear is the influence that chance, ancestry, and environment have over divergent possibilities. By demonstrating multiple sets of closely related clades differing in C_4_ subtype, we have identified replicated examples with which to address these issues using comparative methods of evolutionary biology (Harvey and Pagel, 1991). C_4_ photosynthesis can thus become a powerful tool to unravel the intricacies of convergence in complex trait evolution.

## Data Availability Statement

The datasets presented in this study can be found in online repositories. The names of the repository/repositories and accession number(s) can be found in the article/Supplementary Material.

## Author Contributions

RK led the imaging efforts and their quantification and ecological data analysis. MS conducted the phylotranscriptomic and gene sequence analyses. SA performed gas exchange and enzyme assays. TS and RS directed the labs where the work was conducted and supplied funding. RS wrote the manuscript with input from each co-author. All authors contributed to the article and approved the submitted version.

## Dedication

This paper is dedicated to the memory of Dr. Udo Gowik (1971–2020), a fun-loving friend, helpful to all, a pioneer in C_4_ plant biology and a fearsome political debater.

## Conflict of Interest

The authors declare that the research was conducted in the absence of any commercial or financial relationships that could be construed as a potential conflict of interest.

## Footnotes

1 The tissue layer where CO_2_ is concentrated is often comprised of parenchymatous bundle sheath cells; however, in an example of the non-convergence, other cell layers have also been co-opted as the site of CO_2_ concentration. Despite this variation, bundle sheath (BS) is the term generally used to refer to the high CO_2_ compartment. We generally follow this convention here, although we do use the anatomically correct name when discussing a specific Kranz anatomy cell type that is not technically BS tissue.

2 www.isotopes.wsu.edu

3 http://ncbi.nlm.nih.gov/sra

4 http://phytozome.jgi.doe.gov/

5 http://tree.bio.ed.ac.uk/software/figtree

6 www.onekp.com

7 https://www.gbif.org/occurrence/

8 https://www.worldclim.org/

9 https://cgiarcsi.community

10 www.mobot.org/MOBOT/research/APweb

## References

[B1] Aberer A. J. Kobert K. Stamatakis A. (2014). ExaBayes: massively Bayesian tree inference for the whole-genome era. *Mol. Biol. Evol.* 31 2553–2556. 10.1093/molbev/msu236 25135941 PMC4166930

[B2] Alonso-Cantabrana H. von Caemmerer S. (2016). Carbon isotope discrimination as a diagnostic tool for C_4_ photosynthesis in C_3_–C_4_ intermediate species. *J. Exp. Bot.* 67 3109–3121. 10.1093/jxb/erv555 26862154 PMC4867892

[B3] Arnon D. I. (1949). Copper enzymes in isolated chloroplasts: polyphenoloxidase in *Beta vulgaris*. *Plant Physiol.* 24 1–15. 10.1104/pp.24.1.1 16654194 PMC437905

[B4] Ashton A. R. Burnell J. N. Furbank R. T. Jenkins C. L. D. Hatch M. D. (1990). “Enzymes of C_4_ photosynthesis,” in *Methods in Plant Biochemistry*, Vol. 7A ed. Lea P. J., (London: Academic Press), 39–72. 10.1016/B978-0-12-461013-2.50010-1

[B5] Aubry S. Brown N. J. Hibberd J. M. (2011). The role of proteins in C_3_ plants prior to their recruitment into the C_4_ pathway. *J. Exp. Bot.* 62 3049–3059. 10.1093/jxb/err012 21321052

[B6] Bates D. Mächler M. Bolker B. Walker S. (2015). Fitting linear mixed-effects models using lme4. *J. Stat. Softw.* 67 1–48. 10.18637/jss.v067.i01

[B7] Bissinger K. Khoshravesh R. Kotrade J. P. Oakley J. Sage T. L. Sage R. F. (2014). *Gisekia* (Gisekiaceae): phylogenetic relationships, biogeography, and ecophysiology of a poorly known C_4_ lineage in the Caryophyllales. *Am. J. Bot.* 101 499–509. 10.3732/ajb.1300279 24638165

[B8] Bivand R. Lewin-Koh N. (2013). *maptools: Tools for Reading and Handling Spatial Objects. R Package Version 0.8–27.* Available online at: https://cran.r-project.org/web/packages/maptools/index.html (accessed August 24, 2020).

[B9] Blount Z. D. Lenski R. E. Losos J. B. (2018). Contingency and determinism in evolution: replaying life’s tape. *Science* 362:eaam5979. 10.1126/science.aam5979 30409860

[B10] Bohley K. Joos O. Hartmann H. Sage R. F. Liede-Schumann S. Kadereit G. (2015). phylogeny of sesuvioideae (Aizoaceae)-biogeography, leaf anatomy and the evolution of C_4_photosynthesis. *Perspect. Plant Ecol.* 17 116–130.

[B11] Bolger A. M. Lohse M. Usadel B. (2014). Trimmomatic: a flexible trimmer for Illumina sequence data. *Bioinformatics* 30 2114–2120. 10.1093/bioinformatics/btu170 24695404 PMC4103590

[B12] Bräutigam A. Gowik U. (2016). Photorespiration connects C_3_ and C_4_ photosynthesis. *J. Exp. Bot.* 67 2953–2962. 26912798 10.1093/jxb/erw056

[B13] Bräutigam A. Weber A. P. M. (2011). “Transport processes: connecting the reactions of C_4_ photosynthesis,” in *C_4_ Photosynthesis and Related CO_2_ Concentrating Mechanisms*, eds Raghavendra A. S. Sage R. F., (Dordrecht: Springer Netherlands), 277–300.

[B14] Capella-Gutiérrez S. Silla-Martínez J. M. Gabaldón T. (2009). trimAl: a tool for automated alignment trimming in large-scale phylogenetic analyses. *Bioinformatics* 25 1972–1973. 10.1093/bioinformatics/btp348 19505945 PMC2712344

[B15] Carolin R. Jacobs S. Vesk M. (1978). Kranz cells and mesophyll in the Chenopodiales. *Aust. J. Bot.* 26:683 10.1071/BT9780683

[B16] Christin P.-A. Besnard G. Samaritani E. Duvall M. R. Hodkinson T. R. Savolainen V. (2008). Oligocene CO_2_ decline promoted C_4_ photosynthesis in grasses. *Curr. Biol.* 18 37–43. 10.1016/j.cub.2007.11.058 18160293

[B17] Christin P.-A. Boxall S. F. Gregory R. Edwards E. J. Hartwell J. Osborne C. P. (2013a). Parallel recruitment of multiple genes into C_4_ photosynthesis. *Genome Biol. Evol.* 5 2174–2187. 10.1093/gbe/evt168 24179135 PMC3845648

[B18] Christin P.-A. Edwards E. J. Besnard G. Boxall S. F. Gregory R. Kellogg E. A. (2012a). Adaptive evolution of C_4_ photosynthesis through recurrent lateral gene transfer. *Curr. Biol.* 22 445–449. 10.1016/j.cub.2012.01.054 22342748

[B19] Christin P.-A. Freckleton R. P. Osborne C. P. (2010a). Can phylogenetics identify C_4_ origins and reversals? *Trends Ecol. Evol.* 25 403–409. 10.1016/j.tree.2010.04.007 20605250

[B20] Christin P.-A. Osborne C. P. (2013). The recurrent assembly of C_4_ photosynthesis, an evolutionary tale. *Photosynth. Res.* 117 163–175. 10.1007/s11120-013-9852-z 23703454

[B21] Christin P.-A. Osborne C. P. Chatelet D. S. Columbus J. T. Besnard G. Hodkinson T. R. (2013b). Anatomical enablers and the evolution of C_4_ photosynthesis in grasses. *Proc. Natl. Acad. Sci. U.S.A.* 110 1381–1386. 10.1073/pnas.1216777110 23267116 PMC3557070

[B22] Christin P. A. Osborne C. P. Sage R. F. Arakaki M. Edwards E. J. (2011). C_4_ eudicots are not younger than C_4_ monocots. *J. Exp. Bot.* 62 3171–3181. 10.1093/jxb/err041 21393383

[B23] Christin P.-A. Salamin N. Savolainen V. Duvall M. R. Besnard G. (2007). C_4_ photosynthesis evolved in grasses via parallel adaptive genetic changes. *Curr. Biol.* 17 1241–1247. 10.1016/j.cub.2007.06.036 17614282

[B24] Christin P.-A. Samaritani E. Petitpierre B. Salamin N. Besnard G. (2009). Evolutionary insights on C_4_ photosynthetic subtypes in grasses from genomics and phylogenetics. *Genome Biol. Evol.* 1 221–230. 10.1093/gbe/evp020 20333192 PMC2817415

[B25] Christin P.-A. Wallace M. J. Clayton H. Edwards E. J. Furbank R. T. Hattersley P. W. (2012b). Multiple photosynthetic transitions, polyploidy, and lateral gene transfer in the grass subtribe Neurachninae. *J. Exp. Bot.* 63 6297–6308. 10.1093/jxb/ers282 23077201 PMC3481218

[B26] Christin P. A. Weinreich D. M. Besnard G. (2010b). Causes and evolutionary significance of genetic convergence. *Trends Genet.* 26 400–405. 10.1016/j.tig.2010.06.005 20685006

[B27] Conway-Morris S. (2003). *Life’s Solutions: Inevitable Humans in a Lonely Universe.* Cambridge: Cambridge University Press.

[B28] Danila F. R. Quick W. P. White R. G. Kelly S. von Caemmerer S. Furbank R. T. (2018). Multiple mechanisms for enhanced plasmodesmata density in disparate subtypes of C_4_ grasses. *J. Exp. Bot.* 69 1135–1145. 10.1093/jxb/erx456 29300922 PMC6018992

[B29] Dengler N. G. Nelson T. (1999). “Leaf structure and development in C_4_ plants,” in *C_4_ Plant Biology*, eds Sage R. F. Monson R. K., (San Diego: Academic Press), 133–172.

[B30] Di Mario R. J. Cousins A. B. (2019). A single serine to alanine substitution decreases bicarbonate affinity of phosphoenol pyruvate carboxylase in C_4_ *Flaveria trinervia*. *J. Exp. Bot.* 70 995–1004. 10.1093/jxb/ery403 30517744 PMC6363079

[B31] Douglas N. A. Manos P. S. (2007). Molecular phylogeny of Nyctaginaceae: taxonomy, biogeography, and characters associated with a radiation of xerophytic genera in North America. *Am. J. Bot.* 94 856–872. 10.3732/ajb.94.5.856 21636455

[B32] Douglas N. A. Spellenberg R. (2010). A new tribal classification of Nyctaginaceae. *Taxon* 59 905–910. 10.1002/TAX.593018

[B33] Drincovich M. F. Lara M. V. Andreo C. S. Maurino V. G. (2011). “C_4_ decarboxylases: different solutions for the same biochemical problem, the provision of CO_2_ to Rubisco in the bundle sheath cells,” in *C_4_ Photosynthesis and Related CO_2_ Concentrating Mechanisms*, eds Raghavendra A. S. Sage R. F., (Dordrecht: Springer Netherlands), 277–300.

[B34] Dunning L. T. Lundgren M. R. Moreno-Villena J. J. Namaganda M. Edwards E. J. Nosil P. (2017). Introgression and repeated co-option facilitated the recurrent emergence of C_4_ photosynthesis among close relatives: complex transitions among photosynthetic types. *Evolution* 71 1541–1555. 10.1111/evo.13250 28395112 PMC5488178

[B35] Edwards E. J. (2019). Evolutionary trajectories, accessibility and other metaphors: the case of C_4_ and CAM photosynthesis. *New Phytol.* 223 1742–1755. 10.1111/nph.15851 30993711

[B36] Edwards G. E. Voznesenskaya E. V. (2011). “C_4_ photosynthesis: kranz forms and single-cell C_4_ in terrestrial plants,” in *C_4_ Photosynthesis and Related CO_2_ Concentrating Mechanisms*, eds Raghavendra A. S. Sage R. F., (Dordrecht: Springer Netherlands), 29–61.

[B37] Edwards G. E. Walker D. A. (1983). *C_3_, C_4_: Mechanism, and Cellular and Environmental Regulation, of Photosynthesis.* Oxford: Blackwell Scientific Publications.

[B38] Emms D. M. Covshoff S. Hibberd J. M. Kelly S. (2016). Independent and parallel evolution of new genes by gene duplication in two origins of C_4_ photosynthesis provides new insight into the mechanism of phloem loading in C_4_ species. *Mol. Biol. Evol.* 33 1796–1806. 10.1093/molbev/msw057 27016024 PMC4915358

[B39] Emms D. M. Kelly S. (2015). OrthoFinder: solving fundamental biases in whole genome comparisons dramatically improves orthogroup inference accuracy. *Genome Biol.* 16:157. 10.1186/s13059-015-0721-2 26243257 PMC4531804

[B40] Engelmann S. Bläsing O. E. Gowik U. Svensson P. Westhoff P. (2003). Molecular evolution of C_4_ phosphoenolpyruvate carboxylase in the genus *Flaveria*? A gradual increase from C_3_ to C_4_ characteristics. *Planta* 217 717–725. 10.1007/s00425-003-1045-0 12811556

[B41] Erb T. J. (2011). Carboxylases in natural and synthetic microbial pathways. *Appl. Environ. Microbiol.* 77 8466. 10.1128/AEM.05702-11 22003013 PMC3233076

[B42] Farquhar G. D. Ehleringer J. R. Hubick K. T. (1989). Carbon isotope discrimination and photosynthesis. *Annu. Rev. Plant. Physiol. Plant. Mol. Biol.* 40 503–537. 10.1146/annurev.pp.40.060189.002443

[B43] Fick S. E. Hijmans R. J. (2017). Worldclim 2: new 1-km spatial resolution climate surfaces for global land areas. *Int. J. Climatol.* 37 4302–4315. 10.1002/joc.5086

[B44] Furbank R. T. (2011). Evolution of the C_4_ photosynthetic mechanism: are there really three C_4_ acid decarboxylation types? *J. Exp. Bot.* 62 3103–3108. 10.1093/jxb/err080 21511901

[B45] Ghannoum O. Evans J. R. Caemmerer S. (2011). “Nitrogen and water use efficiency of C_4_ plants”,” in *C_4_ Photosynthesis and Related CO_2_ Concentrating Mechanisms*, eds Raghavendra A. S. Sage R. F., (Dordrecht: The Netherlands: Springer), 129–146.

[B46] Gowik U. Bräutigam A. Weber K. L. Weber A. P. M. Westhoff P. (2011). Evolution of C_4_ photosynthesis in the genus *Flaveria*: how many and which genes does it take to make C_4_? *Plant Cell* 23 2087–2105. 10.1105/tpc.111.086264 21705644 PMC3160039

[B47] Gowik U. Engelmann S. Bläsing O. E. Raghavendra A. S. Westoff P. (2006). Evolution of C_4_ phosphoenolpyruvate carboxylase in the genus *Alternanthera:* gene families and the enzymatic characterisitics of the C_4_ isozyme and its orthologues in C_3_ and C_4_ *Alternanthera*. *Planta* 223 359–368. 10.1007/s00425-005-0085-z 16136331

[B48] Gowik U. Westhoff P. (2011). “C_4_-phosphoenolpyruvate carboxylase,” in *C_4_ Photosynthesis and Related CO_2_ Concentrating Mechanisms*, eds Raghavendra A. S. Sage R. F., (Dordrecht: Springer Netherlands), 257–275.

[B49] Grabherr M. G. Haas B. J. Yassour M. Levin J. Z. Thompson D. A. Amit I. (2011). Trinity: reconstructing a full-length transcriptome without a genome from RNA-Seq data. *Nat. Biotechnol.* 29 644–652. 10.1038/nbt.1883 21572440 PMC3571712

[B50] Griffiths H. Weller G. Toy L. F. M. Dennis R. J. (2013). You’re so vein: bundle sheath physiology, phylogeny and evolution in C_3_ and C_4_ plants: origins and function of bundle sheath in C_3_ plants. *Plant Cell Environ.* 36 249–261. 10.1111/j.1365-3040.2012.02585 22827921

[B51] Gutierrez M. Gracen V. E. Edwards G. E. (1974). Biochemical and cytological relationships in C_4_ plants. *Planta* 119 279–300. 10.1007/BF00388331 24442564

[B52] Harvey P. H. Pagel M. D. (1991). *The Comparative Method in Evolutionary Biology.* Oxford: Oxford Press.

[B53] Hatch M. Kagawa T. Craig S. (1975). Subdivision of C_4_-pathway species based on differing C_4_ acid decarboxylating systems and ultrastructural features. *Funct. Plant Biol.* 2:111 10.1071/PP9750111

[B54] Hatch M. D. (1987). C_4_ photosynthesis: a unique blend of modified biochemistry, anatomy and ultrastructure. *Biochim. Biophys. Acta Rev. Bioenerget.* 895 81–106. 10.1016/S0304-4173(87)80009-5

[B55] Hattersley P. W. (1992). “C_4_ photosynthetic pathway variation in grasses (Poaceae): its significance for arid and semi-arid lands,” in *Desertified Grasslands: their Biology and Management*, ed. Chapman G. P., (London: Academic Press), 181–212.

[B56] Heckmann D. Schulze S. Denton A. Gowik U. Westhoff P. Weber A. P. M. (2013). Predicting C_4_ photosynthesis evolution: modular, individually adaptive steps on a Mount Fuji fitness landscape. *Cell* 153 1579–1588. 10.1016/j.cell.2013.04.058 23791184

[B57] Heyduk K. Moreno-Villena J. J. Gilman I. S. Christin P.-A. Edwards E. J. (2019). The genetics of convergent evolution: insights from plant photosynthesis. *Nat. Rev. Genet.* 20 485–493. 10.1038/s41576-019-0107-5 30886351

[B58] Hibberd J. M. Quick W. P. (2002). Characteristics of C_4_ photosynthesis in stems and petioles of C_3_ flowering plants. *Nature* 415 451–454. 11807559 10.1038/415451a

[B59] Hijmans R. J. van Etten J. (2012). *raster: Geographic Analysis and Modeling with Raster Data. R package Version 2.0-12.* Available online at: http://CRAN.R-project.org/package=raster (accessed July 17, 2020).

[B60] Huelsenbeck J. P. Ronquist F. (2001). MRBAYES: bayesian inference of phylogenetic trees. *Bioinformatics* 17 754–755. 10.1093/bioinformatics/17.8.754 11524383

[B61] Jacobs B. Engelmann S. Westhoff P. Gowik U. (2008). Evolution of C_4_ phosphoenolpyruvate carboxylase in *Flaveria*: determinants for high tolerance towards the inhibitor L-malate. *Plant Cell Environ.* 31 793–803. 10.1111/j.1365-3040.2008.01796 18266899

[B62] John C. R. Smith-Unna R. D. Woodfield H. Covshoff S. Hibberd J. M. (2014). Evolutionary convergence of cell-specific gene expression in independent lineages of C_4_ grasses. *Plant Physiol.* 165 62–75. 10.1104/pp.114.238667 24676859 PMC4012605

[B63] Kadereit G. Ackerly D. Pirie M. D. (2012). A broader model for C_4_ photosynthesis evolution in plants inferred from the goosefoot family (Chenopodiaceae s.s.). *Proc. R. Soc. B.* 279 3304–3311. 10.1098/rspb.2012.0440 PMC3385724 22628474

[B64] Kadereit G. Lauterbach M. Pirie M. D. Arafeh R. Freitag H. (2014). When do different C_4_ leaf anatomies indicate independent C_4_ origins? Parallel evolution of C_4_ leaf types in Camphorosmeae (Chenopodiaceae). *J. Exp. Bot.* 65 3499–3511. 10.1093/jxb/eru169 24811953

[B65] Kanai R. Edwards G. (1999). “The biochemistry of C_4_ photosynthesis,” in *C_4_ Plant Biology*, eds Sage R. F. Monson R. K., (San Diego, CA: Academic Press), 49–87.

[B66] Katoh K. Standley D. M. (2013). MAFFT Multiple Sequence Alignment Software Version 7: improvements in performance and usability. *Mol. Biol. Evol.* 30 772–780. 10.1093/molbev/mst010 23329690 PMC3603318

[B67] Khoshravesh R. Akhani H. Sage T. L. Nordenstam B. Sage R. F. (2012). Phylogeny and photosynthetic pathway distribution in *Anticharis* Endl. (Scrophulariaceae). *J. Exp. Bot.* 63 5645–5658. 10.1093/jxb/ers218 22945938

[B68] Khoshravesh R. Lundsgaard-Nielsen V. Sultmanis S. Sage T. L. (2017). “Light microscopy, transmission electron microscopy, and immunohistochemistry protocols for studying photorespiration,” in *Photorespiration: Methods and Protocols*, eds Fernie A. R. Bauwe H. Weber A. P. M., (New York, NY: Springer), 243–270. 10.1007/978-1-4939-7225-8_17 28822138

[B69] Khoshravesh R. Stata M. Busch F. A. Saladié M. Castelli J. M. Dakin N. (2020). The evolutionary origin of C_4_ photosynthesis in the grass subtribe Neurachninae. *Plant Physiol.* 182 566–583. 10.1104/pp.19.00925 31611421 PMC6945869

[B70] Khoshravesh R. Stinson C. R. Stata M. Busch F. A. Sage R. F. Ludwig M. (2016). C_3_–C_4_ intermediacy in grasses: organelle enrichment and distribution, glycine decarboxylase expression, and the rise of C_2_ photosynthesis. *J. Exp. Bot.* 67 3065–3078. 10.1093/jxb/erw150 27073202 PMC4867898

[B71] Kim D. Paggi J. M. Park C. Bennett C. Salzberg S. L. (2019). Graph-based genome alignment and genotyping with HISAT2 and HISAT-genotype. *Nat. Biotechnol.* 37 907–915. 10.1038/s41587-019-0201-4 31375807 PMC7605509

[B72] Kim I. Fisher D. G. (1990). Structural aspects of the leaves of seven species of *Portulaca* growing in Hawaii. *Can. J. Bot.* 68 1803–1811. 10.1139/b90-233

[B73] Lauterbach M. Zimmer R. Alexa A. C. Adachi S. Sage R. Sage T. (2019). Variation in leaf anatomical traits relates to the evolution of C_4_ photosynthesis in Tribuloideae (Zygophyllaceae). *Perspect. Plant Ecol.* 39:125463 10.1016/j.ppees.2019.125463

[B74] Lê S. Josse J. Husson F. (2008). FactoMineR: a package for multivariate analysis. *J. Stat. Softw.* 25 1–18. 10.18637/jss.v025.i01

[B75] Leegood R. C. Walker R. O. (1999). “Regulation of the C_4_ pathway,” in *C_4_ Plant Biology*, eds Sage R. F. Monson R. K., (San Diego, CA: Academic Press), 89–131.

[B76] Losos J. B. (2011). Convergence, adaptation, and constraint. *Evolution* 65 1827–1840. 10.1111/j.1558-5646.2011.01289 21729041

[B77] Ludwig M. (2016a). Evolution of carbonic anhydrases in C_4_ plants. *Curr. Opin. Plant Sci.* 31 16–22. 10.1016/j.pbi.2016.03.003 27016649

[B78] Ludwig M. (2016b). The roles of organic acids in C_4_ photosynthesis. *Front. Plant Sci.* 7:647. 10.3389/fpls.2016.00647 27242848 PMC4868847

[B79] Mallmann J. Heckmann D. Bräutigam A. Lercher M. J. Weber A. P. Westhoff P. (2014). The role of photorespiration during the evolution of C_4_ photosynthesis in the genus *Flaveria*. *eLife* 3:e02478. 10.7554/eLife.02478 24935935 PMC4103682

[B80] Monson R. K. Teeri J. A. Ku M. S. B. Gurevitch J. Mets L. J. Dudley S. (1988). Carbon-isotope discrimination by leaves of *Flaveria* species exhibiting different amounts of C_3_–and C_4_-cycle co-function. *Planta* 174 145–151. 10.1007/BF00394765 24221469

[B81] Moore B. D. Ku M. S. B. Edwards G. E. (1989). Expression of C_4_-like photosynthesis in several species of *Flaveria*. *Plant Cell Environ.* 12 541–549. 10.1111/j.1365-3040.1989.tb02127

[B82] Muhaidat R. Sage R. F. Dengler N. G. (2007). Diversity of Kranz anatomy and biochemistry in C_4_ eudicots. *Am. J. Bot.* 94 362–381. 10.3732/ajb.94.3.362 21636407

[B83] Muhaidat R. Sage T. L. Frohlich M. W. Dengler N. G. Sage R. F. (2011). Characterization of C_3_–C_4_ intermediate species in the genus *Heliotropium* L. (Boraginaceae): anatomy, ultrastructure and enzyme activity. *Plant Cell Environ.* 34 1723–1736. 10.1111/j.1365-3040.2011.02367 21631534

[B84] Oakley J. C. Sultmanis S. Stinson C. R. Sage T. L. Sage R. F. (2014). Comparative studies of C_3_ and C_4_ *Atriplex* hybrids in the genomics era: physiological assessments. *J. Exp. Bot.* 65 3637–3647. 10.1093/jxb/eru106 24675672 PMC4085961

[B85] Ocampo G. Koteyeva N. K. Voznesenskaya E. V. Edwards G. E. Sage T. L. Sage R. F. (2013). Evolution of leaf anatomy and photosynthetic pathways in Portulacaceae. *Am. J. Bot.* 100 2388–2402. 10.3732/ajb.1300094 24259525

[B86] Price M. N. Dehal P. S. Arkin A. P. (2010). FastTree 2–approximately maximum-likelihood trees for large alignments. *PLoS One* 5:e9490. 10.1371/journal.pone.0009490 20224823 PMC2835736

[B87] Pyankov V. I. Voznesenskaya E. V. Kuz’min A. N. Ku M. S. B. Ganko E. Franceschi V. R. (2000). Occurrence of C_3_ and C_4_ photosynthesis in cotyledons and leaves of *Salsola* species (Chenopodiaceae). *Photosynth. Res.* 63 69–84. 10.1023/A:1006377708156 16252166

[B88] Rao X. Dixon R. A. (2016). The differences between NAD-ME and NADP-ME subtypes of C_4_ photosynthesis: more than decarboxylating enzymes. *Front. Plant Sci.* 7:1525. 10.3389/fpls.2016.01525 27790235 PMC5061750

[B89] Revell L. J. (2012). phytools: an R package for phylogenetic comparative biology (and other things).” *Methods Ecol. Evol.* 3 217–223. 10.1111/j.2041-210X.2011.00169

[B90] Reyna-Llorens I. Hibberd J. M. (2017). Recruitment of pre-existing networks during the evolution of C_4_ photosynthesis. *Phil. Trans. R. Soc. B* 372:20160386. 10.1098/rstb.2016.0386 28808102 PMC5566883

[B91] Rice P. Longden I. Bleasby A. (2000). EMBOSS: the European molecular biology open software suite. *Trends Genet.* 16 276–277. 10827456 10.1016/s0168-9525(00)02024-2

[B92] Rosnow J. J. Edwards G. E. Roalson E. H. (2014). Positive selection of Kranz and non-Kranz C_4_ phosphoenolpyruvate carboxylase amino acids in Suaedoideae (Chenopodiaceae). *J. Exp. Bot.* 65 3595–3607. 10.1093/jxb/eru053 24600021 PMC4085955

[B93] Rosnow J. J. Evans M. A. Kapralov M. V. Cousins A. B. Edwards G. E. Roalson E. H. (2015). Kranz and single-cell forms of C_4_ plants in the subfamily Suaedoideae show kinetic C_4_ convergence for PEPC and Rubisco with divergent amino acid substitutions. *J. Exp. Bot.* 66 7347–7358. 10.1093/jxb/erv431 26417023 PMC4765798

[B94] Sage R. F. (2004). The evolution of C_4_ photosynthesis. *New Phytol.* 161 341–370. 10.1111/j.1469-8137.2004.00974 33873498

[B95] Sage R. F. (2016). A portrait of the C_4_ photosynthetic family on the 50th anniversary of its discovery: species number, evolutionary lineages, and Hall of Fame. *J. Exp. Bot.* 67 4039–4056. 10.1093/jxb/erw156 27053721

[B96] Sage R. F. Christin P.-A. Edwards E. J. (2011). The C_4_ plant lineages of planet Earth. *J. Exp. Bot.* 62 3155–3169. 10.1093/jxb/err048 21414957

[B97] Sage R. F. Khoshravesh R. Sage T. L. (2014). From proto-Kranz to C_4_ Kranz: building the bridge to C_4_ photosynthesis. *J. Exp. Bot.* 65 3341–3356. 10.1093/jxb/eru180 24803502

[B98] Sage R. F. Monson R. K. Ehleringer J. R. Adachi S. Pearcy R. W. (2018). Some like it hot: the physiological ecology of C_4_ plant evolution. *Oecologia* 187 941–966. 10.1007/s00442-018-4191-6 29955992

[B99] Sage R. F. Sage T. L. Kocacinar F. (2012). Photorespiration and the evolution of C_4_ photosynthesis. *Annu. Rev. Plant Biol.* 63 19–47. 10.1146/annurev-arplant-042811-105511 22404472

[B100] Sage T. L. Busch F. A. Johnson D. C. Friesen P. C. Stinson C. R. Stata M. (2013). Initial events during the evolution of C_4_ photosynthesis in C_3_ species of *Flaveria*. *Plant Physiol.* 163 1266–1276. 10.1104/pp.113.221119 24064930 PMC3813649

[B101] Sage T. L. Sage R. F. Vogan P. J. Rahman B. Johnson D. C. Oakley J. C. (2011). The occurrence of C_2_ photosynthesis in *Euphorbia* subgenus *Chamaesyce* (Euphorbiaceae). *J. Exp. Bot.* 62 3183–3195. 10.1093/jxb/err059 21459765

[B102] Schneider C. A. Rasband W. S. Eliceiri K. W. (2012). NIH Image to ImageJ: 25 years of image analysis. *Nat. Methods* 9 671–675. 10.1038/nmeth.2089 22930834 PMC5554542

[B103] Schulze S. Mallmann J. Burscheidt J. Koczor M. Streubel M. Bauwe H. (2013). Evolution of C_4_ photosynthesis in the genus *Flaveria*: establishment of a photorespiratory CO_2_ pump. *Plant Cell* 25 2522–2535. 10.1105/tpc.113.114520 23847152 PMC3753380

[B104] Spellenberg R. W. (2003). Nyctaginaceae. *Flora North Am.* 4 14–74.

[B105] Stamatakis A. (2014). RAxML version 8: a tool for phylogenetic analysis and post-analysis of large phylogenies. *Bioinformatics* 30 1312–1313. 24451623 10.1093/bioinformatics/btu033 PMC3998144

[B106] Stata M. Sage T. L. Rennie T. D. Khoshravesh R. Sultmanis S. Khaikin Y. (2014). Mesophyll cells of C_4_ plants have fewer chloroplasts than those of closely related C_3_ plants: C_3_ versus C_4_ mesophyll chloroplasts. *Plant Cell Environ.* 37 2587–2600. 10.1111/pce.12331 24689501

[B107] Suyama M. Torrents D. Bork P. (2006). PAL2NAL: robust conversion of protein sequence alignments into the corresponding codon alignments. *Nucleic Acids Res.* 34 W609–W612. 10.1093/nar/gkl315 16845082 PMC1538804

[B108] Taub D. R. (2000). Climate and the U.S. distribution of C_4_ grass subfamilies and decarboxylation variants of C_4_ photosynthesis. *Am. J. Bot.* 87 1211–1215. 10.2307/2656659 10948007

[B109] Thimm O. Bläsing O. Gibon Y. Nagel A. Meyer S. Krüger P. (2004). MAPMAN: a user-driven tool to display genomics data sets onto diagrams of metabolic pathways and other biological processes. *Plant J.* 37 914–939. 10.1111/j.1365-313x.2004.02016.x 14996223

[B110] Tropicos (2020). *Tropicos.org. Missouri Botanical Garden* http://www.tropicos.org (accessed October 20, 2020).

[B111] Tsuji Y. Suzuki I. Shiraiwa Y. (2012). Enzymological evidence for the function of a plastid-located pyruvate carboxylase in the haptophyte alga *Emiliania huxleyi*: a novel pathway for the production of C_4_ compounds. *Plant Cell Physiol.* 53 1043–1052. 10.1093/pcp/pcs045 22492231

[B112] Ueno O. (1996). Structural characterization of photosynthetic cells in an amphibious sedge, *Eleocharis vivipara*, in relation to C_3_ and C_4_ metabolism. *Planta* 199 382–393. 10.1007/BF00195730

[B113] Ueno O. (2013). Ultrastructure and carbon isotope ratios of leaves in C_4_ species of *Rhynchospora* (Cyperaceae) that differ in the location of Kranz cells. *Int. J. Plant Sci.* 174 702–709. 10.1086/669912

[B114] Vogel J. C. Fuls A. Ellis R. P. (1978). The geographical distribution of Kranz grasses in South Africa. *S. Afr. J. Sci.* 74 209–215.

[B115] Von Caemmerer S. (1992). Carbon isotope discrimination in C_3_−C_4_ intermediates. *Plant Cell Environ.* 15 1063–1072. 10.1111/j.1365-3040.1992.tb01656

[B116] von Caemmerer S. Furbank R. T. (2003). The C_4_ pathway: an efficient CO_2_ pump. *Photosynth. Res.* 77 191–207. 10.1023/A:1025830019591 16228376

[B117] Voznesenskaya E. V. Akhani H. Koteyeva N. K. Chuong S. D. X. Roalson E. H. Kiirats O. (2008). Structural, biochemical, and physiological characterization of photosynthesis in two C_4_ subspecies of *Tecticornia indica* and the C_3_ species *Tecticornia pergranulata* (Chenopodiaceae). *J. Exp. Bot.* 59 1715–1734. 10.1093/jxb/ern028 18390850

[B118] Voznesenskaya E. V. Chuong S. D. X. Koteyeva N. K. Franceschi V. R. Freitag H. Edwards G. E. (2007). Structural, biochemical, and physiological characterization of C_4_ photosynthesis in species having two vastly different types of Kranz anatomy in genus *Suaeda* (Chenopodiaceae). *Plant Biol.* 9 745–757. 10.1055/s-2007-965579 17891703

[B119] Voznesenskaya E. V. Franceschi V. R. Chuong S. D. X. Edwards G. E. (2006). Functional characterization of phosphoenolpyruvate carboxykinase-type C_4_ leaf anatomy: immuno-, cytochemical and ultrastructural analyses. *Ann. Bot.* 98 77–91. 10.1093/aob/mcl096 16704997 PMC2803547

[B120] Voznesenskaya E. V. Franceschi V. R. Kiirats O. Artyusheva E. G. Freitag H. Edwards G. E. (2002). Proof of C_4_ photosynthesis without Kranz anatomy in *Bienertia cycloptera* (Chenopodiaceae). *Plant J.* 31 649–662. 10.1046/j.1365-313X.2002.01385 12207654

[B121] Voznesenskaya E. V. Franceschi V. R. Pyankov V. I. Edwards G. E. (1999). Anatomy, chloroplast structure and compartmentation of enzymes relative to photosynthetic mechanisms in leaves and cotyledons of species in the tribe Salsoleae (Chenopodiaceae). *J. Exp. Bot.* 50 1779–1795. 10.1093/jxb/50.341.1779 12432039

[B122] Voznesenskaya E. V. Koteyeva N. K. Cousins A. Edwards G. E. (2018). Diversity in structure and forms of carbon assimilation in photosynthetic organs in *Cleome* (Cleomeaceae). *Funct. Plant Biol.* 45:983. 10.1071/FP17323 32290998

[B123] Voznesenskaya E. V. Koteyeva N. K. Edwards G. E. Ocampo G. (2010). Revealing diversity in structural and biochemical forms of C_4_ photosynthesis and a C_3_–C_4_ intermediate in genus *Portulaca* L. (Portulacaceae). *J. Exp. Bot.* 61 3647–3662. 10.1093/jxb/erq178 20591900 PMC2921202

[B124] Yoshimura Y. Kubota F. Ueno O. (2004). Structural and biochemical bases of photorespiration in C_4_ plants: quantification of organelles and glycine decarboxylase. *Planta* 220 307–317. 10.1007/s00425-004-1335-1 15290293

