# Supplementary material for: Evolutionary Convergence of C4 Photosynthesis: A Case Study in the Nyctaginaceae
Source: Front Plant Sci. 2020 Nov 2;11:578739. doi: 10.3389/fpls.2020.578739 (PMC7667235; doi:10.3389/fpls.2020.578739)
Supplement: Supplementary file 1 [file Data_Sheet_1.PDF]

Supplemental Table S1: Source location for live plant materials sampled this study. GPS coordinates are listed with site location.

Species	Location	Collection Date	Collector	Use
Allionia incarnata	Santa Cruz, Arizona, Near St Catherine mission, in dry creekbed 33.226146, -112.172050	August 27, 2016	Rowan Sage, Tammy Sage	Ultrastructure study, gas exchange, enzyme assay
Allionia incarnata	Edge of Highway 170 at Terlingua Springs road, Texas. 29.325, -103.546	~August 22, 2007	Rowan Sage	1KP transcriptome
Anulocaulis gypsogenus	Gypsum badlands along Texas highway 1165 0.5 km N of junction with highway 652. 31.923674, -104.418766	September 6, 2010	Rowan Sage, Florian Busch	Ultrastructure study, 1KP transcriptome
Boerhavia coccinea	Roadside weed along highway 3078 about 1 km west of Balmorhea State Park, at Toyah creek bridge embankment. 30.937575, -103.810161	September 6, 2010	Rowan Sage, Florian Busch	Ultrastructure study, gas exchange, enzyme assay
Boerhavia coccinea	Roadside weed, Old Navaho bridge parking lot, Highway 89a, Marble Canyon, Arizona 31.923674, -104.418766	August 14, 2002	Rowan Sage, Kate Sage	1KP transcriptome
Boerhavia burbigeana (listed as *B. dominii* in Muhaidat et al. 2007)	Sidewalk weed, Darwin, Australia. -12.456517, 103.841141	September 2, 2001	Rowan Sage, Dave Kubien, Jiri Santrucek	1KP transcriptome, TEM ultrastructure
Commicarpus scandens	Dry wash of Rancherias canyon, N of highway 170, 10 miles east of Redford, Texas 29.334893, -104.039924	September 7, 2010	Rowan Sage, Florian Busch	Ultrastructure study, gas exchange, enzyme assay, 1KP transcriptome
Nyctaginia capitata	Roadside weed four miles south of Marathon, Texas along Highway 385. 30.148402, -103.234787	Sept 8, 2010	Rowan Sage, Florian Busch	Ultrastructure study, gas exchange, enzyme assay, 1KP transcriptome

Supplemental Table S2: $\delta^{13}C$ data for sampled species from the Nyctaginaceae. Species listed are based on accepted names in Tropicos (2018), the International Place Names Index (2018), the Flora of North America (Spellenberg 2003), or Flora of Australia (Meikle and Hewson, 1984). Values are means, with sample size in parentheses. C_4 species are highlighted in bold. See online supplemental Table S3 for the herbarium specimen and collection information, and $\delta^{13}C$ values of each sample. [1]*Belemia fucsiodes* was a type specimen and could only be examined with a dissecting scope for vein density.

Abronia

1. *alpina* Brandegee	-25.2 (2)
2. *ameliae* Lundell	-27.6 (2)
3. *ammophila* Greene	-26.2 (2)
4. *angustifolia* Green	-27.0 (2)
5. *bigelovii* Heim.	-24.5 (2)
6. *carletonii* Coult. & Fisher	-27.3 (2)
7. *elliptica* Nelson:	-25.7 (2)
8. *fragrens* Nutt. ex Hook.	-28.1 (2)
9. *grabrifolia* Standl.	-26.0 (2)
10. *latifolia* Eschsch	-25.1 (2)
11. *macrocarpa* Galloway	-29.5 (2)
12. *maritima* Nutt.	-27.3 (1)
13. *mellifera* Douglas.	-26.2 (2)
14. *nana* S. Wats.	-26.7 (2)
15. *nealleyi* Standl.	-27.4 (1)
16. *orbiculata* Standl.	-26.2 (1)
17. *pogonantha* Heim.	-26.4 (2)
18. *turbinata* Torr.	-24.9 (2)
19. *umbellata* Lam.	-26.9 (1)
20. *villosa* Wats.	-24.9 (3)

Allionia

1. ***choisyi*** Standl.	-13.3 (8)
2. ***incarnata*** L.	-13.2 (8)

Andradea floribunda Allemão	-26.0 (2)

Anulocaulis

1. *annulatus* Standl.	-24.2 (1)
2. *eriosolenus* Standl.	-25.4 (5)
3. *gypsogenus* Waterfall	-26.2 (2)
4. *leiosolenus* Standl.	-24.9 (5)
5. *reflexus* Johnston	-28.0 (2)

Acleisanthes

1. *acutifolia* Standl.	-27.0 (1)
2. *angustifolia* R.A. Levin	-23.9 (2)
3. *anisophylla* Gray	-25.8 (1)
4. *chenopodoides* R.A. Levin	-25.3(3)
5. *crassifolia* Gray	-28.0 (1)
6. *diffusa* R.A. Levin	-24.5 (1)
7. *lanceolata* R.A. Levin	-25.5 (2)
8. *longiflora* A. Gray	-25.4 (4)
9. *nevadensis* Turner	-24.0 (2)
10. *obtusa* Standl.	-25.2 (3)
11. *parviflora* R.A. Levin	-25.5 (1)
12. *wrightii* Benth. & Hook	-25.6 (3)
13. *somalensis* Choiv.	-25.4 (1)
14. *undulatus* Fowler & Turner	-26.1 (1)

Belemia fucsiodes Pires	non-Kranz anatomy[1]

Boerhavia

1. ***acutifolia*** J. Moore	-13.7 (1)
2. ***alata*** Watson	-14.3 (3)
3. ***albiflora*** Fosb.	-12.6 (2)
4. ***anisophylla*** Torr.	-13.5 (5)
5. ***ciliata*** Brandegee	-13.9 (1)
6. ***coccinea*** Mill.	-14.3 (4)
7. ***cordobensis*** Kuntze	-13.1 (6)
8. ***coulteri*** Watson	-14.9 (5)
9. ***crispifolia*** Fosb.	-12.7 (4)
10. ***deserticola*** Codd.	-13.1 (4)
11. ***diandra*** L.	-13.6 (2)
12. ***diffusa*** L.	-13.3 (7)
13. ***dominii*** Meikle & Hewson	-13.4 (4)
14. ***elagans*** Choisy	-13.0 (5)
15. ***erecta*** L.	-13.8 (6)
16. ***glabrata*** Blume	-12.8 (2)
17. ***gracillima*** Heim.	-13.4 (4)
18. ***grahamii*** Gray	-12.0 (1)
19. ***herbstii*** Fosb.	-12.8 (1)
20. ***heroensis*** Heim.	-13.2 (4)
21. ***intermedia*** Jones	-13.1 (2)
22. ***lateriflora*** Standl.	-14.4 (2)

23. ***linearifolia*** Gray: -13.3 (6)
24. ***maculata*** Standl. -12.1 (1)
25. ***pterocarpa*** S. Wats. -13.0 (5)
26. ***pulchella*** Grisebach -11.6 (2)
27. ***purpurescens*** Gray -13.1 (4)
28. ***repens*** L. -12.9 (5)
29. ***scabrida*** Steudel -13.0 (1)
30. ***schomburgkiana*** Oliv. -13.8 (2)
31. ***spicata*** Choisy. -13.2 (8)
32. ***tetrandra*** Forst -12.1 (4)
33. ***tomentosa*** Ehrenb. - 9.4 (1)
34. ***torreyana*** Standl. -13.3 (5)
35. ***traubae*** Spellenberg -12.3 (2)
36. ***triquetra*** S. Wats. -12.7 (1)
37. ***verbenacea*** Killip. -12.8 (3)
38. ***verticillata*** Poir. -13.5 (2)
39. ***viscosa*** Laq. & Rodr -12.3 (7)
40. ***vulvarifolia*** Poir. -11.7 (1)
41. ***weberbaueri*** Heim. -12.9 (2)
42. ***wrightii*** Gray -13.0 (4)
43. ***xantii*** S. Wats -13.6 (7)

Cephalotomandra fragrans K. & T. -28.8 (1)

Colignonia
1. *glomerata* Griseb. -28.7 (2)
2. *ovalifolia* Heim. -28.2 (2)
3. *parviflora* Choisy: -25.3 (4)
4. *pentoptera* Bohlin -27.0 (2)
5. *rufopilosa* Kuntze -28.4 (2)
6. *scandens* Benth. -26.0 (2)

Commicarpus
1. *ambiguous* Meilke -26.6 (1)
2. *arabicus* Meikle -26.9 (3)
3. australis Meilke -27.1 (2)
4. boissieri Cufod. -28.1 (3)
5. *chinensis* Heim. -27.9 (3)
6. *decipiens* Meilke -25.4 (1)
7. *fallacissimus* Heim. -25.4 (3)
*8. grandifloras S*tandl. -27.1 (3)
9. greenwayi Meilke -27.1 (2)
10. *heimerlii* Meilke. -26.6 (1)
11. *helenae* Meilke -26.6 (4)
12. *pedunculosus* Cufod. -26.2 (5)
13. *pentandrus* Heim. -26.8 (5)
14. *pilosus* Meilke -27.1 (2)
15. plumbagineus Standl. -27.0 (7)
16. raynalii Lebrun -26.1 (1)
17. *reniformis* Cufod. -28.3 (2)
18. *scandens* Standl. -26.8 (8)
19. *simony*i Meikle -26.5 (3)
20. *sinuatus* Meikle -27.2 (4)
21. *squarrosus* Standl. -25.2 (3)
22. *stenocarpus* Cufod. -28.7 (2)
23. *tuberosus* Standl. -28.5 (5)

Cryptocarpus pyriformis Kunthe. -24.1 (3)

Cyphomeris
1. *crassifolia* Standl. -28.1 (5)
2. gypsophiloides Standl. -27.2 (7)

Grajalesia fasiculata Miranda -25.7 (1)

Leucaster caniflorus Choisy -28.8 (1)

Mirabilis
1. *aggregata* Cav. -28.5 (2)
2. *albida* Heim. -29.8 (2)
3. *alipes* Pilz. -26.3 (2)
4. *austrotexana* Turner -26.8 (2)
5. *coccinea* Benth. & Hook. -26.6 (3)
6. *decipiens* Standl. -27.2 (2)
7. *glabra* Standl. -29.3 (1)
8. *greenei* S. Wats. -28.6 (2)
9. *jalapa* L -28.2 (4)
10. *laevis* Curran. -25.7 (9)
11. *linearis* Heim. -28.6 (2)
12. *longiflora* L. -27.8 (2)
13. *Macfarlanei* -25.5 (2)
14. *melanotricha* Spellenb. -28.5 (2)
15. *multiflora* Gray -24.4 (2)
16. *nyctaginia* MacMill. -29.5 (2)
17. *oligantha* Macbr. -24.0 (1)
18. *oxybaphoides* Gray -27.4 (1)
19. *polyphylla* Standl. -22.6 (1)
20. *sanguinosa* Heim. -27.5 (1)
21. *tenuiloba* Wats. -25.0 (2)
22. *triflora* Benth. -28.6 (1)
23. *wrightiana* Gray -27.5 (1)

Nyctaginia capitata Choisy -26.2 (8)

Okenia

1. ***grandiflora*** Standl.	-12.9 (2)
2. ***hypogaea*** Schltdl. & Cham.	-12.7 (8)
3. ***parviflora*** Wilson	-12.3 (4)
4. **rosei** Standl.	-14.0 (1)

Note: Spellenberg (2003) lists only one species in *Okenia, O. hypogea*.

Phaeoptilum spinosum Radlk.	-24.4 (5)
Pisoniella arborescens Standl.	-25.8 (4)
Ramisia brasiliensis Oliv.	-24.3 (1)
Reichenbachia hirsuta Spreng	-28.7 (2)

Salpianthus

1. *arenarius*	-28.8 (2)
2. *macrodontus* Standl.	-27.4 (1)
3. *purpurescens* Hook & Arn.	-28.3 (2)

Selinocarpus (=*Acleisanthes*, Levin 2000, 2002).

Tripterocalyx

1. *carneus* Galloway	-26.4 (2)
2. *crux-maltae* Standl.	-27.8 (3)
3. *cycloptera* Standl.	-25.0 (1)
4. *micranthus* Hook.	-24.9 (3)

References:

Meikle, .R.D. and Hewson, H.J. (1984) Nyctaginaceae. *Flora of Australia* 4, 5-18.

Spellenberg, R.W., (2003). Nyctaginaceae. *Flora of North America*, 4, 14-74.

Supplementary Table S3: Herbarium specimens of Nyctaginaceae species sampled for $\delta^{13}C$ analysis. Species listed are based on accepted names in Tropicos, the International Place Names Index, the Flora of North America, or Flora of Australia. Where accepted names are uncertain, the species and synonym are listed. Letters in parenthesis indicate the isotope value for the specimen, followed by the herbarium housing the specimen where (K) is the herbarium at Kew gardens, (MO) is the herbarium of the Missouri Botanical Garden, and (NY) is the herbarium of the New York Botanical Garden. Two isotope values are given when two distinct plants on the same herbarium sheet were assayed. Abberviations: AUS, Australia; Island, Isl; MX, Mexico. Excluded species refer to species names that are no longer recognized but for which we have $\delta^{13}C$ data.

Abronia

1. a*lpina* Brandegee: Twisselmann et al. 57886, California USA (-24.5, NY); Twisselmann et al. 17270, California USA (-25.8, NY).
2. *ameliae* Lundell: Correll & Rollins 23934, Texas USA (-27.5, NY); Lundell & Lundell 12807, Texas USA (-27.6, NY).
3. *ammophila* Greene: Anderson 1241, Wyoming USA (-26.0, USA); Dorn 4426, Wyoming USA (-26.4, NY).
4. *angustifolia* Green: Muerer-Grimes & Grimes 34, Texas USA (-27.5, NY); Spellenberg 3996, New Mexico USA (-26.5, NY).
5. *bigelovii* Heim.: Barneby 3074, New Mexico USA (-24.5, NY); Spellenberg & Soreng 6481, New Mexico USA (-24.5, NY).
6. *carletonii* Coult. & Fisher: Spellenberg 4677, New Mexico USA (-26.5, NY); Spellenberg & Zucker 12421, New Mexico USA (-28.1, NY).
7. *elliptica* Nelson: Tiehm 3425, Nevada USA (-24.5, NY); Tiehm & Nachlinger 13287, Nevada USA (-26.8, NY).
8. *fragrens* Nutt. ex Hook.: Higgens 17146, Texas USA (-28.1, NY); Spellenberg 13669, New Mexico USA (-28.1, NY).
9. *grabrifolia* Standl.: Cronquist 11427, Colorado USA (-25.1, NY); Neese 15788, California USA (-26.9, NY).
10. *latifolia* Eschsch.: Clausen 4919, California USA (-25.3, NY); Crampton 3120, California USA (-24.9, NY).
11. *macrocarpa* Galloway: Atha 403, Texas USA (-29.3, NY); Orzell & Bridges 16223, Texas USA (-29.7, NY).
12. *maritima* Nutt.: Denton 2263, California USA (-27.3, NY).
13. *mellifera* Douglas .: Halse 3448, Oregon USA (-24.9, NY); LeDoux & Dunn 956, Washington USA (-27.5, NY).
14. *nana* S. Wats.: Niles & Holland 3563, Nevada USA (-26.4, NY); Welsh et al. 16802, Utah USA (-26.9, NY).
15. *nealleyi* Standl. (may = *A. carletonii*): Johnston 5723, New Mexico USA (-27.4, NY).
16. *orbiculata* Standl.: Clokey 7920, Nevada USA (-27.1, -25.2, NY).
17. *pogonantha* Heim.: Eleis et al. 9216, California USA (-25.8, NY); Everett & Balls 21568, California USA (-26.9, NY).
18. *turbinata* Torr.: Tiehm 5860, Nevada USA (-24.7, NY); Tiehm & Tucker 7756, Nevada USA (-25.1, NY).
19. *umbellata* Lam: Moran 792, California USA (-26.9, NY).
20. *villosa* Wats.: Breedlove 60660, Baja California MX (-26.2, NY); Thorne et al. 57886, Baja California MX (-24.5, NY); Wiggens 14014, California USA (-24.1, NY).

Excluded

brevifolia Standl. (=*B. umbellata*): Parks 24212, California USA (-27.2, NY).

Allionia
1. *choisyi* Standl.: Douglas 2132, Arizona USA (-14.6, NY); Douglas 2138, New Mexico USA (-13.8, NY); Lundell 5124, San Luis Potosi MX (-11.8, K); Molina & Molina 24975, Guatemala (-14.6, MO); Sherman et al. 115, Texas USA (-13.2 MO); Skehan 102, New Mexico USA (-11.5, K); Spellenberg 4896, New Mexico USA (-13.5, NY); Worthington 14713, New Mexico USA (-13.7, NY).
2. *incarnata* L.: Asplund s.n., Pichincha Ecuador (-12.6, K); Brizuela 902, Cordoba Argentina (-12.2, NY); Goodspeed 17359, Lima Peru (-11.6, K); Hammond 11618, Arizona USA (-13.9, MO); Higgens 12362, Texas USA (-13.6, NY); Hitchcock & Muhlick, California USA (-12.9, K); Solomon & Stein 11633, Bolivia (-13.6, NY); Windham 89-42, Arizona USA (-15.0, MO).

Excluded:

banduriae Phil. (= *A. incarnata*): Morong 1102, Chile (-13.5, NY); Phillips 2-1888, Chile (-12.2, K).
cristata Standl.: (= *A. incarnata*): Luck, July 15 1896, Arizona, USA (-11.5, K).
glabra Standl. (= *A. choisyi*): Lundell 5124, Charcas MX (-11.8, K); Skehan 102, New Mexico USA (-11.8, K).
puberula Phil. (=*A. incarnata*): Hawkes et al. 4632, Bolivia (-13.0, MO); Solomon & Stein 11633, La Paz Bolivia (-13.9, MO).

Andradea floribunda Allemão: Duarte 9727, Brazil (-26.0, K); Kuhlman 312, Minas Gerais Brazil (-26.0, MO).

Anulocaulis
1. *annulatus* Standl.: Peebles 267, California USA (-26.0, MO); Jones May 3, 1987, California USA (-24.2, MO).
2. *eriosolenus* Standl.: Douglas 2196, Couhuila MX (-26.6, NY); Palmer 1124, Coahuilla MX (-24.3; K); Parks 1068, Texas USA (-23.9, MO); Powell 2177, Texas USA (-25.2, MO); Reveal & Atwood 3272, Chihuahua MX (-27.0, NY).
3. *gypsogenus* Waterfall: Correll & Johnston 20353, Texas USA (-25.5, MO); Waterfall 7809, Texas USA (-26.8, MO).
4. *leiosolenus* Standl.: Douglas 2078, New Mexico USA (-24.5, NY); Hess & Vuono 14 May 1998, New Mexico USA (-25.2, MO); Powell 2389, Texas USA (-25.8, MO); Ricketson 1086, Arizona USA (-25.2, K); Train 1811, Nevada USA (-24.0, MO).
5. *reflexus* Johnston: Hendrickson 7699, Ojinaaga MX (-29.7, NY); Johnston 5296, Texas USA (-26.3, MO).

Acleisanthes
1. *acutifolia* Standl.: Palmer 282, Coahuila MX (-23.6, MO); Reveal & Hess 3008, Chihuahua MX (-28.0, MO).
2. *angustifolia* R.A. Levin: Corell & Correll 30518, Texas USA (-25.8, MO); E. Palmer 1119, Coahilla MX (-21.9, K).
3. *anisophylla* Gray: Parry et al. 1124, Texas USA (-25.8, NY).
4. *chenopodoides* R.A. Levin: Douglas 2137, New Mexico USA (-27.0, NY); Pringle 1885, Chihuahua MX (-25.6, K); Wooton 408, New Mexico USA (-23.3, K).
5. *crassifolia* Gray: Spellenberg & Moore, Texas USA (-28.0, NY).
6. *diffusa* R.A. Levin: Palmer 13795 (-24.5, MO); Wright 1708, New Mexico USA (-25.7, K).
7. *lanceolata* R.A. Levin: MBG 815599; Texas USA (-25.5, M0); Waterfall 4474, Texas USA (-25.5, MO).
8. *longiflora* A. Gray: LeSueur 27, Chihuahua MX (-25.8, MO); Palmer 187, Coahuilla MX (-25.3, K); Pringle Aug 1885, Chihuahua MX (-24.3, K); Wynd & Mueller, Coahuila MX (-26.2, K).
9. *nevadensis* Turner: Macquire & Blood 4394, Nevada USA (-22.0, USA); Williams & Thiem 80-33-12; Nevada USA (-26.0, MO).
10. *obtusa* Standl.: Fisher 44212, Nuevo Leon MX (-26.1, MO); Lieb 494, Texas USA (-26.8, NY); Zamudio 3261, Baja California MX (-22.7, MO).
11. *parviflora* R.A. Levin: Warnock 21751, Texas USA (-25.5, MO).
12. *wrightii* Benth. & Hook.: Corell & Johnson 19433, Texas USA (-27.3, NY); Parks 295, Texas USA (-25.7, MO); Parry 773, New Mexico USA (-23.7, K).

13. *somalensis* Choiv.: Thulin 5642, Somalia (-25.4, K).
14. *undulatus* Fowler & Turner: Gregg 1848, Texas USA (-26.1, MO).
Excluded
A. greggi Standl. (=*A. obtusa*): Fryxell 3663 (-27.9, K); Palmer 282 (-25.2, K).

Belemia fucsiodes Pires: Belen 3796, Minas Gerais Brazil (type specimen with C_3 anatomy, MO).

Boerhavia
1. *acutifolia* J. Moore: Lorence et al. 8401; Hawaii USA (-13.7, MO).
2. *alata* Watson: Douglas 2156A, Sonora MX (-14.3, NY); Douglas 2156B, Sonora MX (-15.7, NY); Spellenberg & Wilson 3627, Sonora MX (-12.7, MO).
3. *albiflora* Fosb.: Fosberg 55761 Pheonix Isl (-12.2, K); Fosberg & Stoddert 54832, Pheonix Isl (-12.7, MO); Fosberg & Stoddert 54864, Sydney Isl (-12.9, K).
4. *anisophylla* Torr.: Douglas 2194, Durango MX (-14.0, NY); Palmer 156, Saltillo MX (-12.2,K); Powell & Powell 3614, Texas USA (-14.2, MO); Pringle 685, Chihuahua MX (-13.6, K); Robbins & Roby 74153 Nuevo Leon MX (-13.5, MO).
5. *ciliata* Brandegee: Douglas 2145, Texas USA (-13.9, NY).
6. *coccinea* Mill.: Atwood 17075, California USA (-13.8, MO); Atwood & Welsh 25500, Arizona USA (-15.5, NY); Carter 296, Afganistan (-13.0, K); Douglas 2056, Arizona USA (-14.9, NY).
7. *cordobensis* Kuntze: Garcia 917, Argentina (-12.7, NY); Germishuizen 8426, South Africa (-13.0; MO); O'Donnel 4420, Argentina (-12.7, NY); Retief 27, South Africa (-13.4 K); Retief 111, South Africa (-13.6, K); Zietsman et al. 4105, South Africa (-13.0, MO).
8. *coulteri* Watson: Atwood 17074, California USA (-14.8, MO); Douglas 2124, Arizona USA (-14.7, NY); Douglas 2127, Arizona USA (-17.0, NY); Makings 2516, Arizona USA (-13.8, MO); Niles 3619, Nevada USA (-14.1, NY).
9. *crispifolia* Fosb.: Fosberg 48976, Aldabra Isl (-13.5, MO); Fosberg 49171, Aldabra Isl (-12.5, MO); Fosberg 49223, Aldabra Isl (-12.5, K); Renvoize 781, Aldabra Isl (-12.4, K).
10. *deserticola* Codd.: Giess et al. 6243, Namibia (-12.0, MO); Long & Rae 745, Namibia (-13.3, MO); Merxmüller & Giesss 30622, Namibia (-13.6, K); Müller & Giesss 357, Namibia (-13.6, K).
11. *diandra* L.: Ash 2474, Ethiopia (-13.5, MO); Hadidi & Ghannour 4 July 1967, Egypt (-13.7, MO).
12. *diffusa* L.: Acocks 18813, Namibia, (-12.3, K); Burkhalter 15965, Florida USA (-15.4, MO); Furuse 3149, Okinawa (-11.4, K); Germishuizen 2527, Namibia (-13.7, K); Heppes 5675, Yemen (-12.3, K); MaConochie 12-11-01, Oman (-13.0, K); Thomas 455, Louisiana USA (-14.9, MO).
13. *dominii* Meikle & Hewson: Adams 3946, New South Wales, AUS (-12.7, K); Lepschi 730, AUS (-13.6, K); Lucas & Wilson 16, Northern Territory AUS (-13.2, MO); Pedley 5382, Queensland AUS (-14.2, MO).
14. *elagans* Choisy: Abedni & Hassan 6474 Pakistan (-12.7, MO); Ali et al. 1464, Pakistan (-12.7, MO); Grey-Wilson 302, Iran (-13.9, K); Lawton 2302, Oman (-14.1, K); Wykeham & Perry 6/78, Arabia (-11.6, K).
15. e*recta* L.: Blumer 1765, Arizona USA (-12.5, K); Bos 5019, Cameroon (-13.4, K); Kral 53991, Florida USA (-14.5, MO); Reckmans 6478, Burundi (-15.8, K); Thulin & Dahir 6426, Somalia (-13.0, K); Warnock et al. 46368, Texas USA (-13.6, MO).
16. *glabrata* Blume: Florence 9383, Marquesas Isl (-13.3; K); Fosberg 13889 (-12.2, K).
17. *gracillima* Heim.: Douglas 2143, Texas USA (-14.3, NY); Gentry 1603, Rio Mayo MX (-12.8, K); Sherman et al. 145, Texas USA (-13.7, MO); Wiggens 15311, Baja California MX (-12.8; K).
18. **grahamii* Gray: Jones 4194, Texas USA (-12.0, NY).[1]
19. *herbstii* Fosb.: Herbst 6308, Hawaii USA (-12.8, K).

---

*[1] This name is a synonym of *Commicarpus scandens* (L.) Standl. Close examination shows the specimen belongs to the genus *Boerhavia* based on mporphology, while $\delta^{13}C$ values confirm that the specimen is a C_4 species. We thus retain the name on the herbarium sheet.

20. *heroensis* Heim.: Hardy 2025, Namibia (-13.1, K); Hardy & de Winter 1423, South Africa (-13.8, MO); Seydal 4323, Namibia (-13.3, MO); Tolken & Hardy 810, Namibia (-12.6, K).
21. **intermedia*[2] Jones: Beckett 10015, Arizona USA (-12.0; K); Carter & Kellogg 2903, Baja California MX (-11.9, K); Douglas 2174, Sonora MX (-13.6, NY); Douglas 2186, Sonora MX (-14.7, NY); Hutchinson 6812, California USA (-12.6, K); Wiggens, 15298, Baja California MX (-12.2, K); Rickettson & Vanderbur 1922, Arizona USA (-13.9, MO); Sherman et al. 153, Texas USA (-14.1, MO).
22. *lateriflora* Standl. Douglas 2157, Sonora MX (-14.4, NY); Douglas 2166, Sonora MX (-14.3, NY).
23. *linearifolia* Gray: Barnaby 2584, Arizona USA (-12.8, NY); Douglas 2205, Coahuila MX (-14.0 NY); Earle & Earle 379 New Mexico USA (-13.2, K); Groth 98, Texas USA (-12.3, K); Miller et al., 5062, Texas USA (-14.5, MO); Spellenberg & Moore 2608, Texas USA (-12.9, MO).
24. *maculata* Standl.: Gentry 4060, MX (-12.1, K).
25. *pulchella* Grisebach: Bruch-Carette 11/11914, Argentina (-10.5, NY); Espinar 2087, Argentina (-12.6, NY).
26. *purpurescens* Gray: Blumer 1672, USA (-11.9; K); Douglas 2149, Arizona USA (-14.3, NY); Spellenberg & Brouillet 13261, Arizona USA (-14.0, NY); Wiggens & Rollins 385, Sonora MX (-12.0, MO).
27. *pterocarpa* S. Wats.: Douglas 2178, Arizona USA (-13.6, NY); Gibbs & Robinson 511, South Africa (-13.8, MO); Gould 3266, Arizona USA (-12.6, MO); Herman 611, South Africa (-12.4, K); Leistner 938, South Africa (-12.7, K).
28. *repens* L.: Khan & Huq 3934, Bangladesh (-12.2, MO); Miller 324, Yemen (-13.0, K); Nowicke et al. 351, Sri Lanka (-13.3, MO); Stoddert 8093, Aldabra Isl (-13.1, NY); Wood 3231, Yemen (-12.9, K).
29. *[3]*scabrida* Steudel: Zöllner 8553 Chile (-13.0, MO).
30. *schomburgkiana* Oliv.: Lazarides & Palmer 006, AUS (-13.5, K); Zich 125, Western AUS (-14.0, MO).
31. *spicata* Choisy: Douglas 2171, Sonora MX (-13.6, NY); Douglas 2176, Sonora MX (-14.7, NY); Earle & Earle 316, New Mexico USA (-10.9, K); Makings 2509, Arizona USA (-14.1, MO); Sherman et al. 76, Arizona USA (-14.1, MO); Wiggens, 14674, Baja California MX (-12.6, K); Wiggens 15647, Baja California MX (-12.7, K); Wooton 407, New Mexico USA (-13.2, K).
32. *tetrandra* Forst.: Fosberg & Stoddert 54820, Pheonix Isl (-12.1, K); Judd & Mitchell 57, Jarvis Isl (-11.5, K); Sachet 1398, Tuamotu Isl (-12.8, MO); Sachet 1973, Society Isl (-12.1, MO); Whistler W5307, no place name (-13.4, K).
33. **tomentosa*[4] Ehrenb.: Rottler s.n., India (-9.4, K).
34. *torreyana* Standl.: Douglas 2146, Texas USA (-14.0, NY); Fosberg S3282, New Mexico USA (-13.2, MO); Higgens 20767, Arizona USA (-15.2, NY); Kusche Aug 1927, Arizona USA (-11.8, K); Palmer 488, Coahilla MX (-12.3, K).
35. *traubae* Spellenberg: Douglas 2169, Sonora MX (-12.7, NY); Douglas 2170, Sonora MX (-11.9, NY).
36. *triquetra* S. Wats,: Carter et al. 1887, Baja California MX (-12.7, K).
37. *verbenacea* Killip.: Beetle 26195, Peru (-12.1, K); Vega 3097, Peru (-12.9, NY); Vega & Guevara 6182, Peru (-13.4, NY).
38. **verticillata*[5] Poir.: Klackenberg & Landon 142, India (-13.5, K); s.n. 3699 (MO #2230899), Pakistan (-13.5, MO).
39. **viscosa*[6] Laq. & Rodr: Baker 65, Nicaragua (-12.8, NY); Barnes & Lund, 202, Jalisco MX (-11.8); Jacques-Georges 13132, Mauritania (-12.1, MO); Nash 2466, Florida USA (-11.2, K); Pittier 114, Guatemala (-13.0, NY); Smith 4601, Guatemala (-12.5, K); Wooton 421, New Mexico USA (-12.7, K). (Likely *B. coccinea*)

---

[2] The name is a synonym of *B. triquetra* S. Watss.
[3] This name of this species is a synonym of *B. erecta* L.
[4] This name is a synonym of *B. repens* L.
*[5] Tropicos, 2018, lists this species as *Commicarpus boissieri* Cufod. Current classification puts it as a synonym of *Commicarpus plumbagineus* (Cav.) Standl. However, the $\delta^{13}C$ value shows this specimen is C_4, possibly a misidentified *Boerhavia* species. We kept the name on the herbarium sheet for tracability and further investigation.
*[6] This species is a synonym of *Boerhavia coccinea* Mill.

40. *vulvarifolia*[7] Poir.: Jacques-Goerges 12357, Senegal (-11.7, MO).
41. *weberbaueri* Heim.: Sanchez 3082, Peru (-12.9, MO); Sanchez et al. 6182, Peru (-12.8, MO).
42. *wrightii* Gray: Brant & Stone 3980, Arizona USA (-14.3, MO); Doan 1308, Sonora MX (-14.5, NY); Gould & Haskell 3193, Arizona USA (-12.6, MO); Palmer 845, Carmen Isl MX (-10.6, K).
43. *xantii* S. Wats: Anderson 12548, Sonora MX (-13.7, MO); Carter et al. 1975, Baja California MX (-12.9, K); Douglas 2154, Sonora MX (-14.2, NY); Douglas 2249, Sonora MX (-14.6, NY); Flores et al. 247 Baja California MX (-14.4, MO); Gentry 1593, Rio Mayo MX (-12.3, K); Gentry 4724, Sonora MX (-12.8, MO).

Excluded

B. caribaea Jacq (= *B. coccinea*): Bentley 101, Galapagos Isl (-12.9, MO); Manterrosa & Carballo JMS00373, El Salvador (-13.8, MO).

B. chinensis Asch & Schweinf. (=*Commicocarpus chinensis*): Geesink et al. 7931, Thailand (-32,3; K).

B. decumbens Vahl. (=*B. coccinea*); Moldenke 775, Florida USA (-12.8, K).

Boldoa *purpuescens* (see *Salpianthus purpurescens*)

Cephalotomandra *fragrans* Karst. & Triana: Triana 998, New Granada (-28.8, K).

Colignonia

1. *glomerata* Griseb.: Britton & Rusby 699, Bolivia (-27.4, K); Hawkes et al. 3466, Argentina (-29.9, MO).
2. *ovalifolia* Heim.: Harling et al 10406, Ecuador (-30.2, MO); Pearce 718, Bolivia (-26.2, K).
3. *parviflora* Choisy: Andre 3254, Ecuador (-25.1, K); Bernal 1282, Colombia (-25.7, MO); Gentry et al. 75078, Peru (-25.6, MO); Stork et al. 10621, Peru (-24.7, K).
4. *pentoptera* Bohlin: Mexia 7713, Ecuador (-26.4, K; -27.8, MO).
5. *rufopilosa* Kuntze: Cevado-Rodriguez 6578, Bolivia (-29.1; K); Cabanillas 59, (-27.7, MO).
6. *scandens* Benth.: Harling & Anderson 13651, Ecuador (-27.3, MO); Stork & Horton 10801, Peru (-24.8, K).

Commicarpus

1. *ambiguous* Meilke: Wood 2749, Yemen (-26.6; K).
2. *arabicus* Meikle: Miller 194, Yemen (-24.7, K); Spellenberg 7507, Yemen (-28.7, NY); Thulin et al. 9294, Yemen (-27.3, K).
3. *australis* Meilke: Hill 584, AUS (-27.1, K); Wilson 12593, AUS (-27.1; K).
4. *boissieri* Cufod.: Collinette 7121, Saudi Arabia (-26.3, K); Thulin & Gifri, 8510, Yemen (-29.9, K).
5. *chinensis* Heim.: Fosberg 41316, Heron Isl AUS (-27.6, NY); Latz 9452, AUS (-28.0, K); Strey 5404, Natal South Africa (-28.0, K).
6. *decipiens* Meilke: Tolen & Hardy 737, Namibia (-25.4, K).
7. *fallacissimus* Heim.: de Winter 3051, Namibia (-24.5, K); Hardy & de Villiers 5129, South Africa (-26.9, K); Seydal 1350, Namibia (-24.7, NY).
8. *heimerlii* Meilke.: Smith & Lavranos 75, Socotra (-26.6, K).
9. *helenae* Meilke: Radcliffe-Smith 3735, Oman (-26.7, K); Radcliffe-Smith 3913, Oman (-28.6, NY); Spellenberg & Abushear 7380, Yemen (-24.5, NY); Wood 2061, Yemen (-24.3, K).
10. *grandifloras S*tandl.: Collenette 1084, Saudi Arabia (-24.6, K); Spellenberg 7365, Yemen (-27.1, NY); Thulin et al. 9311, Yemen (-29.3, K).
11. *greenwayi* Meilke: Polhill & Paulo 2033, Tanzania (-26.0, K); Richards, 21004, Tanzania (-28.1, K).
12. *pedunculosus* Cufod.: Bisset 80, Yemen (-28.1, K); Burney et al. T89, Kenya (-28.4, NY); Gilbert 1981, Kenya (-25.6, NY); Radcliffe-Smith 4975, Yemen (-24.8, K); Richards 23405, Tanzania (-24.0), NY).
13. *pentandrus* Heim.: Bayliss 3715, Swaziland (-27.3, NY); Immelkan 2732CA, Natal South Africa (-30.9; K); Merxmuller & Giess 788, Namibia (-26.3, NY); Reid 2827CA, South Africa (-24.2, K): Seydal 4162, Namibia (-24.6, NY); Stirton 8848, Natal (-27.2, K).
14. *pilosus* Meilke: Davies 2363, Zimbabwe (-27.7, K); Leach 10664, Zimbabwe (-26.5, K).

*[7] This species is classified as a synonym of *Boerhavia repens* L.

15. *plumbagineus* Standl.: Agnus 3044, Rhodesia (-26.1, NY); Boulos & Ads 14218, Saudi Arabia (-26.8, K); Burney & Burney N248, Kenya (-28.8, NY); Radclifffe-Smith & Henchie 4551, Yemen (-26.4, K); Reekmans 7685, Burundi (-28.8, K); Spellenberg & Abushear 7374, Yemen (-26.4, NY); Tanner 595, Tanzania (-25.6, NY).
16. *raynalii* Lebrun et Meilke: Fotius 1233, Chad (-26.1, K).
17. *reniformis* Cufod.: Radcliffe-Smith 4227, Oman (-28.8, K); Thulin et al. 8337, Yemen (-27.8, K).
18. *scandens* Standl.: Breteler 4357, Nerida, Venezuela (-27.1, NY); Douglas 2108, Arizona USA (-26.9, NY); Nagelkerken 120; South Africa (-27.0, K); Nunez & Colin 2847, Michoacan MX (-25.6, MO); Pringle 1884, Arizona USA (-24.4, K); Rothchild & Ayala 76, Jalisco MX (-29.8, K); Spellenberg 3714, Texas USA (-28.1, NY); Stoffers 2073, South Africa (-25.2, K).
19. *simony*i Meikle: Seydal 451, South Africa (-27.4, K); Smith & Lavranos 176, Socotra (-24.8, K); Smith & Lavranos 223, Socotra (-27.7, K).
20. *sinuatus* Meikle: Miller 8 (-26.0, K); Spellenberg 7144, Yemen (-25.0, NY); Spellenberg 7506, Yemen (-30.8, NY); Wood 2603, Yemen (-27.9, K).
21. *squarrosus* Standl.: Boulos et al. 17003, Yemen (-26.8, K); Merxmuller & Giess 1704, Namibia (-24.9, NY); Wood 1605, Yemen (-24.1, K).
22. *stenocarpus* Cufod.: Grey-Wilson 280, Iran (-26.9, K); Thulin et al. 8222, Yemen (-30.5, K).
23. *tuberosus* Standl.: Hutchinson 1408, Peru (-27.8, K); Madsen 63910, Ecuador (-29.4, NY); Plowman & Alcorn 14329, Ecuador (-29.9, NY); Sagastegui 15288, Peru (-30.0, NY); Wood 4473, Columbia (-25.3, K).

***Cryptocarpus** pyriformis* Kunthe: Plowman & Alcorn 14321, Guayas Ecuador (-25.6 K); Weberbauer 7747, Peru (-23.3, K); Wiggens & Porter 590, Galapagos Isl (-23.4, K).

Cyphomeris
1. *crassifolia* Standl.: Douglas 2203, Coahuila MX (-27.7, NY); Miller et al. 5735, Texas USA (-29.7, MO); Muller 2094, Nuevo Leon MX (-27.2, MO); Spellenberg et al. 5938, Neuvo Leon MX (-29.2, MO); Warnock & Barkley 14871, Coahuila MX (-26.9, MO).
2. *gypsophiloides* Standl.: Mahrt et al. Oct. 13, 1990, Texas, USA (-28.8, MO); Marht et al. 110, Texas USA (-27.2, MO); Robbins & Roby 74183, Coahuila MX (-28.0, NY); Spellenberg & Zucker, 2 Sept 1987, New Mexico USA (-27.1, NY); Spellenberg et al. 4077, Zacatecas MX (-27.1, MO); Spellenberg et al. 5930, Texas USA (-27.6, MO); Vasey 1881, New Mexico USA (-24.9, K).

***Grajalesia** fasiculata* Miranda: Linares & Martinez 2731, El Salvador (-25.7, MO).

***Leucaster** caniflorus* Choisy: Glaziou 12115, Rio de Janeiro Brazil (-28.8, K).

Mirabilis
1. *aggregata* Cav.: Sherman et al. 255, Texas USA (-31.2, MO); Mueller 7995, Texas USA (-25.7, MO).
2. *albida* Heim.: Lammers 10718, Missouri USA (-29.6, MO); Rowan 1195, Missouri USA (-29.9, MO).
3. *alipes* Pilz.: Blair & Thiem 326, Nevada USA (-26.3, NY); Pinzl 11311, Nevada USA (-26.2, NY).
4. *austrotexana* Turner: Correll 36778, Texas USA (-25.2, NY); Turner 5164, Texas USA (-28.4, NY).
5. *coccinea* Benth. & Hook.: Daniel 15777, Arizona USA (-26.3, NY); Landrum et al. 9789, Arizona USA (-24.7, NY); Reeves & Lehto L20180, Arizona USA (-28.7, NY).
6. *decipiens* Standl.: Mooers 514, Colorado USA (-28.3, NY); Rollins 1545, Colorado USA (-26.0, USA).
7. *glabra* Standl.: Hazlett 9895, Colorado USA (-29.3, NY).
8. *greenei* S. Wats: Bonar et al. 22 May 1949, California USA (-29.5, NY); Pilz 994, California USA (-27.7, NY).
9. *jalapa* L.: Croat 2842, Madagascar (-23.3, K); Nee & Atha 44052, Texas USA (-29.0, NY); Stoddart 7208, Amirante Isl (-29.4, K); Thomas & Amason 142,066, Arkansas USA (-30.9, NY).
10. *laevis* Curran.: Abrams 2504, California USA (-25.5, K); Barneby 18303, Oregon USA (-26.5, NY); Duran 3455, California USA (-23.5, K); Epling & Robison 4-1932 (-26.1, K); Erter 5834, California USA (-26.7,

NY); Howell 8333, Guadalupe MX (-22.9, K); Spellenberg 12336, California USA (-28.5, NY); Strong & Strong March 3, 1960, Arizona USA (-24.6, K); Williams & Tiehm 76-111-2, Nevada USA (-27.0, NY).

11. *linearis* Heim. Alexander 1183, Utah USA (-27.8, NY); Higgens 7273, Oklahoma USA (-29.4, NY).
12. *longiflora* L.: Douglas 2092, New Mexico USA (-29.2, NY); Spellenberg 3832, New Mexico USA (-26.4, NY).
13. *Macfarlanei* Constance & Rollins: Kaye 1351, Oregon USA (-26.0, NY); Pilz 1000, Oregon USA (-25.0, NY).
14. *melanotricha* Spellenb.: Douglas 2075, New Mexico USA (-27.6, NY); Spellenberg & Ward 9729, New Mexico USA (-29.5, NY).
15. *multiflora* Gray: Higgens 8623, New Mexico USA (-23.8, NY); Holmgren 3355, Arizona USA (-24.9, NY).
16. *nyctaginia* MacMill.: Holmgren 9772, New York USA (-30.1, NY); Nee 43870, New York USA (-28.9, NY).
17. *oligantha* Macbr.: Wiggens 15940, Baja California MX (-24.0, K).
18. *oxybaphoides* Gray.: Metcalfe Sept 15 1903, New Mexico USA (-27.4, K).
19. *polyphylla* Standl.: Wiggens 4406, Baja California MX (-22.6, K).
20. *sanguinosa* Heim.: Hinton et al. 11976, Zitacuaro MX (-27.5, K).
21. *tenuiloba* Wats.: Epling & Robinson, April 8, 1932 (-26.1, K); Wiggens 15863, Baja California MX (-23.8, K).
22. *triflora* Benth.: Carter 2695, Baja California MX (-28.6, K).
23. *wrightiana* Gray: Gentry 2654, Rio Mayo MX (-27.5, K).

Excluded

M. comata Standl. (=*M. albida*): Barneby 18120, Arizona USA (-26.1, NY); Douglas 2035 Arizona USA (-25.9, NY).

M. decumbens Daniels (= *M. linearis*): Robbins 2238, Oklahoma USA (-27.0, NY); Spellenberg & Ward, Texas USA (-27.7, NY).

M. diffusa Reed (=*M. linearis*): Hill 12125, New Mexico USA (-29.1, NY); Spellenberg et al. 3412, New Mexico USA (-26.9, NY).

M. exaltata Standl. (=*M. glabra*): Higgens 8314, Texas USA (-27.4, NY); Wilson 1-6, New Mexico USA (-26.4, NY).

M. gausapoides Standl. (=*M. linearis*): Higgens 5651, Texas USA (-27.6, NY); Higgens 9198, New Mexico USA (-27.7, NY).

M. grayana Heim. (=*M. albida*): Correll 33830, Texas USA (-24.9, NY).

M. hirsuta Macmillan (=*M. albida*): Ferris 157, Nebraska USA (-27.2, NY); Thorne 12198, Iowa USA (-26.8, NY).

Nyctaginia *capitata* Choisy: Barkely 14501, Texas USA (-24.2, K); Bush 1254, Texas USA (-25.8; K); Cerda 372, Texas USA (-28.6, MO); Douglas 2181, Saltillo MX (-26.0, NY); Mcvaugh 7509, Texas USA (-25.1, K); Miller et al. 5053, Texas USA (-27.5, MO); Nelson 4668, Durango MX (-26.7, K); Palmer 294, Durango MX (-26.0, K).

Okenia
1. **grandiflora*[8] Standl.: Hinton et al. 10323, Montes de Oca MX (-12.4, K); Hinton et al. 10346, MX (-13.4, K).
2. *hypogaea* Schltdl. & Cham.: Douglas 2206, Veracruz MX (-13.9, NY); Gilliss 9666, Florida USA (-12.5, MO); Hinton et al. 10868, Caleana MX (-12.3, K); Lott 3955, Jalisco MX (-13.9, K); McKee 10971, Tampico MX (-11.8, K); Reed 104136, Florida USA (-13.1, MO); Small 8501 Caleana MX (-12.3, K); Small & Carter 27 Oct. 1906, Florida USA (-12.1, NY).
3. **parviflora*[9] Wilson: Hinton 6119, Goyuca MX (-12.5, NY); Hinton 6260, Goyuca MX (-12.1, NY); Hinton et al. 6277, MX (-13.4, K); Hinton et al. 8-16-37, Mina MX (-11.2).
4. **rosei* Standl[10].: Fryxall 1729, Michoacan MX (-14.0, NY).

Phaeoptilum *spinosum* Radlk.: de Winter & Leistner 5744, Namibia (-19.6, K); Hardy & Bayliss 1205, South Africa (-29.7, K); Ortendahl 518, Namibia (-22.9, MO); Seydal 417, Namibia (-24.3, MO); Strey 2401, Namibia (-25.4, K).

Pisoniella *arborescens* Standl.: Hinton et al. 13262, Michoacan MX (-26.6, MO); O'Donell 5334, Salta Argentina (-26.4, K); Pringle 11142, Michoacan MX (-24.8, K); Pringle 11697, Federal District MX (-25.2, MO).

Ramisia *brasiliensis* Oliv.: Glazio 14221, Brazil (-24.3, K).

Reichenbachia *hirsuta* Spreng: Hassler 11463, Paraguay (-27.8, K); Nee 35264, Santa Cruz Bolivia (-29.5, K). (=*Reichenbachia paraguayensis* Dugand et Daniel)

Salpianthus
1. *arenarius* Humb & Bonpl.: Guadalupe Ayala 91-16, Jalisco MX (-30.2, K); Hinton 10215, Vallecitos MX (-27.4, K).
2. *macrodontus* Standl.: Carter & Kellogg 3251, Sinoloa MX (-27.4, K).
3. *purpurescens* Hook & Arn.: Hinton 11493, Montes de Oca MX (-30.4, K); Tucker 836, El Salvador (-26.1, K).

Selinocarpus (excluded, species subsumed into *Acleisanthes* as per Levin 2000, 2002).

Tripterocalyx
1. *carneus* Galloway: Muerer-Grimes & Grimes 9 May 1989, Texas USA (-26.7, NY); Wolf & Everett 11411, Arizona USA (-26.1, NY).
2. *crux-maltae* Standl.: Brandegee s.n., California USA (-30.0, K); Pinzl 12878, Nevada USA (-28.1, NY); Tiehm 12213, Nevada USA (-25.0, NY).
3. *cycloptera* Standl.: Hawkes et al. 1220, Chihuahua MX (-25.0, K).
4. *micranthus* Hook.: Higgens 15451, Utah USA (-23.7, NY); Higgens 25483, Utah USA (-28.0, NY); Nelson 7083, Wyoming USA (-22.9, K)

---

*[8] This is a synonym of *Okenia hypogaea* according to Spellenberg (2003). *Flora N. America* vol. 4, pp. 14-74.
*[9] This is a synonym of *Okenia hypogaea* according to Spellenberg (2003). *Flora N. America* vol. 4, pp. 14-74.
*[10] This is a synonym of *Okenia hypogaea* according to Spellenberg (2003). *Flora N. America* vol 4, pp. 14-74.

Supplemental Table S4: Species and NCBI short read archive (SRA) identification numbers for taxa used to generate the phylogenetic tree of the Nyctaginaceae. Data available at http://ncbi.nlm.nih.gov/sra.

Species	SRA ID	Species	SRA ID
Abronia bigelovii	SRR6787503	*Bougainvillea stipitata var grisebachiana*	SRR1594527
Abronia fragrans	SRR6435333	*Colignonia ovalifolia*	SRR6435326
Abronia glabrifolia	SRR6435334	*Commicarpus scandens*	SRR6435360
Abronia latifolia	SRR6435331	*Cyphomeris gypsophiloides*	SRR1979687
Abronia maritima	SRR6435332	*Cypselea humisufa*	ERR2040193
Abronia nealleyi	SRR1979680	*Ercilla volubilis*	SRR1979676
Abronia umbellata	SRR6435337	*Gisekia pharnaceoides*	SRR6435307
Acleisanthes acatitensis	SRR6435338	*Guapira obtusata*	SRR1594257
Acleisanthes acutifolia	SRR6787481	*Hypertelis cerviana*	ERR2040235
Acleisanthes lanceolata	SRR1979679	*Mirabilis jalapa*	ERR2040249
Acleisanthes obtusa	SRR1979678	*Mirabilis jalapa*	ERR2040250
Allionia incarnata	ERR2040243	*Mirabilis multiflora*	SRR1979681
Allionia incarnata	ERR2040244	*Mirabilis pringlei*	SRR6435291
Anisomeria littoralis	SRR6787505	*Neea psychotriodes*	SRR6435290
Anulocaulis annulatus	SRR6435335	*Nyctaginia capitata*	SRR6787479
Anulocaulis eriosolenus	SRR6787488	*Okenia hypogaea*	SRR6435296
Anulocaulis leiosolenus var gypsogenus	SRR1593521	*Phytolacca dioica*	SRR1979683
Anulocaulis leiosolenus var gypsogenus	SRR1593521	*Pisonia aculeata*	SRR1594351
Boerhavia burbidgeana	ERR2040247	*Pisonia umbellifera*	SRR1979682
Boerhavia burbidgeana	ERR2040248	*Rivina humilis*	SRR1594158
Boerhavia ciliata	SRR6435336	*Salpianthus purpurascens*	SRR6435292
Boerhavia coccinea	ERR2040245	*Sarcobatus vermiculatus*	SRR6435321
Boerhavia coccinea	ERR2040246	*Seguieria aculeata*	SRR1594352
Boerhavia coccinea	SRR1698114	*Sesuvium portulacastrum*	ERR2040194
Boerhavia coccinea	SRR1698115	*Sesuvium verrucosum*	ERR2040195
Boerhavia coccinea	SRR1698225	*Sesuvium verrucosum*	ERR2040196
Boerhavia coccinea	SRR1698226	*Trianthema portulacastrum*	ERR2040197
Boerhavia purpurascens	SRR6787486	*Trianthema portulacastrum*	ERR2040198
Boerhavia torreyana	SRR6787485	*Trichostigma octandrum*	SRR6435294
Bougainvillea glabra	SRR1994279	*Tripterocalyx carneus*	SRR6435295
Bougainvillea peruviana	SRR2912686	*Tripterocalyx crux maltae*	SRR6787483
Bougainvillea spectabilis	ERR2040242	*Zaleya pentandra*	ERR2040199

Supplemental Table S5: Genome annotation methods used for specific species**.** *Amar.* Indicates *Amaranthus.*

Genomes with MapMan Annotation	Genomes with all Phytozome Annotation
Amar. hypochondriacus	*Aquilegia caerulea*
Arabidopsis thaliana	*Arabidopsis thaliana*
Daucus carota	*Gossypium raimondii*
Mimulus guttatus	*Medicago truncatula*
Olea europaea	*Mimulus guttatus*
Oryza sativa	*Oryza sativa*
Populus trichocarpa	*Populus trichocarpa*
Setaria italica	*Setaria italica*
Solanum lycopersicum	*Solanum lycopersicum*
Vitis vinifera	*Vitis vinifera*

Supplemental Table S6: Parameter selection by stepwise regression (A) and mixed model design and comparison (B). Two of eight significant predictors, Bio 5 and Bio 19 (highlighted with grey shading) were strongly correlated with Bio 10 and AI respectively, and were thus removed from the mixed models.

(A)	Estimate	Std. Error	z value	Pr(>|z|)
(Intercept)	-40.03	20.60	-1.94	0.05
Annual mean temperature (Bio1)	0.19	0.10	1.95	0.05
Isothermality (Bio3)	-2.59	1.54	-1.68	0.09
Temperature seasonality (Bio4)	3.96	2.84	1.39	0.16
Max temperature of warmest month (Bio5)	9.92	3.82	2.60	0.01**
Annual temperature range (Bio7)	-9.00	3.79	-2.38	0.02*
Mean temperature of warmest Quarter (Bio8)	3.09	1.17	2.65	0.01**
Mean temperature of warmest Quarter (Bio10)	-12.42	4.37	-2.84	0.00**
Mean temperature of coldest Quarter (Bio11)	-7.33	3.63	-2.02	0.04*
Precipitation seasonality (Bio15)	1.61	0.78	2.06	0.04*
Precipitation of driest quarter (Bio17)	-3.24	2.22	-1.46	0.14
Precipitation of warmest quarter (Bio18)	-3.23	1.80	-1.79	0.07
Precipitation of coldest quarter (Bio19)	3.75	1.66	2.26	0.02*
Annual global aridity index AI	4.37	1.90	2.30	0.02*

Signif. codes: 0 ‘***’ 0.001 ‘**’ 0.01 ‘*’ 0.05 ‘.’ 0.1 ‘ ’ 1

(B) Mixed effect logistic models

Model 0: photosynthesis ~ bio8 + bio10 + bio11 + bio15 + AI
Model 1: photosynthesis ~ bio8 + bio10 + bio11 + bio15 + AI + subtype
Model 2: photosynthesis ~ bio8 + bio10 + bio11 + bio15 + AI + subtype + (1 | genus)
Model 3: photosynthesis ~ bio8 + bio10 + bio11 + bio15 + AI + subtype + (1 | species) + (1 | genus)

Model comparison	AIC	Pr(>Chisq)
Model 0	94.05	
Model 1	22	1
Model 2	18	<2e-16***
Model 3	20	1

Supplemental Table S7: The results of phylogenetic ANOVA comparing a subset of C_3 and C_4 species for the six significant bioclimatic variables listed in Supplemental Table S6. Species lists include: *Commicarpus scandens* (C_3), *Cyphomeris gypsophiloides* (C_3), *Allionia incarnata* (C_4), *Nyctaginia capitata* (C_3), *Anulocaulis eriosolenus* (C_3), *Anulocaulis annulatus* (C_3), *Boerhavia ciliata* (C_4), *Boerhavia burbidgeana* (C_4), *Boerhavia torreyana* (C_4), *Boerhavia coccinea* (C_4). The *p* values wre adjusted using the Bonferroni–Holm method. Number of simulations equals 1000. Median values per variable per species were used.

	F value	Pairwise (C_3 versus C_4) corrected *p* values
Annual temperature range (Bio 7)	0.04	0.86
Mean temperature of wettest quarter (Bio 8)	0.93	0.43
Mean temperature of warmest quarter (Bio 10)	0.04	0.88
Mean temperature of coldest quarter (Bio 11)	0.07	0.82
Precipitation seasonality (Bio 15)	0.13	0.76
Annual global aridity index (AI)	0.13	0.77

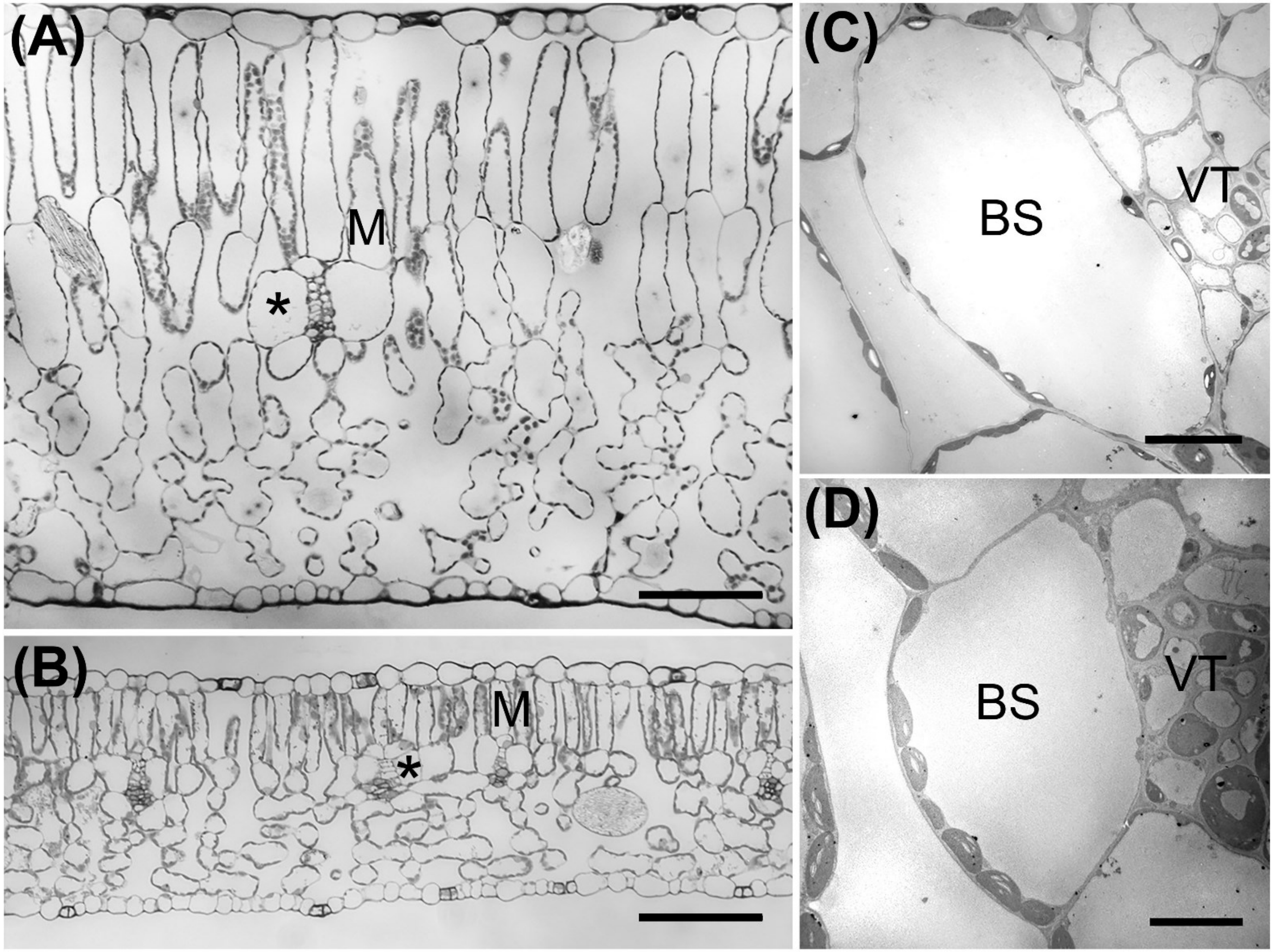

Supplemental Figure S1: Light (A, B) and transmission electron (C, D) microscopy images of the leaf tissue for the C_3 species of Nyctaginaceae, tribe Nyctagineae. (A, C) *Anulocaulis gypsogenus,* (B, D) *Mirabilis jalapa.* Scale bars, 100 µm (A, B); 10 µm (C, D). * marks bundle sheath cell. BS, bundle sheath; M, mesophyll; VT, vascular tissue.

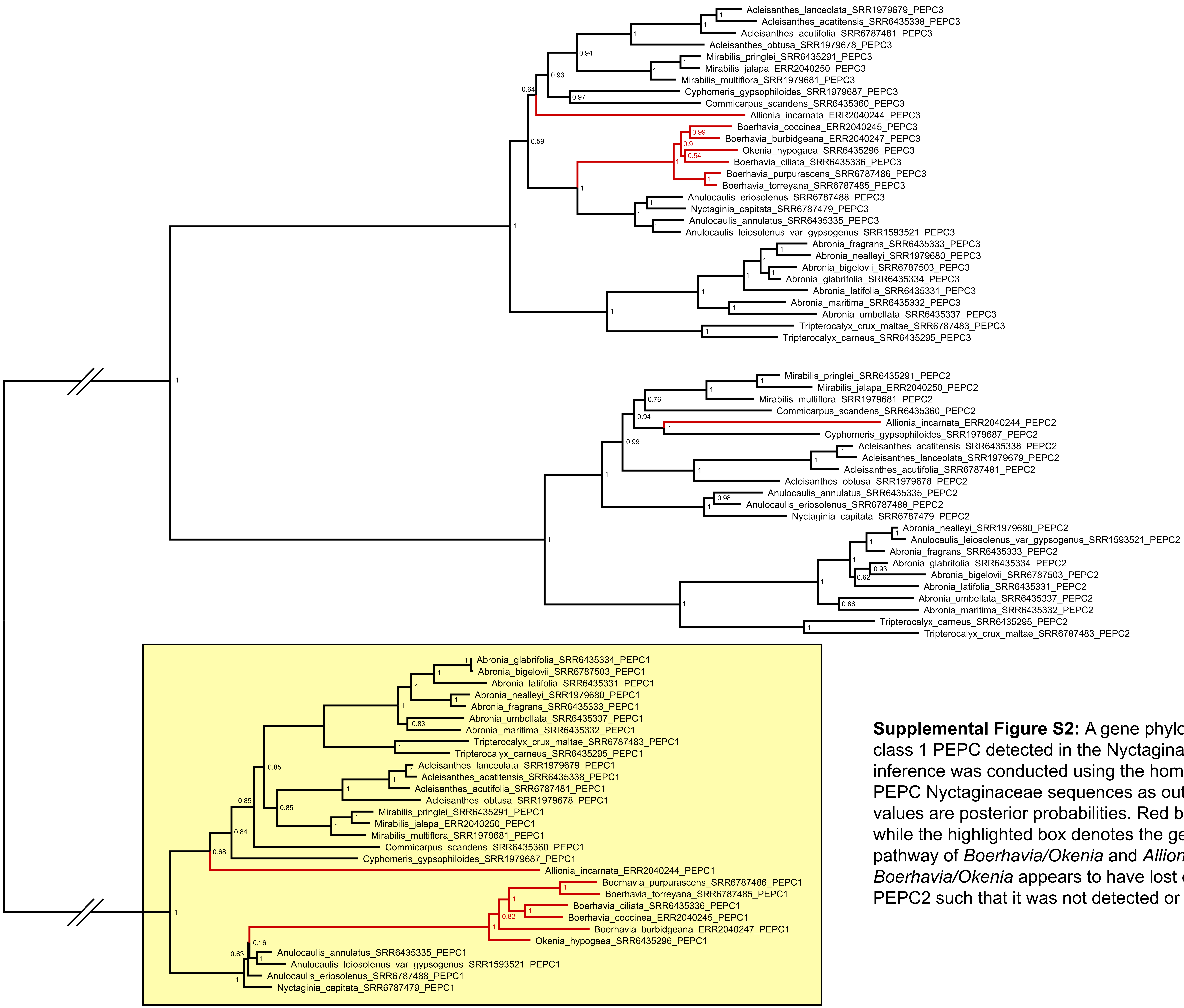

Supplemental Figure S2: A gene phylogeny for the three paralogs of class 1 PEPC detected in the Nyctaginaceae. A Bayesian phylogenetic inference was conducted using the homologous class 2 bacterial-type PEPC Nyctaginaceae sequences as outgroup (not shown). Node values are posterior probabilities. Red branches denote C_4 species, while the highlighted box denotes the gene copy utilized in the C_4 pathway of *Boerhavia/Okenia* and *Allionia incarnata*. Note that *Boerhavia/Okenia* appears to have lost or severely downregulated PEPC2 such that it was not detected or assembled.

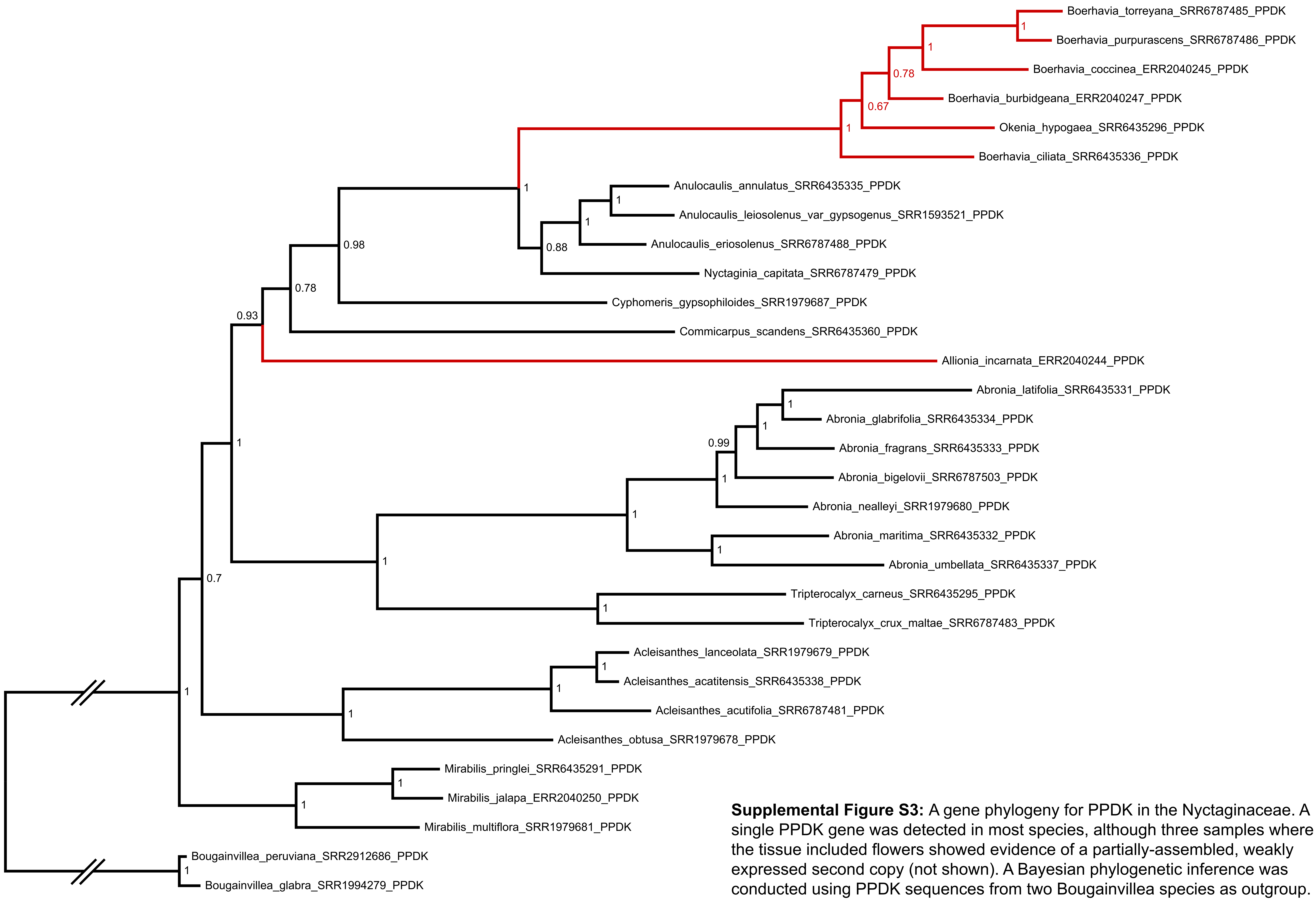

Supplemental Figure S3: A gene phylogeny for PPDK in the Nyctaginaceae. A single PPDK gene was detected in most species, although three samples where the tissue included flowers showed evidence of a partially-assembled, weakly expressed second copy (not shown). A Bayesian phylogenetic inference was conducted using PPDK sequences from two Bougainvillea species as outgroup. Node values are posterior probabilities. Red branches denote C_4 species.

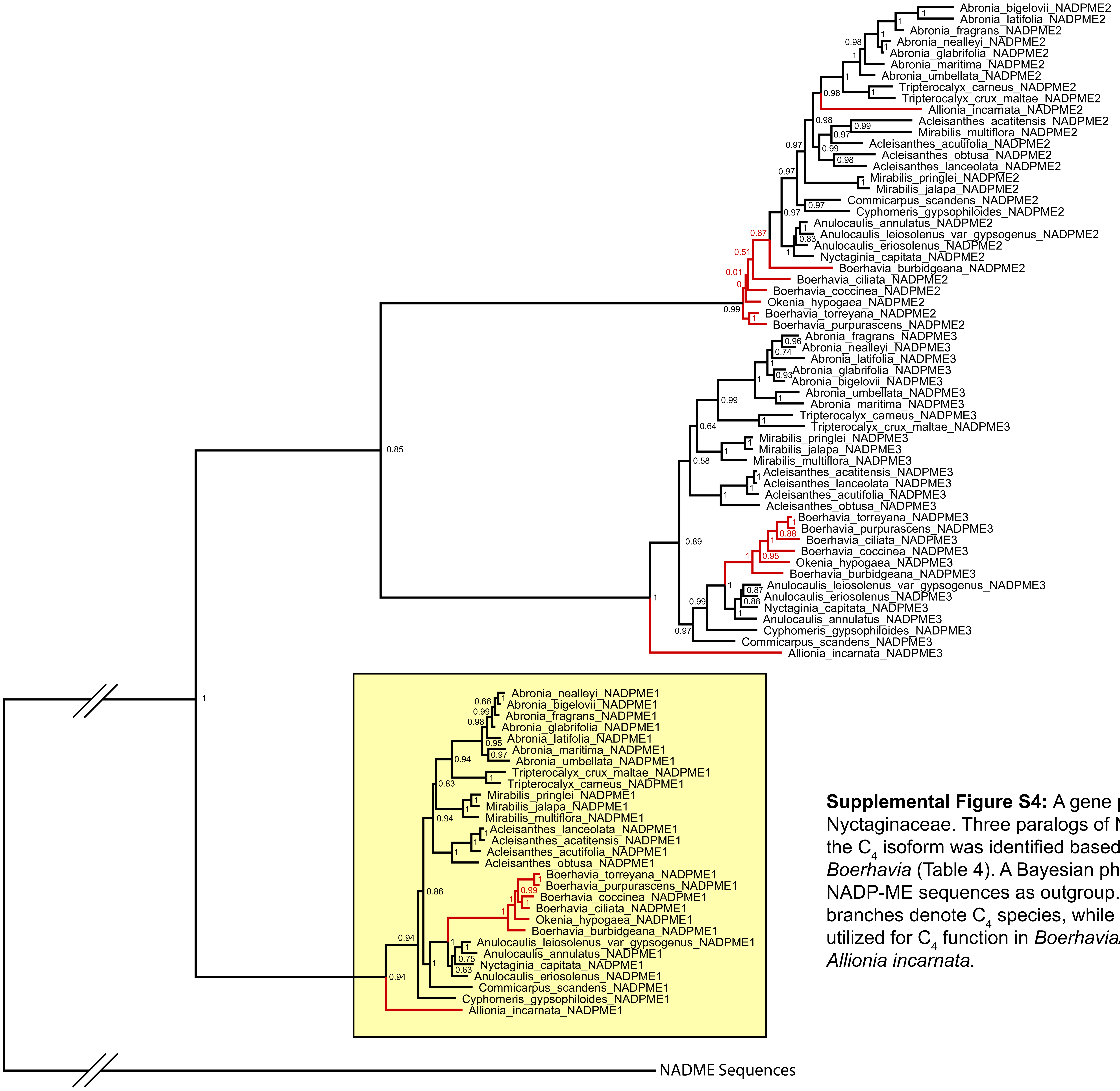

Supplemental Figure S4: A gene phylogeny for NADP malic enzyme in Nyctaginaceae. Three paralogs of NADP-ME were detected in Nytaginaceae, and the C_4 isoform was identified based on having the highest expression in leaves of *Boerhavia* (Table 4). A Bayesian phylogenetic inference was conducted using the NADP-ME sequences as outgroup. Node values are posterior probabilities. Red branches denote C_4 species, while the highlighted box denotes the gene copy utilized for C_4 function in *Boerhavia/Okenia*, which is also significantly expressed in *Allionia incarnata.*

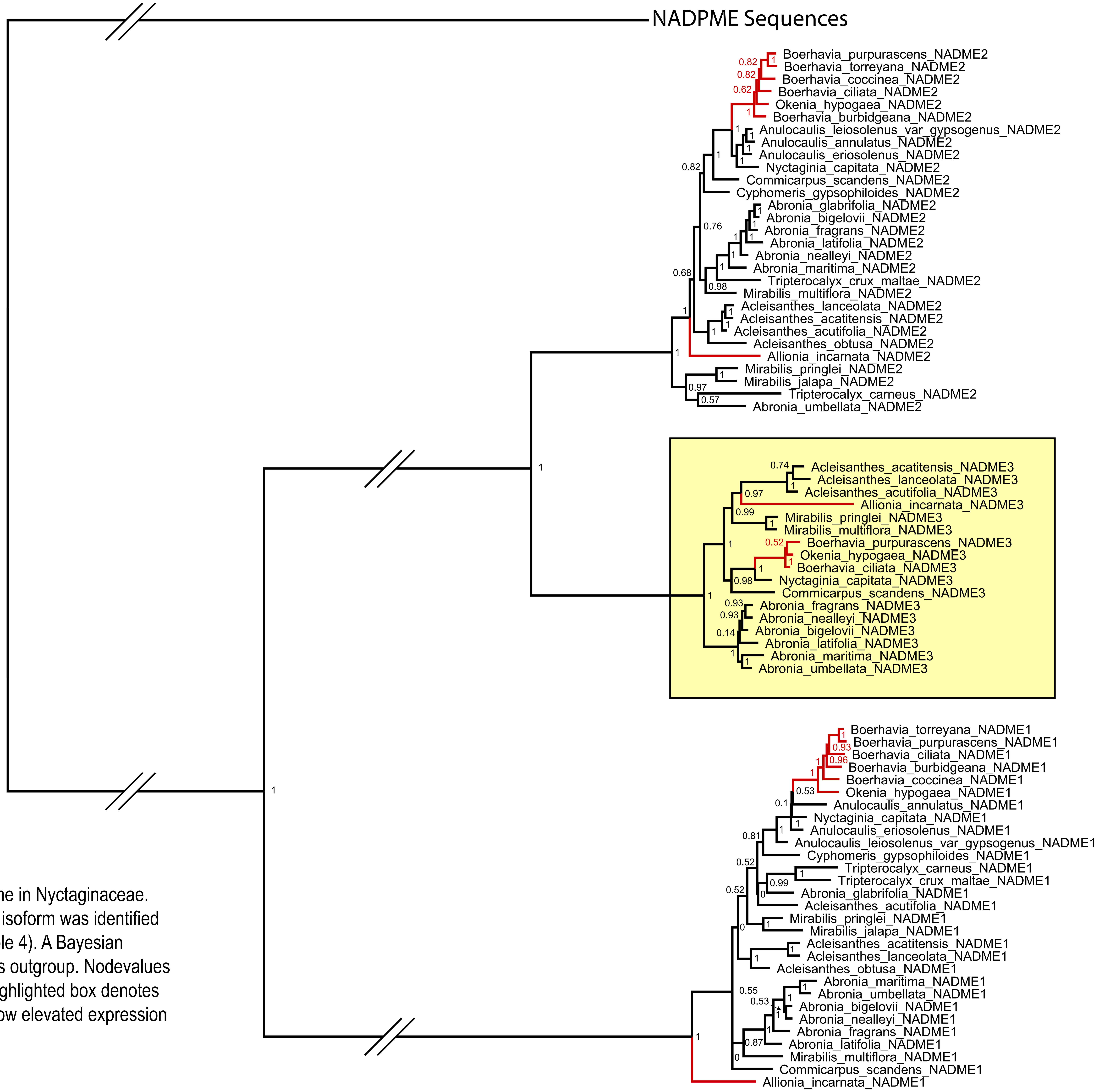

Supplemental Figure S5: A gene phylogeny for NAD malic enzyme in Nyctaginaceae. Three paralogs of NAD-ME were detected in Nyctaginaceae, and the C_4 isoform was identified based on having the highest expression in leaves of *A. incarnata* (Table 4). A Bayesian phylogenetic inference was conducted using the NADPME sequences as outgroup. Nodevalues are posterior probabilities. Red branches denote C_4 species, while the highlighted box denotes the gene copy utilized for C_4 function in *Allionia*, but which does not show elevated expression in *Boerhavia*.

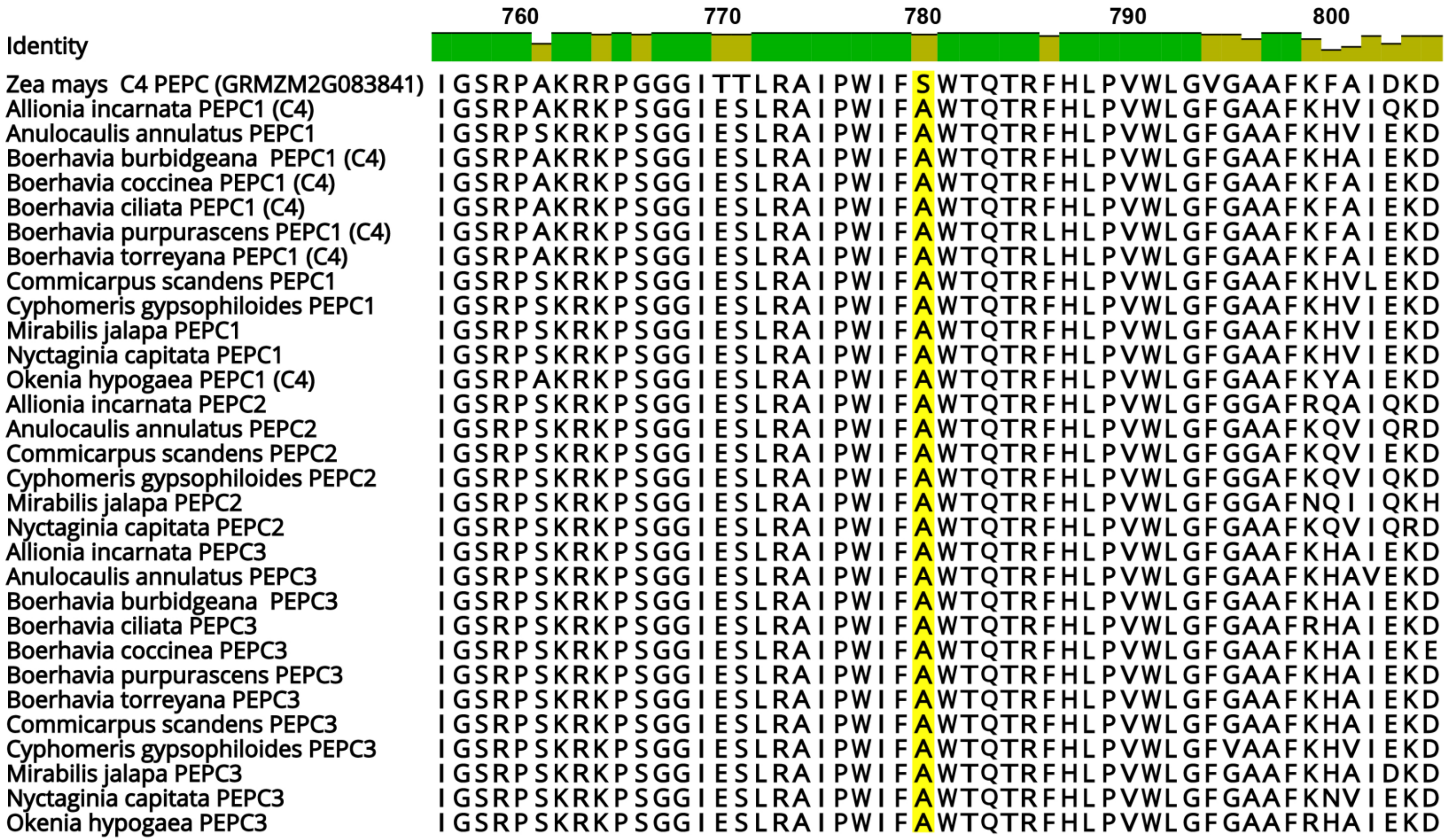

Supplemental Figure S6: Amino acid sequences between positions 755 and 810 for PEP carboxylases from *Zea mays* and numerous species of the Nyctagineae examined in this study. Position 780 is highlighted in yellow, and represents the site of a convergent alanine to serine substitution observed in many C_4 lineages yet absent in C_4 Nyctaginaceae species.

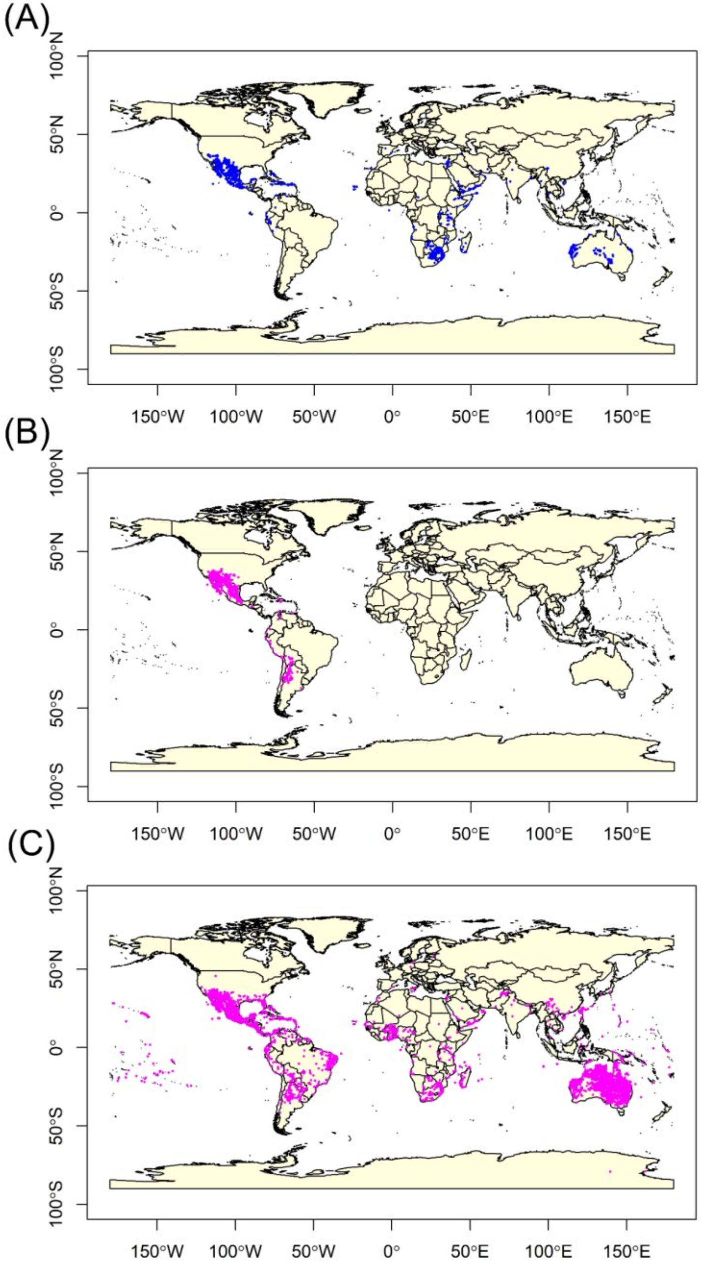

Figure S7: Global distribution map of species in six genera of the Nyctagineae. Panel A, *Boerhavia* species; panel B, *Allionia* species; panel C, species in the C_3 sister genera of the C_4 clades (*Anulocaulis, Commicarpus, Cyphomeris, Nyctaginia)*.

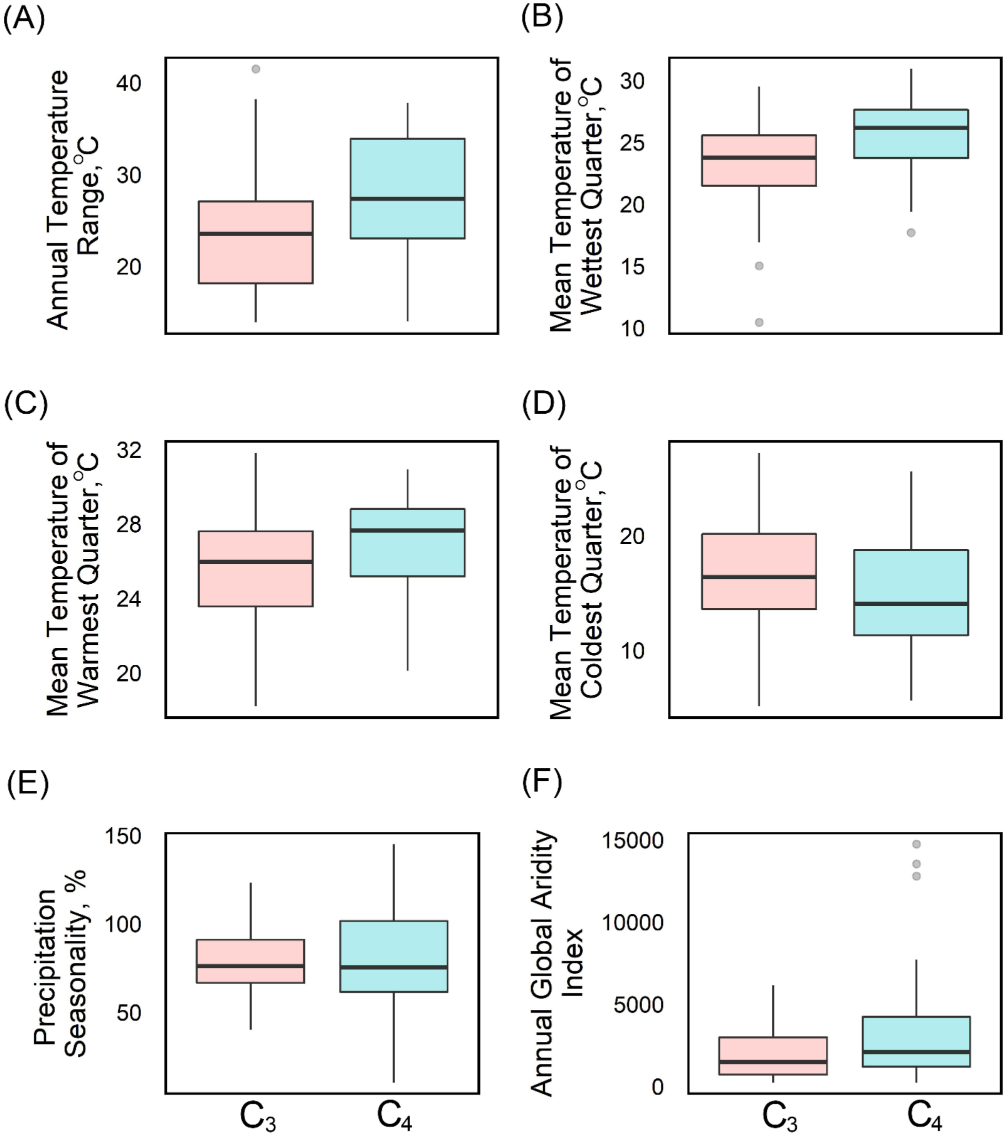

Supplemental Figure S8: Boxplots of climate variables selected by a stepwise regression model using the following variables: panel A, annual temperature range; panel B, mean temperature of wettest quarter, panel C, mean temperature of the warmest quarter, panel D, mean temperature of the coldest quarter; panel E, seasonality of precipitation; panel F, annual global aridity index. The data used was compiled from 15,870 observation for 75 species of Nyctagineae as follows: *Allionia* - two species; *Boerhavia* - 40 species; *Anulocaulis* - five species; *Commicarpus* - 25 species; *Cyphomeris* - two species; and *Nyctaginia* - one specie. Median values per variable per species were used to create the boxplots.

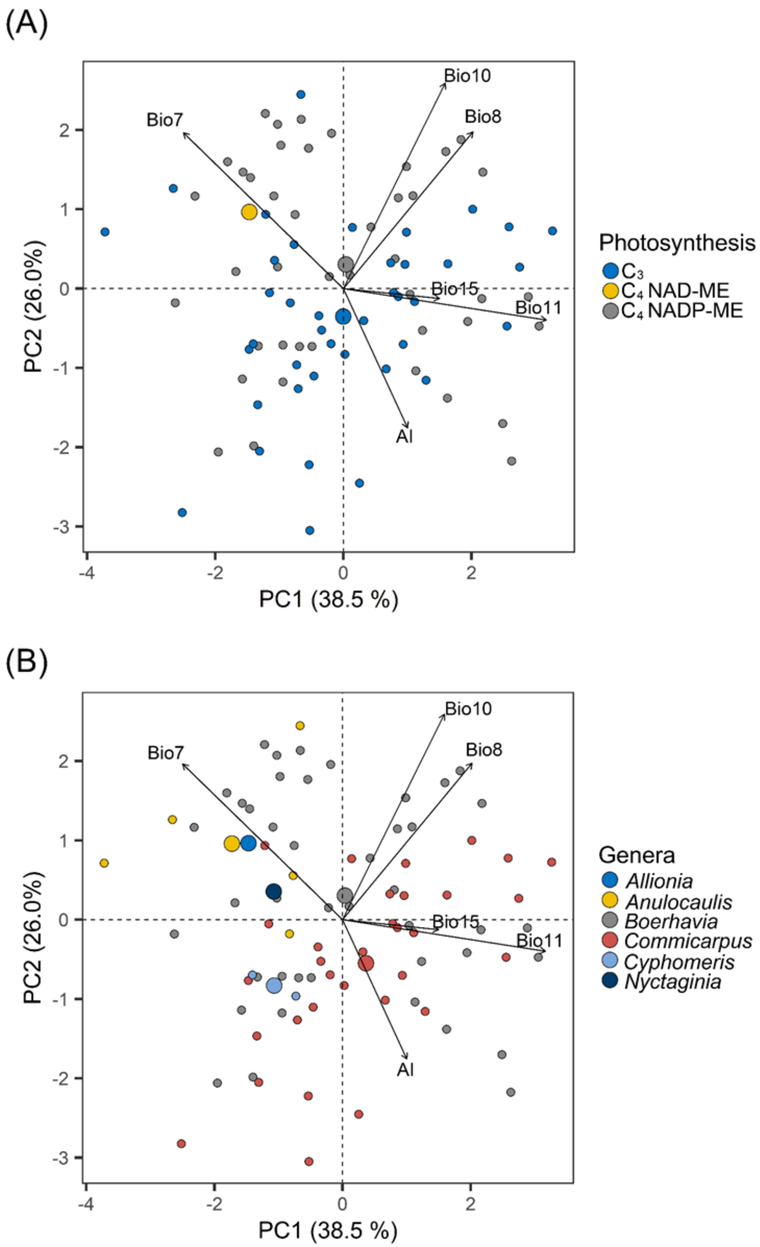

Supplemental Figure S9: Two components of a Principal Component Analysis of selected bioclimate variables for the habitats of C_3 and C_4 species of the Nyctagineae. Data include distributions of C_4 genera *Allionia* (NAD-ME, two species) and *Boerhavia* (NADP-ME, 40 species), and four close C_3 relatives: *Anulocaulis* (five species), *Commicarpus* (25 species), *Cyphomeris* (two species), and *Nyctaginia* (one species). Large symbols indicate mean value when the grouping is by photosynthetic type (panel A) or genus (panel B). Small symbols represents median values of individual species. Bio7, annual temperature range; Bio8, mean temperature of the wettest quarter; Bio10, mean temperature of warmest quarter; Bio11, mean temperature of coldest quarter; Bio15, seasonality of precipitation; AI, annual aridity index.
